# Supplementary material for: Highly mutated antibodies capable of neutralizing N276 glycan-deficient HIV after a single immunization with an Env trimer
Source: Cell Rep. 2022 Mar 8;38(10):110485. doi: 10.1016/j.celrep.2022.110485 (PMC8924373; doi:10.1016/j.celrep.2022.110485)
Supplement: Document S1. Figures S1–S5 [file mmc1.pdf]

Cell Reports, Volume *38*

Supplemental information

Highly mutated antibodies capable of neutralizing N276 glycan-deficient HIV after a single immunization with an Env trimer

Jeong Hyun Lee, Catherine Nakao, Michael Appel, Amber Le, Elise Landais, Oleksandr Kalyuzhniy, Xiaozhen Hu, Alessia Liguori, Tina-Marie Mullen, Bettina Groschel, Robert K. Abbott, Devin Sok, William R. Schief, and Shane Crotty

A

BG505 SOSIP	AENLWVTVYYGVPVWKDAETTLFCASDAKAYETEKHNVWATHACVPTDPNPQEIHLENVTEEFNMWKNNMVEQMHTDIISLWDQSLK
BG505 SOSIP-GT3	AENLWVTVYYGVPVWKDAETTLFCASDAKAYETEKHNVWATHACVPTDPNPQEIHLENVTEEFNMWKNNMVEQMHTDIISLWDQSLK
BG505 MD39-GT3.1	AENLWVTVYYGVPVWKDAETTLFCASDAKAYETEKHNVWATHACVPTDPNPQEIHLENVTEEFNMWKNNMVEQMHEDIISLWDQSLK
BG505 SOSIPv4.1-GT1	AENLWVTVYYGVPVWKDAETTLFCASDAKAYETKKHNVWATHACVPTDPNPQEIHLENVTEEFNMWKNNMVEQMHTDIISLWDQSLK
BG505 SOSIP	PCVKLTPLCVTLQCTNVTNNITDDMRGELKNCSFNMTTELRDKKQKVYSLFYRLDVVQINENQGNRSNNSNKEYRLINCNTSAITQA
BG505 SOSIP-GT3	PCVKLTPLCVTLQCTNVTNNITDDMRGELKNCSFNMTTELRDKKQKVYSLFYRLDVVQINENQGNRSNNSNKEYRLINCNTSAITQA
BG505 MD39-GT3.1	PCVKLTPLCVTLQCTNVTNNITDDMRGELKNCSFNMTTELRDKKQKVYSLFYRLDVVQINENQGNRSNNSNKEYRLINCNTSAITQA
BG505 SOSIPv4.1-GT1	PCVKLTPLCVTLQCTNVTNNITDDMRGELKNCSFNMTTELRDKRQKVHALFYKLDIVPINENQNT-------SYRLINCNTAAITQA
BG505 SOSIP	CPKVSFEPIPIHYCAPAGFAILKCKDKKFNGTGPCPSVSTVQCTHGIKPVVSTQLLLNGSLAEEEVMIRSENITNNAKNILVQFNTP
BG505 SOSIP-GT3	CPKVSFEPIPIHYCAPAGFAILKCKDKKFNGTGPCPSVSTVQCTHGIKPVVSTQLLLNGSLAEEEVMIRSEDFRNNAKNILVQFNTP
BG505 MD39-GT3.1	CPKVSFEPIPIHYCAPAGFAILKCKDKKFNGTGPCPSVSTVQCTHGIKPVVSTQLLLNGSLAEEEVIIRSEDFRNNAKNILVQLNTP
BG505 SOSIPv4.1-GT1	CPKVSFEPIPIHYCAPAGFAILKCKDKKFNGTGPCPSVSTVQCTHGIKPVVSTQLLLNGSLAEEEVMIRSEDIRNNAKNILVQFNTP
BG505 SOSIP	VQINCTRPNNNTRKSIRIGPGQAFYATGDIIGDIRQAHCNVSKATWNETLGKVVKQLRKHFGNNTIIRFANSSGGDLEVTTHSFNCG
BG505 SOSIP-GT3	VQINCTRPNNNTRKSIRIGPGQAFYATGDIIGDIRQAHCNVSKATWNETLGKVVKQLRKHYGNDTTIRFAPSSGGDLEVMNHSFNCG
BG505 MD39-GT3.1	VQINCTRPNNNTVKSIRIGPGQAFYYTGDIIGDIRQAHCNVSKATWNETLGKVVKQLRKHYGNDTTIRFAPSSGGDLEVMNHSFNCG
BG505 SOSIPv4.1-GT1	VQINCTRPNNNTRKSIRIGPGQWFYATGDIIGDIRQAHCNVSKATWNETLGKVVKQLRKHFGNNTIIRFANSSGGDLEVTTHSFNCG
BG505 SOSIP	GEFFYCNTSGLFNSTWISNTSVQGSNSTGSNDSITLPCRIKQIINMWQRIGQAMYAPPIQGVIRCVSNITGLILTRDGGSTNSTTET
BG505 SOSIP-GT3	GEFFYCNTSGLFNSTWISNTSVQGSNSTGSNDSITLPCRIKQIINMWQRIGQAMYAPPIQGVIRCVSNITGLILVRDGGYTNSNTET
BG505 MD39-GT3.1	GEFFYCNTSGLFNSTWISNTSVQGSNSTGSNDSITLPCRIKQIINMWQRIGQAMYAPPIQGVIRCVSNITGLILVRDGGYTNSNTET
BG505 SOSIPv4.1-GT1	GEFFYCDTSGLFNSTWISNTSVQGSNSTGSNDSITLPCRIKQIINMWQRIGQAMYAPPIQGVIRCVSNITGLILTRDGGSTDSTTET
BG505 SOSIP	FRPGGGDMRDNWRSELYKYKVVKIEPLGVAPTRCKRRVVG**RRRRRR**AVGIGAVFLGFLGAAGSTMGAASMTLTVQARNLLSGIVQQQ
BG505 SOSIP-GT3	FRPGGGDMRDNWRSELYKYKVVKIEPLGVAPTRCKRRVVG**RRRRRR**AVGIGAVFLGFLGAAGSTMGAASMTLTVQARNLLSGIVQQQ
BG505 MD39-GT3.1	FRPGGGDMRDNWRSELYKYKVVKIEPLGVAPTRCKRRVVG**RRRRRR**AVGIGAVSLGFLGAAGSTMGAASMTLTVQARNLLSGIVQQQ
BG505 SOSIPv4.1-GT1	FRPSGGDMRDNWRSELYKYKVVKIEPLGVAPTRCKRRVVG**RRRRRR**AVGIGAVFLGFLGAAGSTMGAASMTLTVQARNLLSGIVQQQ
BG505 SOSIP	SNLLRAPEAQQHLLKLTVWGIKQLQARVLAVERYLRDQQLLGIWGCSGKLICCTNVPWNSSWSNRNLSEIWDNMTWLQWDKEISNYT
BG505 SOSIP-GT3	SNLLRAPEAQQHLLKLTVWGIKQLQARVLAVERYLRDQQLLGIWGCSGKLICCTNVPWNSSWSNRNLSEIWDNMTWLQWDKEISNYT
BG505 MD39-GT3.1	SNLLRAPEPQQHLLKDTHWGIKQLQARVLAVEHYLRDQQLLGIWGCSGKLICCTNVPWNSSWSNRNLSEIWDNMTWLQWDKEISNYT
BG505 SOSIPv4.1-GT1	SNLLRAPEAQQHLLKLTVWGIKQLQARVLAVERYLRDQQLLGIWGCSGKLICCTNVPWNSSWSNRNLSEIWDNMTWLQWDKEISNYT
BG505 SOSIP	QIIYGLLEESQNQQEKNEQDLLALD
BG505 SOSIP-GT3	QIIYGLLEESQNQQEKNEQDLLALD
BG505 MD39-GT3.1	QIIYGLLEESQNQQEKNEQDLLALD
BG505 SOSIPv4.1-GT1	QIIYGLLEESQNQQEKNEQDLLALD

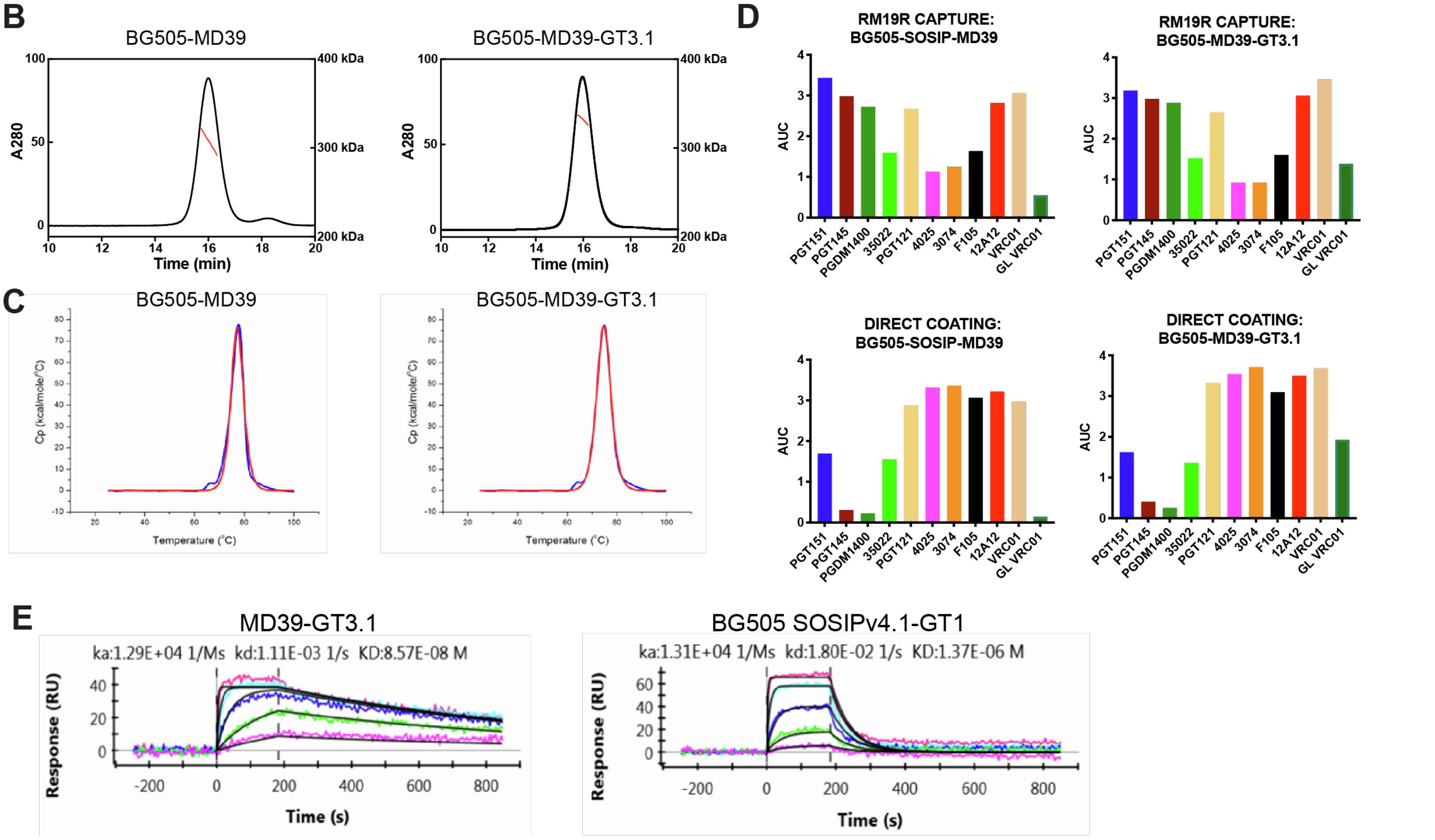

Figure S1. Characterization of the MD39-GT3.1 trimer immunogen and related immunogens, related to Figures 1 and 2.

(A) The sequence of BG505 SOSIP trimer and BG505 SOSIP-based CD4bs GT trimer constructs. Modifications from the parent BG505 SOSIP sequence is shown in red. The furin cleavage site between gp120 and gp41 is shown in bold font. The 15mer epitope of HYCAP Tg CD4 T cells is underlined.
(B) Size exclusion chromatography-multiangle light scattering (SEC-MALS) traces for BG505 MD39 and BG505 MD39-GT3.1. The left y-axis is normalized A280 and right y-axis is the molecular weight of protein and glycan. The MW assessed by protein-conjugate analysis is shown as the red line inside the peak. Theoretical protein MW is 214 kDa in both cases.
(C) Thermal stability of BG505 MD39 and BG505 MD39-GT3.1 measured by differential scanning calorimetry (DSC). The raw data are shown as blue solid line, and the fit is shown as red line. Melting temperature (Tm) values from the fit are shown.
(D) Antigenic profiles measured by ELISA for BG505 MD39 and BG505 MD39-GT3.1, with trimers either captured by the base binding mAb RM19R (Cottrell et al., 2020) in order to preserve trimer quaternary structure or coated directly onto the ELISA plate so as to disrupt trimer quaternary structure.
(E) SPR kinetic analysis of glVRC01 Fab binding to MD39-GT3.1 trimer (left) and BG505 SOSIPv4.1-GT1 (right) captured on sensor by PGT121. The glVRC01 Fab analyte was tested at a top concentration of 30.347 μM and four successive 4-fold dilutions. The dissociation constant from this kinetic fit was 86 nM and1.4 μM, respectively.

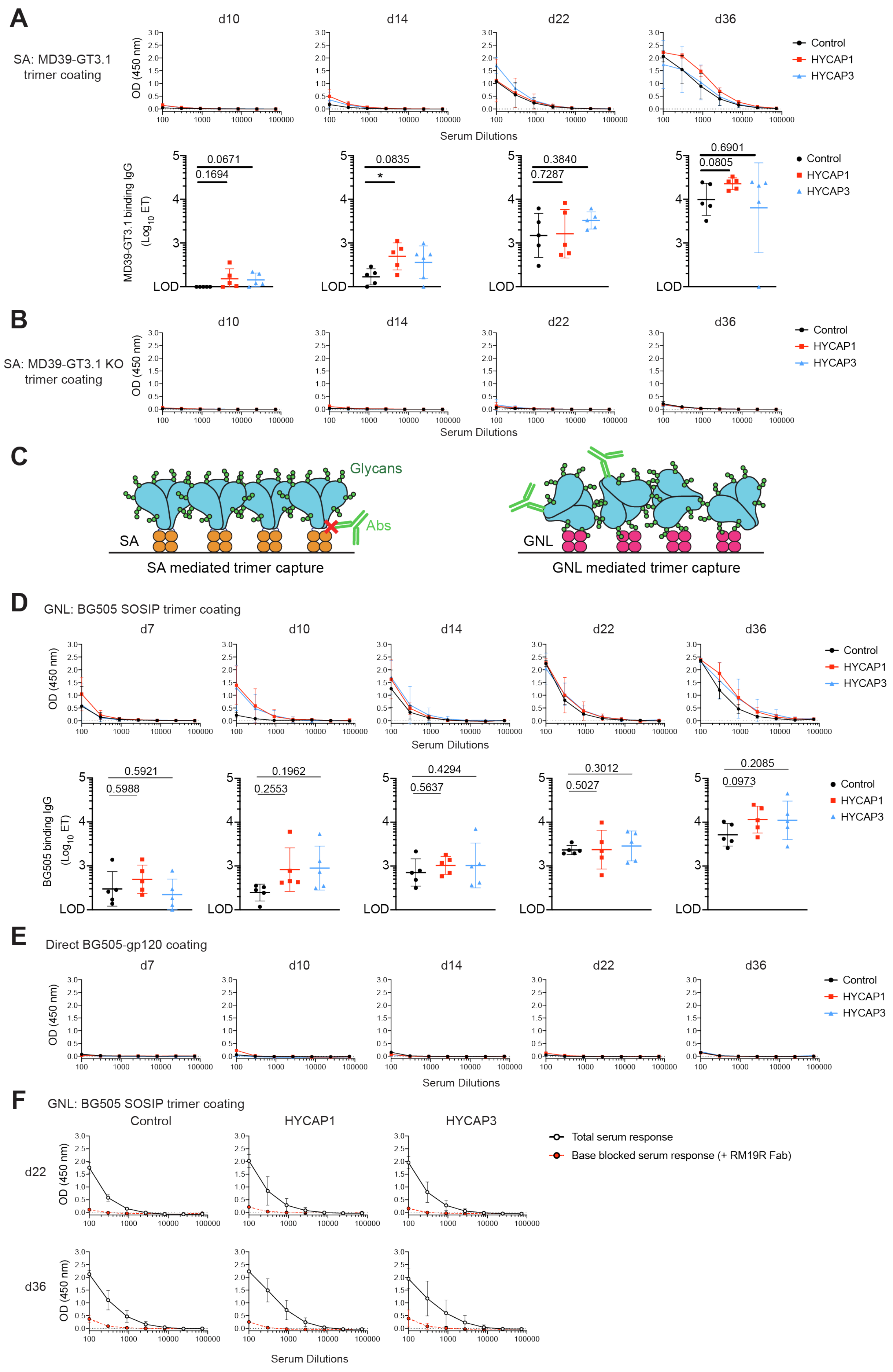

Figure S2. Serum IgG responses from MD39-GT3.1 immunized mice, related to Figure 2.

(A) MD39-GT3.1 trimer-binding serum IgG measured by ELISA and endpoint titers (ET). Biotinylated MD39-GT3.1 trimers were captured on to SA coated plates.
(B) As in (A), but serum IgG binding to the MD39-GT3.1 KO trimer.
(C) Comparison of SA-biotinylated trimer capture and GNL-trimer capture. SA-based trimer capture results in C-terminus oriented binding of the trimer on to ELISA plates, sterically occluding the trimer base epitope. GNL binds mannose residues and captures Env trimers in random orientations.
(D) BG505 SOSIP trimer-binding serum IgG measured by ELISA and ET. Plates were initially coated with GNL, and trimer was captured onto the GNL coated plates. P-values calculated by two-tailed Student's t-tests.
(E) IgG serum ELISA to detect monomeric BG505 gp120. Recombinant BG505 gp120 was directly coated onto ELISA plates.
(F) As in (D) for d22 and d36 (black lines), but with the respective trimer base blocking ELISA performed in parallel (red lines). To block the base epitope, RM19R Fab was added to the trimers prior to the addition of mouse serum.

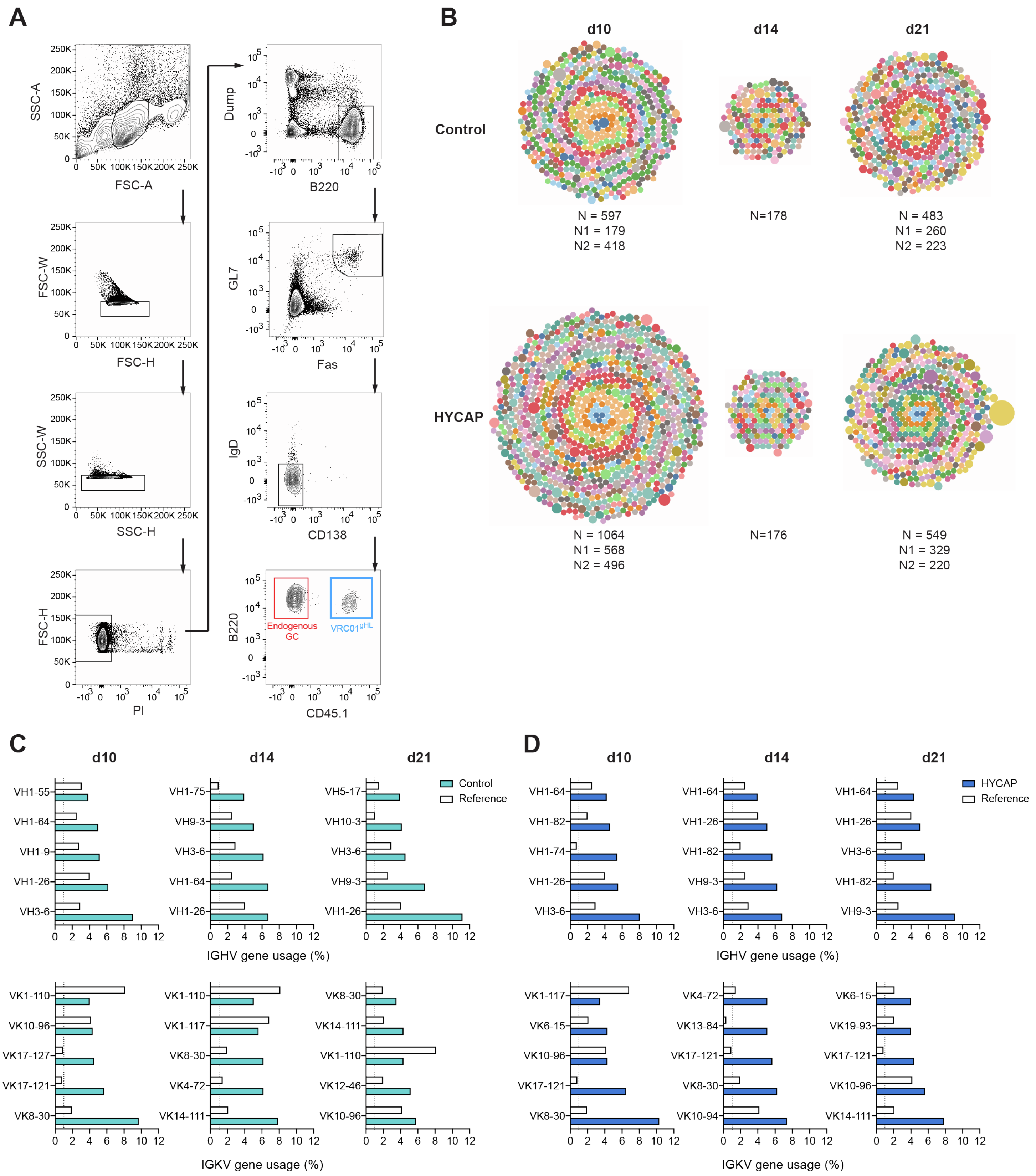

Figure S3. Analysis of endogenous B_{GC} BCR sequences in MD39-GT3.1 trimer immunized mice, related to Figure 2.

(A) Endogenous B_{GC} cell and VRC01^{gHL} B_{GC} cell sort strategy for sequencing. Dump: CD4, CD8a, NK1.1, Gr-1.

(B) Diversity of endogenous B_{GC} cells. Each circle denotes a unique clonotype, defined by paired HC VDJ-LC VJ genes. The size of each circle is directly proportional to the number of clones observed for that clonotype. Circles are colored by IGHV-genes although the colors are not mutually exclusive due to the large number of IGHV genes observed. Distinct

clones using the same IGHV-genes are proximally clustered. N = total number of paired sequences obtained from two independent experiments; N1 = sequences from first experiment, pooled from 4 mice each for both control and HYCAP3 groups; N2 = sequences from a second experiment, pooled from 5 mice in the control group, pooled from 10 mice in the HYCAP group (5 mice from HYCAP1 and HYCAP3 groups each). B cells from d14 were only sequenced in the second experiment.
(C) The top 5 most used IGHV (VH, upper) and IGKV (VK, lower) genes among HC-LC paired endogenous B_{GC} cells after 10, 14 or 21 days post immunization in the control group. The reference V-gene usage frequencies were derived from V-gene usage observed unimmunized C57BL/6J splenic B cells (Rettig et al., 2018). Dotted line indicates 1%.
(D) As in (C) but analysis of sequences from mice that received HYCAP1 and HYCAP3 CD4 T cells by adoptive transfer.

A

	Control	N1	N2	N1+N2	HYCAP1	N1	N2	N1+N2	HYCAP3	N1	N2	N1+N2	Total
d10	HC	5	89	94	HC	0	221	221	HC	312	242	554	869
	LC	3	49	52	LC	0	142	142	LC	193	145	338	532
d14	HC	0	117	117	HC	0	201	201	HC	0	215	215	533
	LC	0	100	100	LC	0	146	146	LC	0	165	165	411
d21	HC	43	158	201	HC	0	283	283	HC	97	261	358	842
	LC	46	129	175	LC	0	214	214	LC	93	209	302	691
d36	HC	0	133	133	HC	0	131	131	HC	0	117	117	381
	LC	0	113	113	LC	0	96	96	LC	0	83	83	292

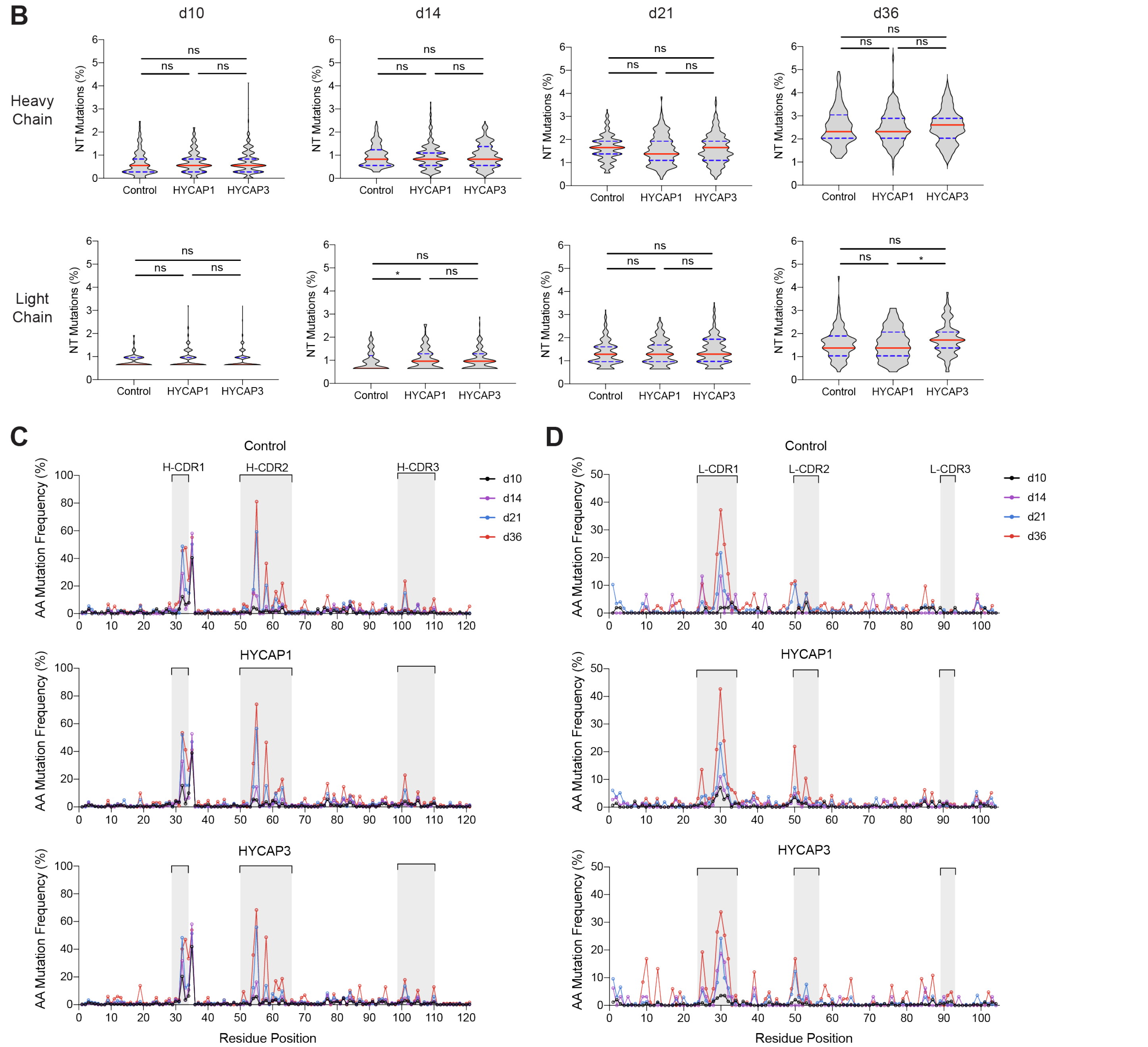

Figure S4. Sequencing of VRC01^{gHL} cells, related to Figure 3.

(A) Number of B_{GC} VRC01^{gHL} HC and LC sequences analyzed. N1 and N2 indicate two independent experiments. D10, 14, 21 B cells were sequenced using the 10X Genomics platform and pooled from 4 mice per group for each experiment. D36 sequences were obtained by Sanger sequencing from 4 mice within each transfer group.

(B) Violin plots of % nucleotide (NT) mutations for the data shown in Figure 2A. The denominator for d10, 14, 21 sequences is 363 NT residues for HC and 312 residues for LC. The denominator for d36 sequences is 345 NT residues for HC and 290 residues for LC.

(C) Per residue HC AA mutations in the three experimental groups.
(D) Per residue LC AA mutations in the three experimental groups.

			M1		M2		M3		M4		M5		M6		M7		M8		M9	
Virus Strain	Tier	Clade	T	S	T	S	T	S	T	S	T	S	T	S	T	S	T	S	T	S
191084 B7-19_N276A	2	A	0.02631	0.00295	0.02851	0.00276	1.07981	0.05221	0.02356	0.00146	26.43695	0.11549	9.25587	0.06637	1.24439	0.00542	0.07767	0.00225	0.23643	0.00289
BG505_N276A	2	A	3.87232	NT	21.55737	NT	17.87130	NT	8.80939	NT	NN	NT	66.33857	NT	76.17481	NT	0.53851	NT	79.94976	NT
BG505_N276D	2	A	3.06971	NT	40.76442	NT	3.46174	NT	0.49062	NT	95.17247	NT	17.85259	NT	3.84156	NT	0.05780	NT	1.14787	NT
REJO4541.67_N276A	2	B	3.79945	0.01394	44.31766	0.10066	11.37000	0.39231	8.65472	0.01772	NN	NN	NN	22.88683	NN	NN	NN	38.42655	NN	93.06217
1012_11_TC21_3257_N276A	1B	B	0.12607	0.02426	0.44165	0.01760	6.86379	0.22708	7.33115	0.09931	NN	95.86216	NN	37.24879	15.54891	0.45328	5.41503	0.03266	21.41162	0.11809
HIV-001428-2.42_N276A	2	C	0.01680	0.00082	7.89591	0.01182	2.64889	0.03082	0.60076	0.00297	NN	15.01911	21.68769	0.15419	19.81655	0.14983	0.04261	0.00057	1.20000	0.00210
3301.v1.c24_N276A		AC	NN	0.06797	NN	0.09463	NN	0.46500	NN	0.09445	NN	1.07127	NN	6.92608	NN	2.79699	NN	0.03651	NN	0.02381
6041.v3.c23_N276A		AC	NN	0.62301	NN	NN	NN	NN	NN	5.48987	NN	NN	NN	NN	NN	NN	29.68087	0.11642	NN	5.32711
92TH021_N276A	2	AE	NN	0.13069	NN	NN	NN	NN	NN	NN	NN	NN	NN	NN	NN	NN	NN	0.49901	NN	NN
191084 B7-19_N276D	2	A	0.07461	0.00304	0.04816	0.00324	0.25399	0.00722	0.02596	0.00212	0.95622	0.01078	0.11903	0.00379	0.04188	0.00283	0.02837	0.00215	0.04985	0.00337
SC422661.8_N276D	2	B	1.72409	NT	3.75924	NT	8.09772	NT	0.32041	NT	NN	NT	NN	NT	24.88707	NT	16.12163	NT	78.30517	NT
HIV-001428-2.42_N276D	2	C	0.02598	0.00085	15.10990	0.01596	1.14572	0.00733	0.16154	0.00250	NN	2.77310	34.96976	0.14733	37.50414	0.03775	0.01913	0.00037	0.23609	0.00125
HIV-0815.v3.c3_N276D	2	ACD	4.12250	NT	NN	NT	NN	NT	2.30058	NT	50.56341	NT	NN	NT	NN	NT	0.73764	NT	NN	NT
MLV			NN	NN	NN	NN	NN	NN	NN	NN	NN	NN	NN	NN	NN	NN	NN	NN	NN	NN

			M10		M11		M12		M13		M14		M15		E7		E11		glVRC01		VRC01	
Virus Strain	Tier	Clade	T	S	T	S	T	S	T	S	T	S	T	S	T	S	T	S	T	S	T	S
191084 B7-19_N276A	2	A	5.50763	0.02893	9.50712	0.03078	0.05328	0.00272	0.00746	0.00049	NN	79.73813	NN	28.77998	2.77038	0.02073	0.02364	0.00149	NN	NN	0.03100	0.00092
BG505_N276A	2	A	24.89845	NT	NN	NT	NN	NT	1.00591	NT	NN	ND	NN	ND	NN	NT	NN	NT	NN	ND	0.01300	NT
BG505_N276D	2	A	0.10831	NT	0.64819	NT	3.72484	NT	0.00850	NT	NN	ND	NN	ND	30.75698	NT	15.40579	NT	NN	ND	0.01100	NT
REJO4541.67_N276A	2	B	NN	63.44921	NN	NN	NN	0.39616	0.00805	0.00001	NN	NN	NN	NN	27.23893	0.50054	16.35902	0.57058	NN	NN	0.02100	0.00350
1012_11_TC21_3257_N276A	1B	B	NN	NN	NN	21.65742	0.28868	0.03467	0.03405	0.00393	NN	NN	NN	NN	89.60507	6.88754	0.80414	0.01075	NN	NN	0.05400	0.81000
HIV-001428-2.42_N276A	2	C	NN	0.53909	12.40000	0.01435	0.53000	0.00155	0.01100	0.00002	NN	NN	NN	2.46747	NN	2.00477	ND	0.94445	NN	NN	0.00550	0.00240
3301.v1.c24_N276A		AC	NN	39.31180	NN	0.76253	NN	0.03244	0.20735	0.00001	NN	NN	NN	12.53858	NN	1.56525	NN	0.13680	NN	NN	0.09000	0.00340
6041.v3.c23_N276A		AC	NN	7.14739	NN	NN	NN	NN	NN	2.56698	NN	NN	NN	NN	NN	NN	NN	NN	NN	NN	0.00900	0.00290
92TH021_N276A	2	AE	NN	NN	NN	NN	NN	NN	NN	2.80218	NN	NN	NN	NN	NN	NN	NN	NN	NN	NN	0.06000	0.01000
191084 B7-19_N276D	2	A	0.11201	0.00255	0.14182	0.00439	0.01806	0.00262	0.00795	0.00024	NN	1.01321	NN	0.29781	0.02558	0.00074	0.01150	0.00127	NN	36.73057	0.02700	0.00520
SC422661.8_N276D	2	B	35.10128	NT	NN	NT	0.25366	NT	0.00585	NT	NN	ND	NN	ND	7.62172	NT	4.75373	NT	NN	ND	0.01000	NT
HIV-001428-2.42_N276D	2	C	1.79904	0.00369	0.45619	0.00137	0.01341	0.00123	0.00114	0.00001	NN	99.53962	NN	1.30816	2.16156	0.00392	5.18986	0.01404	NN	NN	0.01000	0.00230
HIV-0815.v3.c3_N276D	2	ACD	4.16580	NT	9.12986	NT	NN	NT	0.04374	NT	NN	ND	NN	ND	NN	NT	NN	NT	NN	ND	0.00780	NT
MLV			NN	NN	NN	NN	NN	NN	NN	NN	NN	NN	NN	NN	NN	NN	NN	NN	NN	NN	NN	NN

Figure S5. Pseudovirus neutralization by post-immunization VRC01^{gHL} mAbs, related to Figure 6.
Individual pseudovirus neutralization assay IC_{50} (μg/mL) values graphed in Figure 6. The indicated ΔN276 viruses were produced in HEK293T (T) or HEK293S (S) cells. Murine leukemia virus (MLV) was used as a negative control. ND = Not determined; NN = IC_{50} >100 μg/mL, no neutralization; NT = No virus titer. Data shown is an average of two experiments.
