# Supplementary material for: Highly mutated antibodies capable of neutralizing N276 glycan-deficient HIV after a single immunization with an Env trimer
Source: Cell Rep. 2022 Mar 8;38(10):110485. doi: 10.1016/j.celrep.2022.110485 (PMC8924373; doi:10.1016/j.celrep.2022.110485)
Supplement: Document S2. Article plus and supplemental information [file mmc3.pdf]

Cell Reports

Article

Highly mutated antibodies capable of neutralizing N276 glycan-deficient HIV after a single immunization with an Env trimer

Graphical abstract

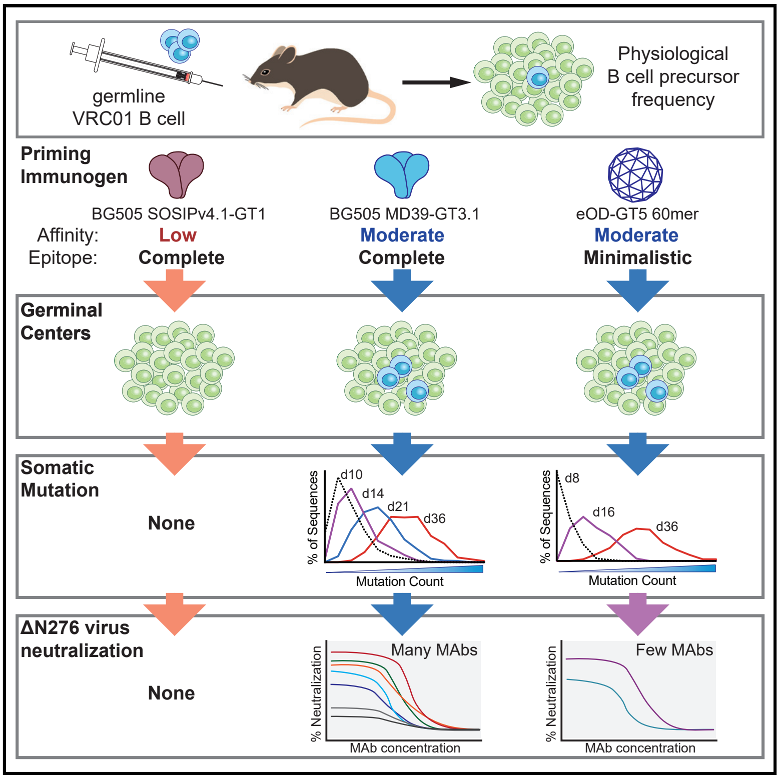

Highlights

- VRC01-class naive B cells can acquire many bnAb-like mutations after 1 immunization
- VRC01-class Abs neutralizing multiple ΔN276 HIV pseudoviruses were elicited
- Trimer-based but not minimal GT immunogen priming elicited key trimer-binding SHMs

Authors

Jeong Hyun Lee, Catherine Nakao, Michael Appel, ..., Devin Sok, William R. Schief, Shane Crotty

Correspondence

shane@lji.org (S.C.),
schief@scripps.edu (W.R.S.)

In brief

Protective broadly neutralizing antibodies (bnAbs) to HIV are difficult to elicit by vaccination because they require mutations that have been observed to take years in HIV-infected humans. Lee et al. show that several key mutations required for Env trimer binding and bnAb development can be acquired after a single immunization.

Lee et al., 2022, Cell Reports *38*, 110485
March 8, 2022 © 2022 The Author(s).
https://doi.org/10.1016/j.celrep.2022.110485

CellPress

Cell Reports

CellPress OPEN ACCESS

Article

Highly mutated antibodies capable of neutralizing N276 glycan-deficient HIV after a single immunization with an Env trimer

Jeong Hyun Lee,[1,2] Catherine Nakao,[1] Michael Appel,[3] Amber Le,[3] Elise Landais,[2,3,5] Oleksandr Kalyuzhniy,[2,3,5] Xiaozhen Hu,[2,3,5] Alessia Liguori,[2,3,5] Tina-Marie Mullen,[2,3,5] Bettina Groschel,[2,3,5] Robert K. Abbott,[1,2] Devin Sok,[2,3,4] William R. Schief,[2,3,4,5,*] and Shane Crotty[1,2,6,7,*]

[1]Center for Infectious Disease and Vaccine Research, La Jolla Institute for Immunology (LJI), La Jolla, CA, USA
[2]Consortium for HIV/AIDS Vaccine Development, The Scripps Research Institute, La Jolla, CA 92037, USA
[3]International AIDS Vaccine Initiative Neutralizing Antibody Center, The Scripps Research Institute, La Jolla, CA 92037, USA
[4]Department of Immunology and Microbiology, The Scripps Research Institute, La Jolla, CA 92037, USA
[5]Ragon Institute of Massachusetts General Hospital, Massachusetts Institute of Technology and Harvard University, Cambridge, MA 02139, USA
[6]Division of Infectious Diseases and Global Public Health, Department of Medicine, University of California, San Diego (UCSD), La Jolla, CA 92037, USA
[7]Lead contact
*Correspondence: shane@lji.org (S.C.), schief@scripps.edu (W.R.S.)
https://doi.org/10.1016/j.celrep.2022.110485

SUMMARY

Elicitation of HIV broadly neutralizing antibodies (bnAbs) is challenging because unmutated bnAb precursors are rare and seldom bind HIV envelope glycoprotein (Env) trimers. One strategy to initiate bnAb responses is to use germline-targeting (GT) immunogens with high affinity to bnAb-class precursor B cells and then shepherd affinity maturation with booster immunogens that successively look more like native Env. In a mouse model where the frequency of VRC01-precursor (VRC01^{gHL}) B cells mimics that of humans, we show that following a GT HIV Env trimer protein prime, VRC01-class B cells in the germinal center (GC) acquire high-affinity VRC01-class B cell somatic hypermutations (SHMs). Many GC-derived VRC01^{gHL} antibodies robustly bind N276 glycan-deficient Env trimers and neutralize several N276 glycan-deficient tier 2 HIV strains. These results are encouraging for GT Env trimer vaccine designs and demonstrate accumulation of substantial SHMs, including deletions, uncommon point mutations, and functional bnAb features, after a single immunization.

INTRODUCTION

Discovery of an efficacious vaccine against the human immunodeficiency virus (HIV) has been difficult because of the high mutability and diversity of HIV. For an antibody-based HIV vaccine to offer protection, it would need to elicit broadly neutralizing antibodies (bnAbs) that can potently neutralize a wide range of primary HIV isolates. HIV bnAbs are uncommon at least in part because naive B cells with bnAb-class epitope specificities are rare (Briney et al., 2019; Havenar-Daughton et al., 2018; Jardine et al., 2016a; Steichen et al., 2019) and have features atypical of other antibodies, such as unusually long heavy-chain (HC) complementarity-determining regions (CDRs) (Doria-Rose et al., 2014; Falkowska et al., 2014; Freund et al., 2017; Landais et al., 2017; MacLeod et al., 2016; Sok et al., 2014; Walker et al., 2009, 2011) or a short light-chain (LC) CDR loop (Zhou et al., 2013). Potent bnAbs are typically found in individuals chronically infected with HIV after years of infection and have more somatic hypermutations (SHMs) than usually elicited in response to vaccinations (Jardine et al., 2016b; Sok and Burton, 2018). Some key mutations in bnAbs are not commonly observed in typical antibodies, including insertions, deletions, and disulfide bonds (Doria-Rose et al., 2014; Landais and Moore, 2018; Zhou et al., 2010).

An HIV vaccine research approach called structure-based rational vaccine design strives to design immunogens that recapitulate naturally occurring bnAb responses (Burton, 2017; Stamatatos et al., 2017). One strategy involves priming with an immunogen that has a high binding affinity for predicted naive precursors of known bnAb-class B cells (Jardine et al., 2013). Theoretically, these germline-targeting (GT) immunogens would activate rare bnAb precursor B cells and induce germinal center (GC) responses (Mesin et al., 2016) to expand the population of bnAb precursors and initiate affinity maturation. Booster immunizations are administered sequentially to deliver immunogens more closely resembling the native Env trimer, guiding acquisition of SHMs needed for breadth and potency (Briney et al., 2016; Escolano et al., 2016; Jardine et al., 2016b; Steichen et al., 2016; Tian et al., 2016).

One broadly neutralizing epitope that is promising for epitope-specific vaccine design is the CD4 binding site (CD4bs). As the receptor binding site on Env, the CD4bs is highly conserved across HIV isolates, and bnAbs recognizing this epitope have

© 2022 The Author(s).
This is an open access article under the CC BY license (http://creativecommons.org/licenses/by/4.0/).

been identified in multiple HIV-positive donors (Bonsignori et al., 2016; Huang et al., 2016; Kong et al., 2016; Umotoy et al., 2019; Zhou et al., 2015). VRC01 is a representative CD4bs bnAb with high breadth and potency. VRC01-class bnAbs utilize an IGHV1-2 HC gene that is found in approximately 2%–4% of the human B cell repertoire (Arnaout et al., 2011; DeKosky et al., 2015). Epitope recognition by VRC01-class bnAbs relies on several conserved features arising from their IGHV1-2*02 gene. The HC CDR3 (H-CDR3) can be variable but uses a tryptophan residue at the fifth to the last residue (Trp100B in VRC01). Various LC V-genes are observed but have a short, 5-amino acid (aa) L-CDR3 (Diskin et al., 2011; Huang et al., 2016; Umotoy et al., 2019; West et al., 2012; Zhou et al., 2015). Strategies to elicit VRC01-class bnAbs by vaccination have been investigated extensively (Briney et al., 2016; Chen et al., 2021; Duan et al., 2018; Jardine et al., 2013, 2016a; McGuire et al., 2014; Stamatatos et al., 2017). Several GT immunogens exist for VRC01-class bnAbs, at least two of which are currently being tested in phase 1 clinical trials (ClinicalTrials.gov: NCT03547245, NCT04224701; Jardine et al., 2016a; Medina-Ramírez et al., 2017). If rare VRC01-class naive precursors can be primed, then one of the biggest hurdles of achieving neutralization potency and breadth is accommodation of a conserved N-linked glycan at position N276 of Env near the CD4bs. To do so, VRC01-class bnAbs typically introduce glycine residues and/or acquire deletions in the L-CDR1 to promote loop flexibility (Zhou et al., 2015) or expand the epitope contact surface area via H-CDR3 to enhance binding (Bonsignori et al., 2018). Animal immunization models to assess VRC01-class B cell targeting immunogens have shown that the ability to accommodate the N276 glycan does not develop readily (Briney et al., 2016; Chen et al., 2021; Parks et al., 2019; Tian et al., 2016). The question of which set of prime-boost immunogens would best drive breadth-inducing SHMs is still under investigation.

For an HIV-1 vaccine to be practical, the vaccination regimen needs to generate bnAbs faster than observed in HIV-infected individuals, which can take 2–8 years (Burton and Hangartner, 2016). Breadth and potency also need to be achievable with a reasonable number of immunizations (Jardine et al., 2016b). However, many animal immunization studies administer 6–8 shots in total over a time span of up to 1 year (Chen et al., 2021; Escolano et al., 2016; Kong et al., 2019; Saunders et al., 2019; Tian et al., 2016; Xu et al., 2018). To optimize the bnAb maturation shepherding process, there is a need to improve prime-boost approaches.

Here, using a low VRC01-class B cell precursor frequency mouse model using transgenic (Tg) germline-reverted VRC01 B cells (Abbott et al., 2018), we found that a single bolus delivery of a CD4bs GT trimer (Briney et al., 2016) could elicit numerous bnAb class mutations in GC B cells (B_{GC}s). We also explored the effect of increasing the quantity of antigen-specific CD4 T cells on B cell clonality and SHM in late GCs. Importantly, we demonstrate that VRC01-class B cells in GT trimer-immunized mice readily acquire SHMs that are important for Env trimer binding. Recombinantly expressed VRC01-class monoclonal antibodies (mAbs) could neutralize several tier 2 N276 glycan-deficient pseudoviruses, highlighting that an optimized selection of GT immunogens in a sequential immunization schedule, such as early introduction of Env trimer-based booster immunogens, may be key to rapidly and efficiently drive bnAb-type mutations.

RESULTS

Affinity of GT Env trimer affects rare B cell recruitment to GCs

Studies of viral glycoprotein immunizations are needed to better understand competition between neutralizing and off-target B cell responses. Soluble Env trimer immunogens tend to elicit non-neutralizing or autologous neutralizing antibodies that typically target strain-specific glycan holes or the base of the trimer (Cirelli et al., 2019; Klasse et al., 2018; McCoy et al., 2016; Ringe et al., 2019; Zhao et al., 2020). In mice, the base of the trimer is particularly immunodominant (Hu et al., 2015). Additionally, compared with minimalistic CD4bs GT immunogens like eOD (engineered gp120 outer domain)-GT5 (Jardine et al., 2013), the CD4bs on the native Env trimer is sterically occluded by adjacent protomers and surrounding glycans. For example, many VRC01-class bnAbs utilize their HC framework region (FWR) 3 to make contacts with the adjuvant gp120 protomer (Lyumkis et al., 2013). Finally, high-avidity 60-mer immunogens significantly enhance the magnitude of VRC01-class responses (Abbott et al., 2018). These characteristics likely make the CD4bs relatively weakly immunogenic when presented as a soluble Env trimer.

To investigate humoral responses to CD4bs-directed GT immunogens, we utilized inferred-germline VRC01 (glVRC01) B cell receptor (BCR) knockin B cells (VRC01^{gHL}) that express the germline human IGHV1-2*02 HC paired with an IGKV3-11*01 LC (Abbott et al., 2018). The HC and LC have the mature CDR3 of VRC01, except for one mutation to remove an unpaired Cys in the H-CDR3 (Jardine et al., 2015). By adoptively transferring VRC01^{gHL} B cells so that recipient mice have a VRC01^{gHL} B cell precursor frequency of 1 in 10^6 splenic B cells, we can specifically study VRC01-class responses to GT immunogens in a physiological mouse model (Abbott et al., 2018). We questioned whether CD4bs GT trimers would be able to generate VRC01-class responses in a controlled low B cell precursor frequency VRC01^{gHL} model system. To this end, we designed the BG505 MD39-GT3.1 trimer (hereafter abbreviated as MD39-GT3.1). MD39-GT3.1 is a BG505 SOSIP trimer with mutations that improve the antigenic profile, thermal stability, and expression yield (MD39) (Steichen et al., 2016) and contains CD4bs GT mutations described previously in BG505 SOSIP-GT3 (Briney et al., 2016; Figure S1A). MD39-GT3.1 expressed as well-formed trimers, indicated by high thermostability and antigenic binding by trimer-specific antibodies such as PGT151 (Blattner et al., 2014; Falkowska et al., 2014), PGT145 (Walker et al., 2011), and PGDM1400 (Sok et al., 2014) (Figures S1B–S1D). The monovalent dissociation constant (K_D) of glVRC01 antibody fragment (Fab) for MD39-GT3.1 is ~86 nM (Figure S1E), similar to its K_D for eOD-GT5 (K_D = 250 ± 200 nM) used in our previous model system (Abbott et al., 2018; Kato et al., 2020).

To investigate the ability of CD4bs GT Env trimers to activate and recruit VRC01^{gHL} cells, we transferred 10^3 congenically marked VRC01^{gHL} B cells into C57BL/6J mice and immunized the recipient mice with MD39-GT3.1 or BG505 SOSIPv4.1 GT1

CellPress
OPEN ACCESS

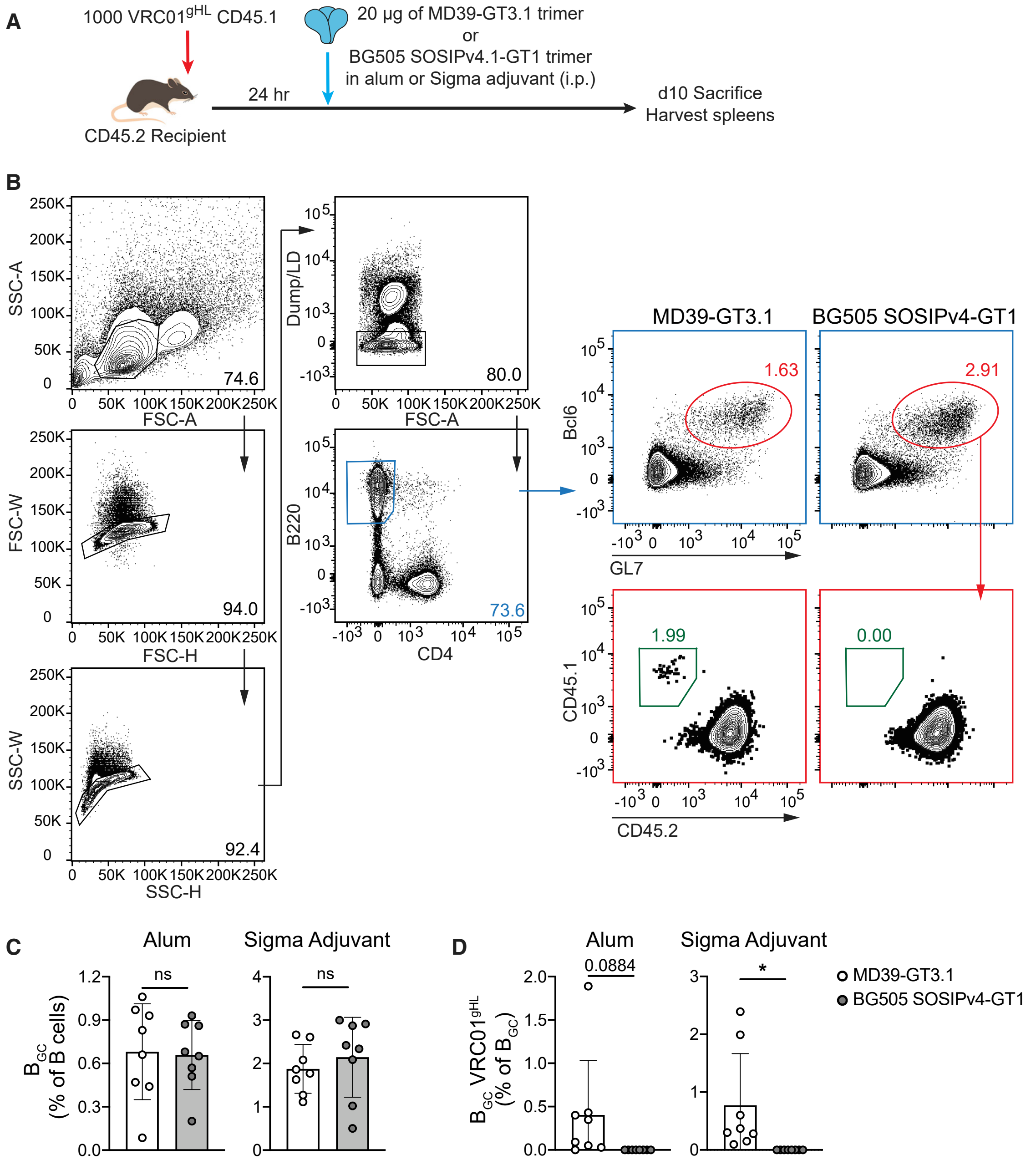

Figure 1. Comparison of two CD4bs GT Env trimer immunogens

(A) Schematic of the experiment.

(B) Representative flow plot showing gating of VRC01^{gHL} B_{GC} cells; from mice immunized with the Sigma adjuvant. Dump: CD8a, NK1.1, Gr-1, live/dead (LD).

(C) Frequency of total B_{GC} cells.

(D) Frequency of VRC01^{gHL} B cells among B_{GC} cells.

N = 2, n = 4, where N is the number of independent experiments, and n is the number of mice per group in each experiment. Mean and standard deviation are shown. Two-tailed Student's t test; not significant (ns), $p > 0.05$; *$p < 0.05$.

(Figure 1A). BG505 SOSIPv4.1 GT1 is a GT trimer designed to engage glVRC01-class bnAbs as well as a trimer apex directed bnAb glPG9 (Medina-Ramírez et al., 2017) (glVRC01 K_D = 1.4 μM; Figures S1A and S1F). A derivative of the BG505 SOSIPv4.1 GT1 Env trimer is currently in a phase 1 clinical trial (ClinicalTrials.gov: NCT04224701). Analysis of spleens on day 10 after immunization revealed that only the higher-affinity MD39-GT3.1 immunogen was able to recruit VRC01^{gHL} cells (Figures 1B–1D), even though MD39-GT3.1 and BG505 SOSIPv4.1 GT1 elicited comparable total B_{GC} responses. The total B_{GC} response to both trimers was increased by ~3-fold when Sigma adjuvant was used instead of alhydrogel alum, but mice immunized with

BG505 SOSIP4v.1 GT1 still failed to generate VRC01^{gHL} B_{GC} cells (Figures 1C and 1D). Thus, monomeric affinity of naive B cells for antigen is a key determinant of B cell activation and GC recruitment by Env trimer immunogens.

Augmentation of CD4 T cell help has a modest effect on late GCs

Selection of high-affinity B cells in the GC involves an interplay between antigen-specific B_{GC} cells and T follicular helper (T_{FH}) cells, where the B_{GC} cells require help from GC T_{FH} cells to undergo rounds of proliferation and affinity maturation. Previously, we explored the effect of increasing T cell help on rare VRC01^{gHL} cells by adoptive transfer of Tg Env-specific T cell receptor (TCR)-expressing CD4 T cells from two different I-A^{b}-restricted Tg TCR mice. CD4 T cells in the HYCAP1 and HYCAP3 mouse lines express a TCR recognizing a 15-mer sequence, PKVSFEPIPIHYCAP, in C2 of Env (Lee et al., 2021a). Co-transferring a moderate number (25 × 10^3 cells) of HYCAP1 or HYCAP3 cells enhanced early proliferation of VRC01^{gHL} cells and increased the proportion of VRC01^{gHL} cells in GCs on day 10 after immunization with MD39-GT3.1 (Lee et al., 2021a).

We investigated whether augmenting CD4 T cell help would continue to influence factors such as late B_{GC} kinetics, ongoing competition between VRC01^{gHL} and polyclonal endogenous B_{GC} cells, and SHM of VRC01^{gHL} B cells. To address these questions, congenically marked 10^3 VRC01^{gHL} B cells were adoptively transferred with or without 25 × 10^3 HYCAP1 or HYCAP3 CD90.1$^+$ CD4 T cells. Recipient mice were immunized with the MD39-GT3.1 trimer in alum (Figure 2A). Total B_{GC} frequencies were comparable irrespective of whether mice received Env-specific CD4 T cells (Figures 2B and 2D). VRC01^{gHL} B_{GC} cell frequencies were increased by ~6-fold on day 10 in the presence of HYCAP CD4$^+$ T cells (Figure 2E), consistent with our previous observations (Lee et al., 2021a). On day 14, the number and frequency of VRC01^{gHL} B_{GC} cells were similar between HYCAP recipients and non-recipients. On day 36, VRC01^{gHL} B_{GC} cells were more abundant under Env-specific CD4 T cell co-transfer conditions (day 36: control versus HYCAP1, $p = 0.0057$; control versus HYCAP3, $p = 0.0065$; Figures 2E and 2F).

Env-specific CD4 T cell responses were quantified by a TCR-dependent activation-induced marker (AIM) assay (Lee et al., 2021a), where antigen-specific CD4 T cells were detected by upregulation of CD40L and CD69 following *ex vivo* restimulation with epitope peptides. The MD39-GT3.1-specific CD4 T cell response remained elevated by ~2- to 4-fold through day 21 among HYCAP CD4 T cell recipients (Figures 2C and 2G). The frequency of AIM$^+$ T_{FH} cells was elevated in HYCAP recipient mice on day 10 (Lee et al., 2021a) but declined to levels comparable with the control group by day 14 (Figures 2C and 2H). Approximately half of the Env-specific T_{FH} cell response was from the transferred HYCAP cells between day 7 and day 10 but declined thereafter (Figure 2I).

Next, we analyzed serum antibody responses. Three different ELISAs were used to distinguish distinct classes of Env-binding responses. When C-terminally biotinylated MD39-GT3.1 trimers captured onto streptavidin (SA)-coated plates, MD39-GT3.1-binding serum immunoglobulin G (IgG) titers increased over time (Figure S2A). Mice that received HYCAP cells had increased MD39-GT3.1-binding serum IgG titers on day 10 and day 14 compared with controls (Figures 2E, 2F, and S2A), consistent with the increased VRC01^{gHL} B_{GC} cells observed on day 10. To determine the proportion of CD4bs-specific IgG, we assessed serum binding to SA-captured biotinylated MD39-GT3.1 knockout (KO) trimers (MD39-GT3.1 trimer with mutations that prevent CD4bs-specific antibody binding). Serum IgG binding to MD39-GT3.1 KO trimers was not detected (Figure S2B), indicating that nearly all SA-captured MD39-GT3.1 trimer-binding IgG was CD4bs specific.

We considered that the lack of serum binding to non-CD4bs epitopes was likely due to the immunodominant Env trimer base (Hu et al., 2015) and that biotinylated C terminus capture of Env trimers may mask and sterically block accessibility to the base (Figure S2C). To test this, we captured BG505 SOSIP Env trimers on plates coated with *Galanthus nivalis* lectin (GNL) (Figure S2C). In GNL ELISA, Env-binding IgGs were observed in all groups. Overall, Env-binding serum titers did not significantly differ between groups, but a trend toward increased titers in HYCAP recipient mice was observed on day 10 (Figures S2A and S2D). We also considered the possibility that endogenous serum responses targeted neoepitopes created upon *in vivo* dissociation of the gp subunits but did not detect serum IgG binding to monomeric BG505 gp120 (Figure S2E). This suggested that the MD39-GT3.1 trimer was stable *in vivo* and that polyclonal Env-binding endogenous responses were almost exclusively to the base. We confirmed the Env trimer base-directed immunodominance by blocking access to this epitope via pre-binding of the base-directed, rhesus macaque (RM)-derived Fab RM19R (Cottrell et al., 2020). RM19R competition drastically reduced Env binding (Figures 2J and S2F). All mice developed VRC01^{gHL}-derived, CD4bs-specific IgG along with trimer base-binding endogenous polyclonal responses. Increasing T cell help had only a modest effect on the serum IgG profile.

To explore whether augmenting CD4 T cell help altered the repertoire of the Env-specific B cell response, BCR sequencing of endogenous B_{GC} cells was performed on days 10, 14, and 21 (Figures 2A and S3A). Clonotype analysis of paired HC-LC BCR sequences showed massive diversity among B_{GC} cells irrespective of CD4 T cell adoptive transfer, suggesting that the trimer base directed response is likely accomplished by diverse murine B cell clonotypes (Figure S3B). The top 5 most frequently observed IGHV and IGKV genes largely overlapped between the control group and those under HYCAP CD4 T cell transfer conditions, many of which were V genes commonly used (>1% representation in the unimmunized repertoire; Rettig et al., 2018) within the mouse repertoire (Figures S3C and S3D). Thus, increasing CD4 T cell help enhanced early proliferation of rare, high-affinity VRC01^{gHL} B_{GC} cells but had no detectable effect on late GC kinetics and polyclonal endogenous Env-binding B cell responses, possibly because of equivalence in Env-specific T cell frequencies at later time points.

One Env trimer immunization can promote extensive VRC01-class SHMs

GC-derived VRC01^{gHL} BCRs were sequenced on days 10, 14, 21, and 36 after MD39-GT3.1 Env trimer immunization (Figures

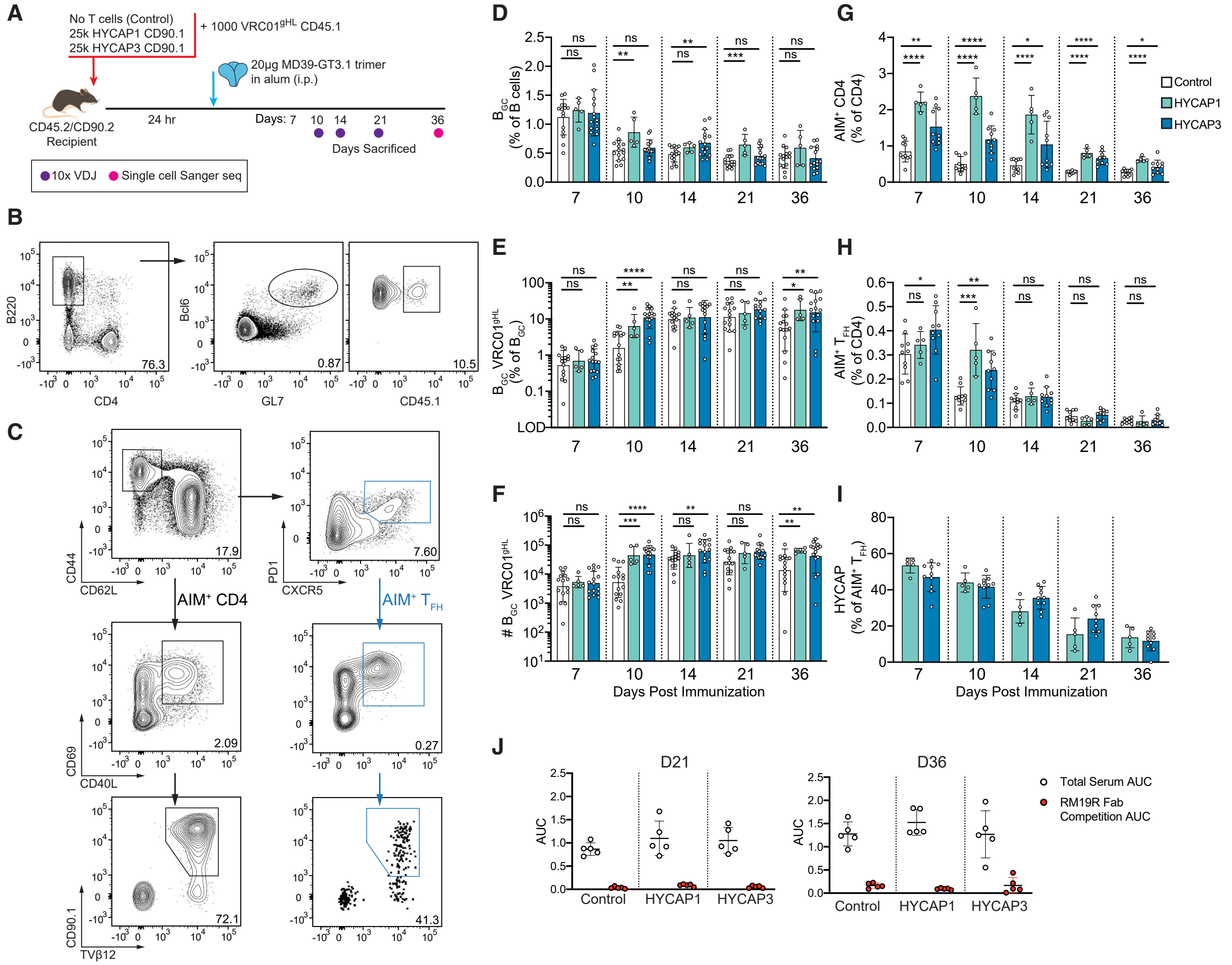

Figure 2. VRC01-class B_{GC} cell kinetics in response to MD39-GT3.1

(A) Schematic of the experiment. Day 21 corresponds to either day 21 or day 22, depending on the replicate. Control: N = 3, n = 5; HYCAP1: N = 1, n = 5; HYCAP3: N = 3, n = 5.

(B) Representative flow plot showing B_{GC} VRC01^{gHL} gating on day 10, from a HYCAP3 T cell recipient mouse. Cells were gated as in Figure 1C.

(C) Representative flow plot showing the AIM gating strategy from a HYCAP1 T cell recipient mouse on day 10. Cells are gated on CD4$^+$ T cells. Frequencies shown in the AIM$^+$ (CD40L$^+$CD69$^+$) gates shows the percentage of AIM$^+$ cells among total CD4 T cells. Control: N = 2, n = 5; HYCAP1: N = 1, n = 5; HYCAP3: N = 2, n = 5.

(D) Frequency of total B_{GC} cells.

(E) Percentage of VRC01^{gHL} cells among B_{GC} cells; gated as CD45.1$^+$ or CD45.1$^-$CD45.2$^+$, depending on the congenic marker of transferred VRC01^{gHL} cells.

(F) Number of B_{GC} VRC01^{gHL} cells in the spleen, back-calculated using the number of total splenocytes.

(G) Total MD39-specific CD4 T cell responses, gated as in (C).

(H) MD39-specific T_{FH} responses, gated as in (C).

(I) Percentage of HYCAP (CD90.1$^+$TVβ12$^+$) cells among total MD39-specific T_{FH} cells.

(J) Area under the curve (AUC) analysis of total serum response to the trimer with and without RM19R competition.

Pooled data from 3 experiments are shown in (D–F). Pooled data from 2 experiments are shown in (G–I). Mean and standard deviation are shown for graphs on linear scales. Geometric mean and geometric standard deviation are shown for data graphed in log scale. Two-tailed Student's t test: ns, $p > 0.05$; *$p < 0.05$; **$p < 0.01$; ***$p < 0.001$; ****$p < 0.0001$.

2A, S3A, and S4A). VRC01^{gHL} B_{GC} cells acquired numerous mutations after a single immunization (Figures 3A and 3B). Even though co-transfer of Env-specific CD4 T cells increased the number of VRC01^{gHL} B_{GC} cells on day 10, the rates and types of SHM acquired by VRC01^{gHL} B cells were unaffected over time (Figure S4B).

Because SHMs in all three experimental groups were similar (Figures S4C and S4D), VRC01^{gHL} BCR sequence data from all groups were combined for further analysis. By day 36, the median number of amino acid mutations was 6 in the HC and 3 in the LC. Most mutations occurred in the V gene because VRC01^{gHL} BCR possessed mature CDR3 sequences. HC

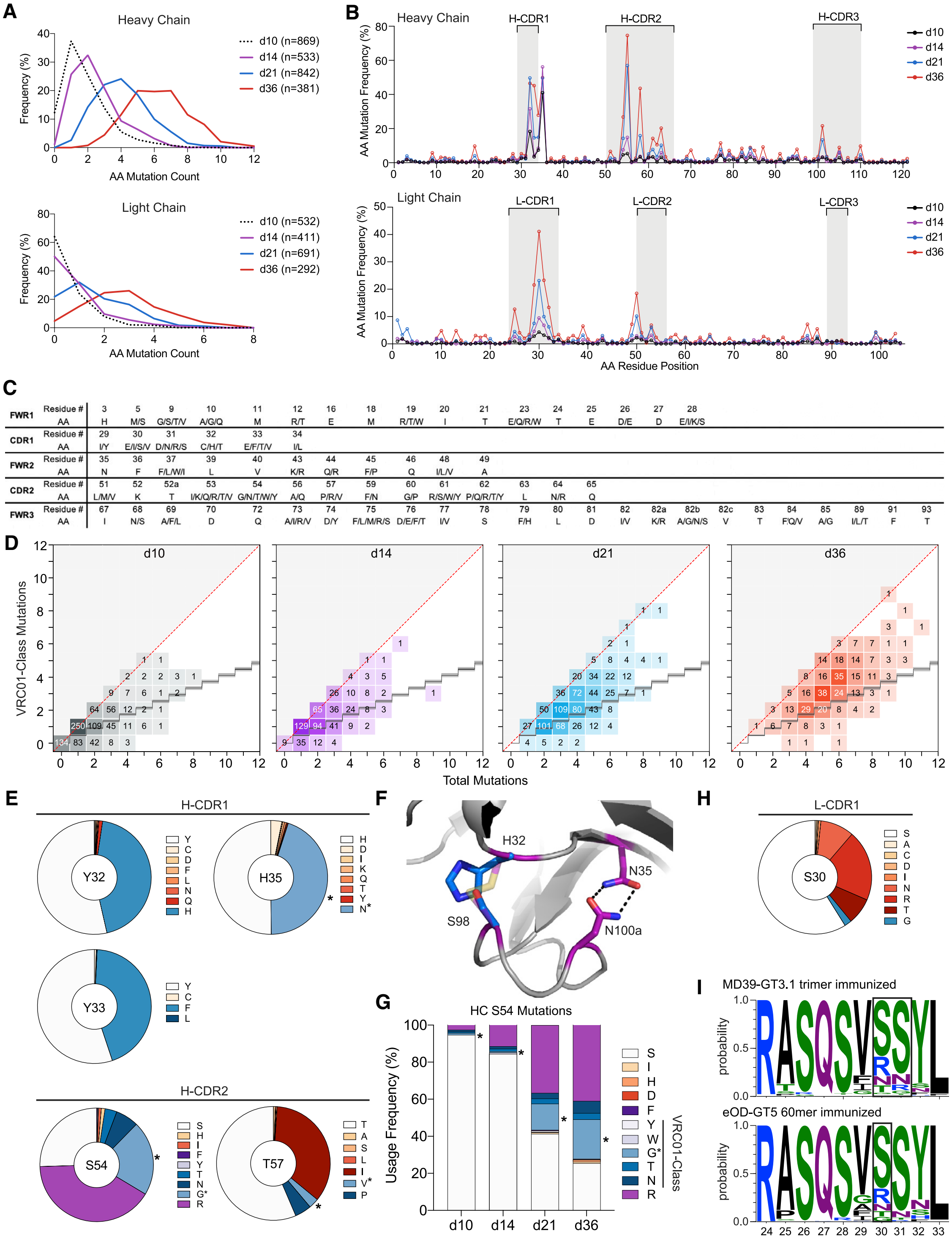

FWR1	Residue #	3	5	9	10	11	12	16	18	19	20	21	23	24	25	26	27	28							
	AA	H	M/S	G/S/T/V	A/G/Q	M	R/T	E	M	R/T/W	I	T	E/Q/R/W	T	E	D/E	D	E/I/K/S							
CDR1	Residue #	29	30	31	32	33	34																		
	AA	I/Y	E/I/S/V	D/N/R/S	C/H/T	E/F/T/V	I/L																		
FWR2	Residue #	35	36	37	39	40	43	44	45	46	48	49													
	AA	N	F	F/L/W/I	L	V	K/R	Q/R	F/P	Q	I/L/V	A													
CDR2	Residue #	51	52	52a	53	54	56	57	59	60	61	62	63	64	65										
	AA	L/M/V	K	T	I/K/Q/R/T/V	G/N/T/W/Y	A/Q	P/R/V	F/N	G/P	R/S/W/Y	P/Q/R/T/Y	L	N/R	Q										
FWR3	Residue #	67	68	69	70	72	73	74	75	76	77	78	79	80	81	82	82a	82b	82c	83	84	85	89	91	93
	AA	I	N/S	A/F/L	D	Q	A/I/R/V	D/Y	F/L/M/R/S	D/E/F/T	I/V	S	F/H	L	D	I/V	K/R	A/G/N/S	V	T	F/Q/V	A/G	I/L/T	F	T

Figure 3. VRC01^{gHL} mAbs acquire bnAb-like VRC01-class mutations after MD39-GT3.1 immunization

(A) Accumulation of HC and LC amino acid mutations in VRC01^{gHL} B_{GC} cells. n is the total number of sequences analyzed. Detailed sampling information is presented in Figure S4.

(B) Day 36 per-residue mutation frequency of HC and LC amino acid residues. Residue positions are numbered linearly.

(C) List of IGHV1-2-region VRC01-class mutations.

(D) Total and VRC01-class amino acid mutations in the IGHV1-2 region. The diagonal red line represents maximum VRC01-class mutations. The staircase shows the background level of random mutations.

(legend continued on next page)

mutations predominantly accumulated in H-CDR1 and H-CDR2 (Figures 3B, S4C, and S4D), similar to what was observed for longitudinal development of a VRC01-class bnAb, PCIN63 (Umotoy et al., 2019). Some SHMs were key mutations identified in the engineered, minimally mutated bnAb MinVRC01 (Jardine et al., 2016b). The number of VRC01-class HC mutations, defined as IGHV1-2 mutations observed in a reference panel of VRC01-class bnAbs (Briney et al., 2016), increased substantially over time (Figures 3C and 3D).

The most common SHMs were examined in greater detail. One highly selected mutation was H35N, which drives the H-CDR1$_{N35}$:H-CDR3$_{N100a}$ (Kabat numbering) CDR loop-stabilizing interaction in VRC01 (~40%–50% of all day 36 HC sequences; Figure 3E), which has been observed in several other immmunization models (Abbott et al., 2018; Briney et al., 2016; Jardine et al., 2015). Some HCs acquired a Y32H mutation instead, which, like H35N, would form a CDR loop-stabilizing interaction by hydrogen bonding with H-CDR3$_{S98}$ (Figures 3E and 3F). Mature VRC01 acquires stabilizing mutations at both paired positions, but HC residues C32 and C98 form a disulfide bond instead (Zhou et al., 2010). The proportion of HCs that acquired either H35N or Y32H equated to ~88%–90% of all HC sequences, highlighting the importance of this loop-stabilizing interaction in VRC01 affinity maturation.

On day 36, ~45% of the sequenced HCs were mutated at position 33, most of which acquired a Y33F mutation, except for one clone with Y33L and two clones with Y33C mutations (Figure 3E). Mutation of Y33 in H-CDR1 to a smaller hydrophobic residue has been demonstrated previously to enhance neutralization potency (Chen et al., 2021). A phenylalanine substitution improved neutralization IC_{50} to wild-type viruses, but less potently than smaller hydrophobic residues, such as valine (Chen et al., 2021).

The single most frequent HC mutation occurred at position S54 in the H-CDR2 (~70%–80% of day 36 HC sequences). Approximately 21% of the analyzed sequences acquired a glycine in this position, which is a mutation in mature VRC01. An arginine mutation was seen in an additional ~41% of day 36 sequences (Figure 3E), which accumulated over time (Figure 3G). Although arginine and phenylalanine are not considered VRC01-class mutations based on our reference set of antibodies, residue 54 in the HC of several other VRC01-class antibodies has either an aromatic side chain or arginine, which fills the highly conserved CD4 F43 cavity formed between the bridging sheet and CD4-binding loop of gp120 (Zhou et al., 2015). This VRC01-class mutation was also observed in mice boosted with the BG505 N276A trimer (Briney et al., 2016) or mice immunized with other CD4bs GT immunogens, such as 426c core or 426c core variants, which retain more gp120 features than eOD (Parks et al., 2019; Tian et al., 2016). In contrast, S54 was much less mutated in eOD-GT5- or eOD-GT8 60-mer-immunized mice (Abbott et al., 2018; Chen et al., 2021; Huang et al., 2020), likely because eOD-GT constructs lack the equivalent of the gp120 bridging sheet and hence, do not contain a complete F43 pocket.

T57 in H-CDR2 was also heavily mutated, with ~35% of the mutated residues being an isoleucine and another ~8% being a T57V/P VRC01-class mutation (Figure 3E). T57V is a commonly found mutation among VRC01-class bnAbs and is also one of the mutations required for neutralization breadth in MinVRC01 and Min12A21 (Jardine et al., 2016b). Because of the similar chemical characteristics of isoleucine and valine, the T57I mutation could be a potential alternative to T57V.

LC SHMs were focused in L-CDR1 (Figure 3B), particularly in the region surrounding S30. Residues like serine and glycine, which confer loop flexibility, were observed (Figure 3H). The S30R mutation was also common and, along with a few S31R mutations, was likely selected because of the L-CDR1-proximal D276 residue in gp120, resulting from knocking out the N276 glycan (Figures 3H and 3I). Selection of arginine residues in L-CDR1 has been observed following immunization with eOD-GT immunogens with an aspartic acid residue in the N276-equivalent position of eOD-GT5 (Abbott et al., 2018; Huang et al., 2020). These results highlight that VRC01-class B_{GC} cells accumulate a considerable number of epitope-specific mutations after a single GT trimer immunization.

MD39-GT3.1-immunized mice acquire improbable mutations, including L-CDR1 deletions

4 clones with L-CDR1 deletions were found among MD39-GT3.1 trimer-immunized mice (two clones isolated each on day 21 and day 36), representing a substantial L-CDR1 deletion rate of 0.38% on day 21 and 1.12% on day 36 (among HYCAP1 and HYCAP3 recipient mice; Figures 4A and S4A). L-CDR1 deletions in VRC01-class bnAbs accommodate the N276 glycan and are considered a critical hurdle for acquisition of breadth (Zhou et al., 2013). One additional clone with an L-CDR1 deletion was obtained from day 36 VRC01^{gHL} B_{GC} cells of eOD-GT5 60-mer-immunized mice (Abbott et al., 2018). The deletions occurred around mutation hotspot motifs in IGKV3-11, defined by the antigen receptor mutation analyzer for detection of low-likelihood occurrences (ARMADiLLO) software (Figure 4B; described further below). Deletions in L-CDR1 after a single immunization were also observed in another adoptive transfer mouse model (HuGL18) using Tg B cells expressing an authentic VRC01-class precursor BCR isolated from naive human B cells (Huang et al., 2020). HuGL18 B cells use an IGKV3-20 gene, which is similar to IGKV3-11. Certain human IGKV genes may be more poised toward acquiring deletions in the L-CDR1. Interestingly, when using a human germline IGHV1-2 and IGKV3-20

(E) Most frequently mutated HC residues on day 36. Shades of blue indicate VRC01-class mutations. Asterisks indicate VRC01 matched mutations. Red shades indicate SHMs not observed in our reference VRC01-class antibodies. Residues are shown in Kabat numbering.
(F) VRC01 HC CDR loop conformations are stabilized via H-CDR1$_{N35}$-H-CDR3$_{N100a}$ and H-CDR1$_{C32}$-H-CDR3$_{C98}$ interactions (purple sticks). VRC01^{gHL} cells from MD39-GT3.1-immunized mice achieve the stabilizing interaction via H-CDR1$_{H32}$-H-CDR3$_{S98}$ (blue sticks) or H-CDR1$_{N35}$-H-CDR3$_{N100a}$ (PDB: 4JPI).
(G) Mutations observed in HC position S54.
(H) Day 36 LC mutations observed in S30. Color coding is as in (D).
(I) Selection of arginine residues in L-CDR1 positions 30 and 31 on day 36 after immunization with the MD39-GT3.1 trimer or eOD-GT5 60-mer.

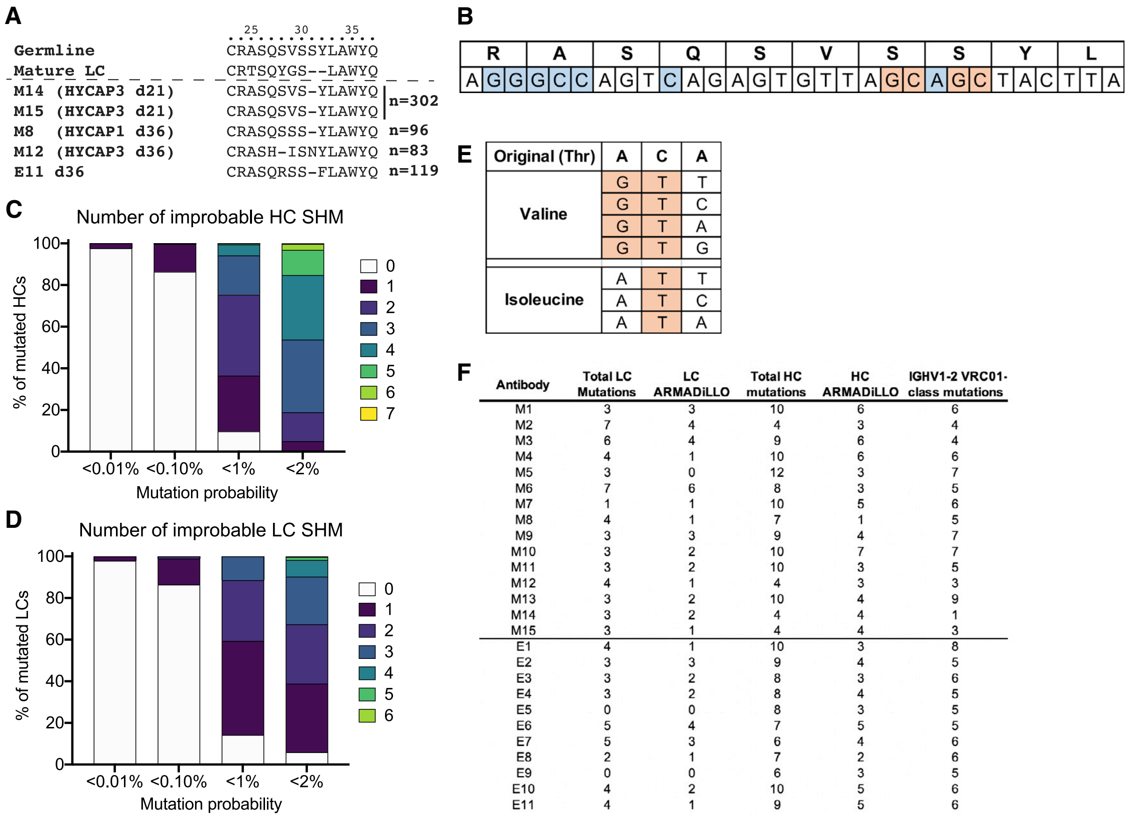

Antibody	Total LC Mutations	LC ARMADiLLO	Total HC mutations	HC ARMADiLLO	IGHV1-2 VRC01-class mutations
M1	3	3	10	6	6
M2	7	4	4	3	4
M3	6	4	9	6	4
M4	4	1	10	6	6
M5	3	0	12	3	7
M6	7	6	8	3	5
M7	1	1	10	5	6
M8	4	1	7	1	5
M9	3	3	9	4	7
M10	3	2	10	7	7
M11	3	2	10	3	5
M12	4	1	4	3	3
M13	3	2	10	4	9
M14	3	2	4	4	1
M15	3	1	4	4	3
E1	4	1	10	3	8
E2	3	3	9	4	5
E3	3	2	8	3	6
E4	3	2	8	4	5
E5	0	0	8	3	5
E6	5	4	7	5	5
E7	5	3	6	4	6
E8	2	1	7	2	6
E9	0	0	6	3	5
E10	4	2	10	5	6
E11	4	1	9	5	6

Figure 4. VRC01^{gHL} B$_{GC}$ cells acquire rare mutations, including L-CDR1 deletions

(A) Clones with an L-CDR1 deletion after immunization with the MD39-GT3.1 trimer (M8, M12, M14, M15) or eOD-GT5 60-mer (E11). n is the total number of sequences analyzed. The three day 36 clones are derived from different mice. M14 and M15 may or may not be from the same mouse.

(B) Mutation cold spots (blue) and hotspots (orange) in L-CDR1 of IGKV3-11.

(C and D) ARMADiLLO analysis of mutated day 36 HC (C) and LC (D) sequences from MD39-GT3.1-immunized mice, showing the fraction of sequences that acquired the given number of improbable mutations.

(E) Codon changes (orange) required for a threonine-to-valine/isoleucine mutation.

(F) Antibodies selected for expression and their mutation profiles.

knockin mouse model (BCRs express IGHV1-2 HC rearranged with various mouse-derived H-CDR3s and IGKV3-20 LC with mature VRC01 L-CDR3), LC deletions were observed after at least 6 boosts with sequential shepherding (Chen et al., 2021).

It has been suggested that a roadblock for developing breadth via immunization is due to critical mutations being "improbable" mutations that occur outside of mutation hotspots (Saunders et al., 2019). Thus, we analyzed the IGHV1-2 and IGKV3-11 regions to determine whether a single priming immunization was able to elicit improbable mutations in VRC01^{gHL} B cells using the ARMADiLLO software, which defines improbable mutations as those that have a less than 2% probability of occurring in the absence of selection pressure (Saunders et al., 2019; Wiehe et al., 2018). All HC sequences and most LC sequences had at least one ARMADiLLO-defined improbable mutation by day 36 after a single MD39-GT3.1 immunization. Many of the HCs contained 5 or more such improbable mutations (<2% mutation probability cutoff; Figures 4C and 4D). Notably, key H-CDR2 mutations, such as S54G (mutation probability, 1.0%–0.1%), S54R (mutation probability, 2.0%–1.0%), and T57I (mutation probability, 1.0%–0.1%), were deemed improbable by ARMADiLLO. The preferential selection of isoleucine at T57 is likely due to the valine mutation requiring a minimum of two nucleotide changes (Figure 4E). Multiple boosts are not necessarily required to achieve ARMADiLLO-defined improbable mutations.

Affinity-matured binding to N276 glycan-deficient, wild-type Env trimers

Having confirmed that VRC01-class SHMs occurred in response to MD39-GT3.1, we proceeded to assess affinity maturation. We recombinantly expressed day 36 clones with an L-CDR1 deletion along with several paired BCR sequences that had some of the highest number of VRC01-class IGHV1-2 mutations (Table S1; Figure 4F). 4–5 paired BCR sequences were selected from each group, totaling 13 mAbs (M1–M13; L-CDR1 deletions in M8 and M12). Using the same criteria, an additional 11 mAbs from day 36 B$_{GC}$ VRC01^{gHL} BCRs from our previous eOD-GT5 60-mer immunization study (Abbott et al., 2018) were also expressed (E1–E11; L-CDR1 deletion in E11; Table S1; Figure 4F). MD39-GT3.1-elicited mAbs had K_D values of 6.4–0.92 nM for the

CellPress
OPEN ACCESS

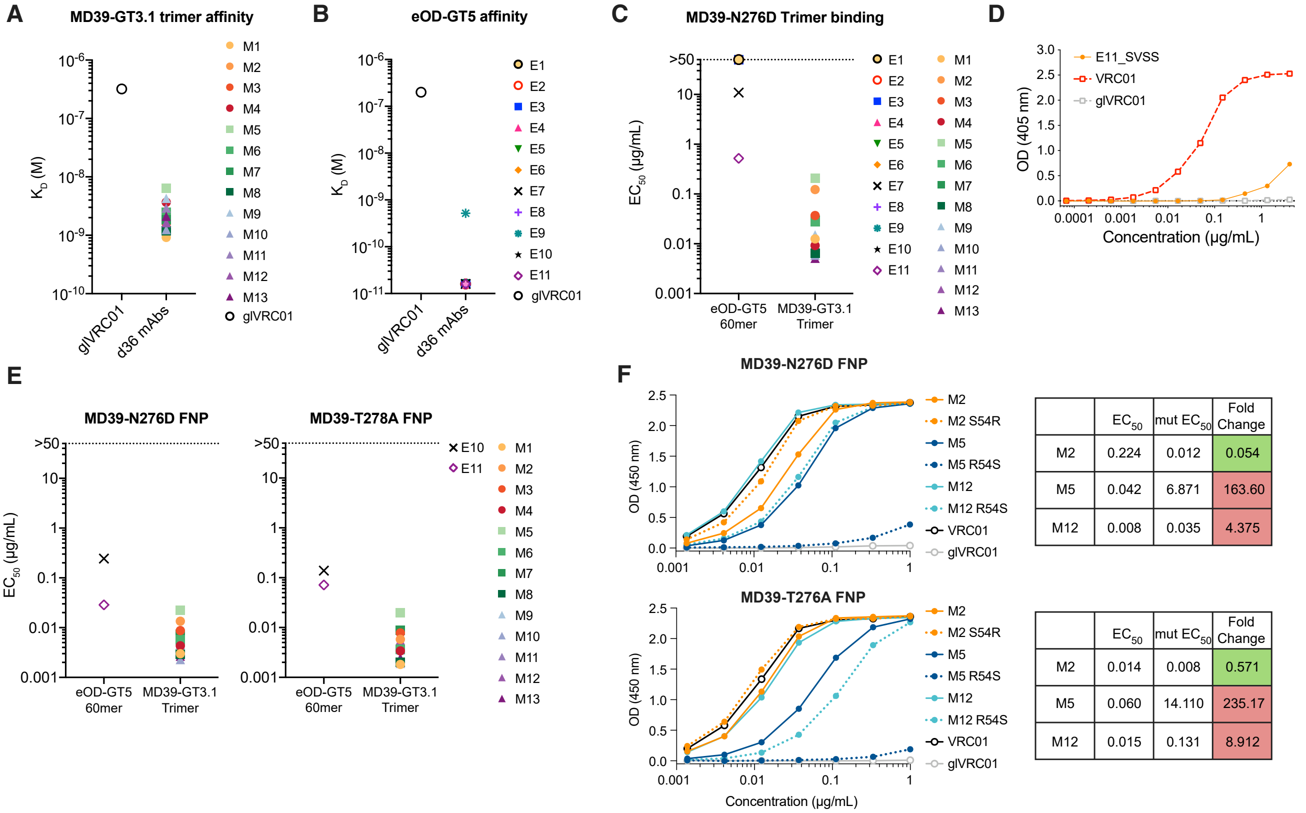

Figure 5. VRC01^{gHL} mutations from a single bolus immunization confers substantial affinity

(A) Monovalent K_D of Fabs from day 36 after immunization with the MD39-GT3.1 trimer, measured by BLI Octet. Data are an average of two experiments.
(B) Monovalent K_D of Fabs from day 36 after immunization with the eOD-GT5 60-mer, measured by SPR.
(C) mAb binding ELISA EC_{50} to MD39-N276D trimers captured on SA-coated plates.
(D) E11 L-CDR1 deletion reversion mutants binding to MD39-N276D trimers captured on SA-coated ELISA plates.
(E) mAb binding to MD39-N276D trimer ferritin nanoparticles (FNPs) and MD39-T278A trimer FNPs on directly coated ELISA plates.
(F) Binding of mAbs with the HC_{S54R} mutation or HC_{R54S} reversion to MD39-N276D (top) or MD39-T278A (bottom) trimer FNPs, measured by ELISA, and change in EC_{50} relative to the parent mAb following addition (M2) or reversion (M5 and M12) of the HC_{R54} mutation.

MD39-GT3.1 trimer, measured by biolayer interferometry (BLI), averaging ~130-fold gain in affinity (Figure 5A). 10 of 11 eOD-GT5 60-mer immunization-derived mAbs had a K_D of ~16 pM for the eOD-GT5 monomer, which is the highest affinity quantifiable by Bio-Rad ProteOn surface plasmon resonance (SPR) (Figure 5B). This was an exceptional, ~12,500-fold improvement in affinity relative to the unmutated precursor, glVRC01. These results demonstrate that immunization with an Env trimer-based (MD39-GT3.1) or minimalistic (eOD-GT5 60mer) GT immunogen can induce high-affinity SHMs in VRC01^{gHL} B cells after a single immunization.

Because all mAbs gained substantial affinity to the autologous GT immunogen, we assessed whether the mutated mAbs could bind a more native CD4bs epitope. We tested binding of each mAb to MD39 trimers lacking the glycan at position N276 (ΔN276). All MD39-GT3.1 immunization-elicited mAbs bound the MD39-N276D trimer by ELISA, compared with only two eOD-GT5 60-mer elicited mAbs (Figure 5C). One of the two mAbs from eOD-GT5 60-mer-immunized mice capable of binding MD39-N276D Env was E11, which had a deletion in L-CDR1 (Figure 5C). E11 binding to MD39-N276D was strongly dependent on the deletion because reverting the deletion abolished binding to the trimer (Figure 5D). All mAbs that bound the MD39-N276D trimer also recognized a second ΔN276 mutant, MD39-T278A (Figure 5E). None of the mAbs were able to bind MD39 with the N276 glycan (data not shown), as expected after only a single immunization.

The HC_{S54R} substitution was present in 5 of 13 MD39-GT3.1-elicited mAbs but none in mAbs from eOD-GT5 60-mer-immunized mice (Abbott et al., 2018). We next tested whether this mutation was important for Env trimer binding. When the S54R mutation was added to mAb M2, its binding to MD39-N276D and MD39-T278A trimers improved (Figure 5F). On the other hand, when R54 was reverted to the germline serine residue in M5 and M12, ΔN276 trimer binding was reduced ~4- to 8-fold for M12 and more than 160-fold for M5 (Figure 5F). This indicated that F43 cavity-recognizing mutations are highly favorable for mediating Env trimer binding and that such mutations arise selectively after exposure to immunogens displaying nearly complete Env CD4bs epitopes.

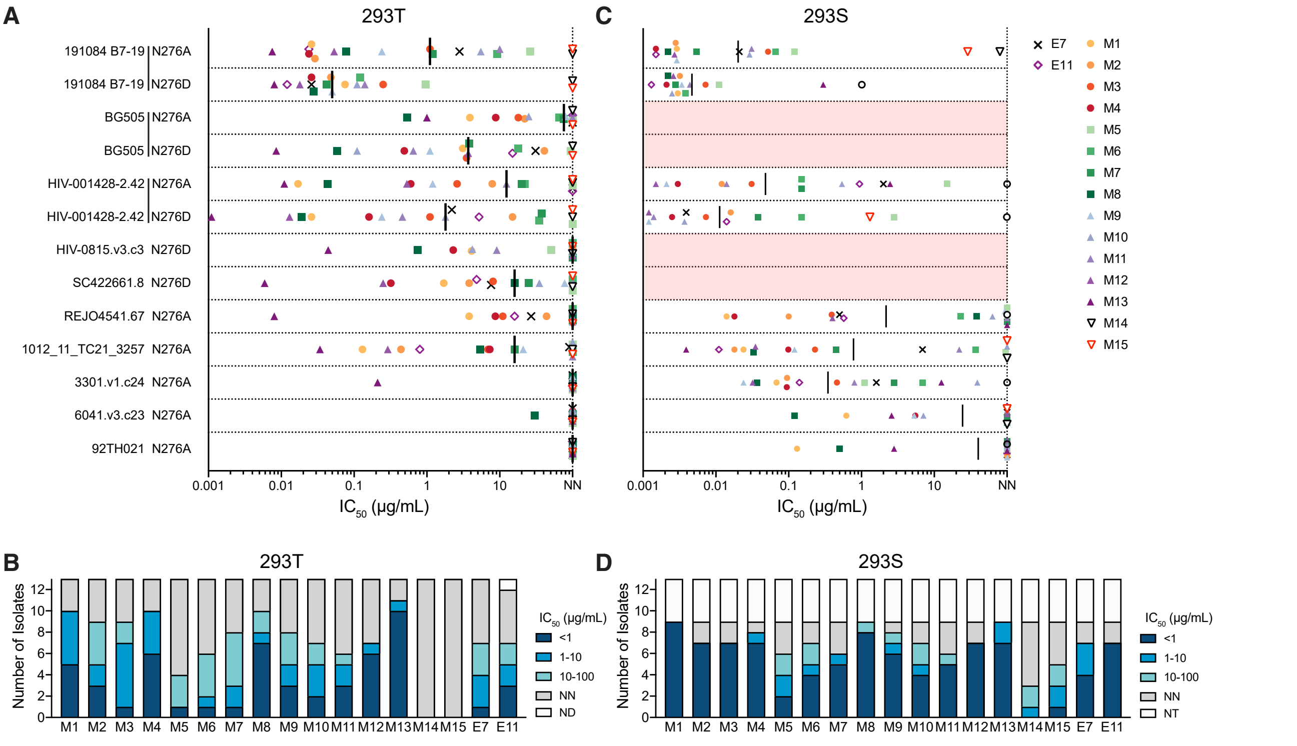

E

	M1	M2	M3	M4	M5	M6	M7	M8	M9	M10	M11	M12	M13	M14	M15	E7	E11	VRC01
191084 B7-19 N276A	1.12E-01	9.66E-02	4.73E-02	6.25E-02	4.62E-03	7.10E-03	4.50E-03	2.82E-02	1.21E-02	5.27E-03	3.10E-03	5.09E-02	6.67E-02	7.97E-01	2.88E-01	7.50E-03	6.25E-02	2.95E-02
191084 B7-19 N276D	4.00E-02	6.67E-02	2.88E-02	8.08E-02	1.15E-02	3.17E-02	6.67E-02	7.86E-02	6.80E-02	2.27E-02	3.14E-02	1.44E-01	3.00E-02	1.01E-02	2.98E-03	2.85E-02	1.08E-01	1.93E-01
HIV-001428-2.42 N276A	4.82E-02	1.52E-03	1.19E-02	5.00E-03	1.50E-01	6.82E-03	7.50E-03	1.33E-02	1.75E-03	5.40E-03	1.13E-03	2.83E-03	1.82E-03	NN	2.47E-02	2.00E-02	9.40E-03	4.36E-01
HIV-001428-2.42 N276D	3.27E-02	1.07E-03	6.64E-03	1.56E-02	2.80E-02	4.29E-03	1.00E-03	1.95E-02	5.00E-03	2.06E-03	3.04E-03	9.23E-02	9.09E-03	9.95E-01	1.31E-02	1.77E-03	2.69E-03	2.30E-01
REJO4541.67_N276A	3.68E-03	2.27E-03	3.55E-02	2.07E-03	NN	2.30E-01	NN	3.80E-01	9.30E-01	6.30E-01	NN	4.00E-03	1.25E-03	NN	NN	1.85E-02	3.56E-02	1.67E-01
1012_11_TC21_3257_N276A	1.85E-01	4.09E-02	3.33E-02	1.36E-02	9.60E-01	3.70E-01	2.81E-02	6.11E-03	5.71E-03	NN	2.20E-01	1.21E-01	1.15E-01	NN	NN	7.67E-02	1.38E-02	1.50E+01
3301.v1.c24_N276A	6.80E-04	9.50E-04	4.60E-03	9.40E-04	1.10E-02	6.90E-02	2.80E-02	3.70E-04	2.40E-04	3.90E-01	8.00E-03	3.20E-04	4.76E-05	NN	1.25E-01	1.60E-02	1.40E-03	3.78E-02
6041.v3.c23_N276A	6.20E-03	NN	NN	5.50E-02	NN	NN	NN	4.00E-03	5.30E-02	7.10E-02	NN	NN	2.60E-02	NN	NN	NN	NN	3.22E-01
92TH021_N276A	1.30E-03	NN	NN	NN	NN	NN	NN	5.00E-03	NN	NN	NN	NN	2.80E-02	NN	NN	NN	NN	1.67E-01

Fold change IC_{50}	Fold Improvement
>0.02	<50
0.01 - 0.02	50 - 100
0.002 - 0.01	100 - 500
0.001 - 0.002	500 - 1000
<0.001	>1000

Figure 6. Mutated VRC01^{gHL} mAbs neutralize heterologous ΔN276 pseudoviruses

(A) Neutralization IC_{50} to ΔN276 pseudoviruses produced in HEK293 T cells. Non-neutralizing (NN): IC_{50} > 100 μg/mL. The black line indicates the median. Numerical IC_{50} values are shown in Figure S5.

(B) Number of viral isolates shown in (A), neutralized by each mAb with the indicated IC_{50} range. ND, not determined.

(C) As in (A) but for pseudoviruses produced in HEK293S cells. Red bars indicate viruses that could not be produced.

(D) As in (B) but for pseudoviruses produced in HEK293S cells. NT, no virus titer.

(E) Fold change in neutralization IC_{50} between HEK293T- and HEK293S-produced viruses. For non-neutralized HEK293T viruses, an IC_{50} value of 100 μg/mL was used to calculate fold change. NA, not calculated because of insufficient data.

Single GT Env trimer immunization elicits neutralizing antibodies against ΔN276 HIV

Encouraged by the Env trimer binding properties of day 36 VRC01^{gHL} mAbs, we investigated whether ΔN276 trimer binding would translate to neutralization of ΔN276 HIV viruses. In an initial screening against a panel of 28 ΔN276 pseudoviruses, all MD39-N276D trimer-binding mAbs weakly neutralized at least one ΔN276 isolate (data not shown). The M13 mAb from the HYCAP3 CD4 T cell transfer group was the most broad and potent. We next assessed the day 36 mAbs along with two day 21 mAbs with an L-CDR1 deletion (M14 and M15; Figures 4A and 4F; Table S1) for neutralizing activity against nine N276A viruses neutralized by M13 in the initial screening, along with four N276D isolates (Figures 6A, 6B, and S5). The viruses screened were from various clades, all of which were tier 2 except one isolate (1012_11_TC21_3257; Figure S5). N276D viruses were neutralized with greater potency than their N276A counterparts (Figure 6A), likely because of D276 in the GT immunogens. Most mAbs were typically weakly neutralizing, with an IC_{50} greater than 1 μg/mL. Nevertheless, several mAbs from all experimental groups (control, M4; HYCAP1, M8; HYCAP3, M13) had high potency, having a median neutralization IC_{50} of less than 1 μg/mL (Figure 6B). The most broad and potent mAb, M13, neutralized 10 of 13 isolates tested (8 of 10 when excluding N276A/N276D variant duplicates) with an average IC_{50} of 0.12 μg/mL. This degree of potency is comparable with that of the best mAbs isolated from VRC01^{gH} mice that were primed with eOD-GT8 60-mer, followed by two successive boosts (core BG505 GT3 60-mer and N276A BG505 trimer; Briney et al., 2016). M13 could neutralize several ΔN276 isolates with an IC_{50} comparable with mature VRC01 (Figure S5).

Immature VRC01-class antibodies have been shown to neutralize pseudoviruses better when glycans surrounding the CD4bs are either missing or are high mannose (Briney et al., 2016; LaBranche et al., 2018; Parks et al., 2019). There are complex glycans in the CD4bs region, such as N462 and N197, that may sterically hinder CD4bs epitope accessibility more so than high-mannose glycans (Cao et al., 2017; Struwe et al., 2018). We produced pseudoviruses in GnT1-deficient HEK293S cells that are unable to process high-mannose glycans into complex glycans, which were neutralized with improved potency and breadth (Figures 6C, 6D, and S5). Many IC_{50} values were enhanced more than 100-fold relative to the equivalent pseudoviruses with complex glycans (Figure 6E). These results were in agreement with a study where $VRC01^{gH}$ mice were immunized with the 426c core; $VRC01^{gH}$ mAbs from immunized mice were able to neutralize high-mannose-expressing autologous ΔN276 virus but not HEK293 T cell-produced ΔN276 viruses expressing native glycoforms (Parks et al., 2019). These results highlight that MD39-GT3.1 trimer-induced SHMs confer significant progress toward breadth. In this model system, breadth to ΔN276 viruses can arise in response to a single priming dose, although boosts would be required to generate mutations that allow the BCR to overcome the steric barrier of the complete Env glycan shield.

DISCUSSION

Generating potent broadly neutralizing responses to the heavily glycosylated HIV Env trimer can be challenging because of the immunodominant proteinaceous base. Here we employed a B cell and T cell adoptive transfer model to explore GC kinetics, B_{GC} cell diversity, and SHM levels of B_{GC} cells in a rare naive B cell precursor model for VRC01-class bnAbs. We observed that a single bolus immunization with the MD39-GT3.1 trimer primed rare $VRC01^{gHL}$ cells and that $VRC01^{gHL}$ B_{GC} cells accumulated high-affinity VRC01 bnAb-type SHMs. Several LC sequences acquired an amino acid deletion in the L-CDR1. $VRC01^{gHL}$ B cells acquiring an L-CDR1 deletion were only observed under conditions of augmented CD4 T cell help. mAbs with detectable binding to the MD39-N276D trimer neutralized several ΔN276 heterologous virus strains.

BnAbs are typically highly mutated because of accumulation of SHMs over several years. However, not all mutations are required for bnAb breadth and potency (Jardine et al., 2016b). It has been proposed that one of the roadblocks for development of bnAbs is the difficulty of inducing selection of "improbable" mutations that are necessary for breadth and potency (Saunders et al., 2019; Wiehe et al., 2018). 30%–40% of HCs in MD39-GT3.1-primed $VRC01^{gHL}$ clones acquired substitutions defined as improbable mutations by ARMADiLLO. Some of these mutations are thought to be important for breadth (Jardine et al., 2016b). The data presented here demonstrate that, under immunization-induced selective pressure, improbable bnAb-class mutations, defined by the less than 2% likelihood cutoff, can occur rapidly and frequently. These results provide the promising outlook that key VRC01-class mutations may not be as difficult to acquire as suggested previously (Wiehe et al., 2018). In our study, the proportion of cells with VRC01-class mutations at key positions (Y33, S54, and T57) after one immunization occurred at nearly equivalent or greater frequencies as those observed after the fourth immunization in a sequential immunization study (Chen et al., 2021).

All recombinantly expressed day 36 mAbs with detectable binding to the MD39-N276D trimer neutralized several ΔN276 heterologous virus strains. The IGHV1-2 HC in $VRC01^{gHL}$ and $VRC01^{gH}$ mice (Briney et al., 2016; Jardine et al., 2015; Parks et al., 2019) includes the mature VRC01 H-CDR3. HC and LC CDR3s contribute directly to VRC01-class affinity, neutralization potency, and breadth. Thus, having predefined CDR3s may have facilitated the quick development of breadth to ΔN276 viruses. Regardless, it is important to note that mAbs derived from MD39-GT3.1 trimer-immunized mice outperformed mAbs from eOD-GT5 60-mer-immunized mice (Abbott et al., 2018) in the same $VRC01^{gHL}$ B cell adoptive transfer model system. The numbers of total and VRC01-class mutations in both studies were comparable, but mAbs from MD39-GT3.1-immunized mice had significantly enhanced binding to the MD39-N276D trimer, whereas most mAbs from eOD-GT5 60-mer-immunized mice failed to bind (Figure 5). An early introduction of trimer GT immunogens may accelerate acquisition of broadly neutralizing SHMs in animals and humans. For example, the BG505-GT3 trimer was originally designed not as a priming immunogen but as a first-boost immunogen to an eOD-GT8 60-mer prime, which has significantly higher affinity for bnAb precursors. GT3 was designed to have a strong affinity gradient between mature bnAbs and gl IGHV1-2 CD4bs Abs, with the hypothesis that such a gradient would facilitate selection of productive SHM on the path to bnAb development (Briney et al., 2016), a hypothesis confirmed in this study.

We also explored whether augmenting the number of antigen-specific CD4 T cells would affect late GC kinetics and accumulation of SHMs. Although increasing Env-specific helper CD4 T cells provided an early competitive benefit for rare $VRC01^{gHL}$ B cells (Lee et al., 2021a), Env-specific CD4 T cells had a minimal effect on B_{GC} cell kinetics and SHM patterns after day 10. These results are consistent with a report showing that B cells receiving more T cell help are preferentially recruited to early GCs but do not have an advantage during competition in established GCs (Yeh et al., 2018). Bolus immunization with the Env trimer typically does not elicit strong GC or T_{FH} cell responses (Hu et al., 2015; Tokatlian et al., 2019). The magnitude of the GC response waned rapidly, starting between day 7 and day 10. Env-specific T_{FH} responses were reduced substantially by day 14, regardless of whether mice received exogenous Env-specific CD4 T cells. Immunization strategies that prolong *in vivo* antigen persistence have been shown to increase GC duration (Cirelli et al., 2019; Moyer et al., 2020); thus, whether modulation of CD4 T cell help influences GC dynamics under conditions where antigen availability is not limiting would be worthy of future exploration.

The results presented here have implications for planning vaccination schedules and determining prime-boost immunogens in future studies. Although our observations were made in the context of a closely controlled mouse model, one shepherding approach may be to maximize B_{GC} affinity maturation from each immunization instead of administering frequent boosts with numerous immunogens. Notably, impressive SHMs were achievable without employing recently described optimized

vaccination strategies (Cirelli et al., 2019; Dubrovskaya et al., 2019; Moyer et al., 2020; Tokatlian et al., 2019) and new adjuvants (Alving et al., 2012; Kasturi et al., 2020; Silva et al., 2021). It may be possible to give rise to breadth-inducing mutations with fewer boosts, and introducing Env trimer-based boost immunogens earlier could be a desirable strategy for doing so.

Limitations of the study

The HC and LC of VRC01^{gHL} BCRs have predetermined CDR3 loops, which derive from the mature VRC01 sequence. Although epitope recognition by VRC01-class bnAbs is much more heavily driven by H-CDR2 rather than H-CDR3, having a mature H-CDR3 loop may have reduced the hurdle for the VRC01^{gHL} B cells to acquire breadth to ΔN276 pseudoviruses. VRC01^{gHL} has a relatively high monomeric K_D of ~100 nM for MD39-GT3.1. The diverse repertoire of authentic VRC01-class human naive B cells had an affinity of 1–40 μM for the eOD-GT8 immunogen (geometric mean, ~4 μM) (Havenar-Daughton et al., 2018; Jardine et al., 2016a).

STAR★METHODS

Detailed methods are provided in the online version of this paper and include the following:

- KEY RESOURCES TABLE
- RESOURCE AVAILABILITY
  - Lead contact
  - Materials availability
  - Data and code availability
- EXPERIMENTAL MODEL AND SUBJECT DETAILS
- METHOD DETAILS
  - Protein expression and purification
  - Size exclusion chromatography with multi-angle light scattering (SEC-MALS)
  - Differential scanning calorimetry (DSC)
  - Mice and immunizations
  - Identification of Env-specific CD4 T cells
  - B cell receptor (BCR) sequencing
  - BCR sequence analysis
  - Biolayer interferometry (BLI)
  - Surface plasmon resonance (SPR)
  - Enzyme-linked immuno-sorbent assay (ELISA)
  - Neutralization assay
- QUANTIFICATION AND STATISTICAL ANALYSIS

SUPPLEMENTAL INFORMATION

Supplemental information can be found online at https://doi.org/10.1016/j.celrep.2022.110485.

ACKNOWLEDGMENTS

We thank the La Jolla Institute for Immunology (LJI) sequencing core for NGS support, LJI Bioinformatics core for coding support, and LJI Flow cytometry core for sorting support. We also thank S. Menis for sharing updated codes for VRC01-class mutation analysis, K. Wiehe for sharing the Singularity container version of ARMADiLLO, and E. Ollmann-Saphire for providing lab equipment for antibody expression. This work was supported by the NIH CHAVD (UM1 AI144462 to S.C., W.R.S., and D.S.), NIH K99 AI145762 (R.K.A.), and the IAVI Neutralizing Antibody Center through the Collaboration for AIDS Vaccine Discovery grant OPP1196345/INV-008813, funded by the Bill and Melinda Gates Foundation (to W.R.S. and D.S.). The full list of IAVI's major donors is available at http://www.iavi.org. The FACSAria II Cell Sorter was acquired through the NIH Shared Instrumentation Grant (SIG) program (S10 RR027366).

AUTHOR CONTRIBUTIONS

Conceptualization, J.H.L. and S.C.; methodology, J.H.L.; formal analysis, J.H.L., E.L., O.K., X.H., and A. Liguori; investigation, J.H.L., C.N., M.A., A. Le, O.K., X.H., and A. Liguori; resources, T.-M.M., B.G., B.A., and W.R.S.; data curation, J.H.L.; writing – original draft, J.H.L.; writing – review & editing, J.H.L., W.R.S., and S.C.; supervision and funding acquisition, D.S., W.R.S., and S.C.

DECLARATION OF INTERESTS

W.R.S. is an inventor on a patent application concerning the eOD-GT5 60-mer and MD39-GT3.1 Env trimer immunogens.

Received: February 28, 2021
Revised: August 10, 2021
Accepted: February 14, 2022
Published: March 8, 2022

REFERENCES

Abbott, R.K., Lee, J.H., Menis, S., Skog, P., Rossi, M., Ota, T., Kulp, D.W., Bhullar, D., Kalyuzhniy, O., Havenar-Daughton, C., et al. (2018). Precursor frequency and affinity determine B cell competitive fitness in germinal centers, tested with germline-targeting HIV vaccine immunogens. Immunity *48*, 133–146.e6.

Alving, C.R., Peachman, K.K., Rao, M., and Reed, S.G. (2012). Adjuvants for human vaccines. Curr. Opin. Immunol. *24*, 310–315.

Arnaout, R., Lee, W., Cahill, P., Honan, T., Sparrow, T., Weiand, M., Nusbaum, C., Rajewsky, K., and Koralov, S.B. (2011). High-resolution description of antibody heavy-chain repertoires in humans. PLoS One *6*, e22365.

Blattner, C., Lee, J.H., Sliepen, K., Derking, R., Falkowska, E., delaPeña, A.T., Cupo, A., Julien, J.P., vanGils, M., Lee, P.S., et al. (2014). Structural delineation of a quaternary, cleavage-dependent epitope at the gp41-gp120 interface on intact HIV-1 Env trimers. Immunity *40*, 669–680.

von Boehmer, L., Liu, C., Ackerman, S., Gitlin, A.D., Wang, Q., Gazumyan, A., and Nussenzweig, M.C. (2016). Sequencing and cloning of antigen-specific antibodies from mouse memory B cells. Nat. Protoc. *11*, 1908–1923.

Bonsignori, M., Zhou, T., Sheng, Z., Chen, L., Gao, F., Joyce, M.G., Ozorowski, G., Chuang, G.Y., Schramm, C.A., Wiehe, K., et al. (2016). Maturation pathway from germline to broad HIV-1 neutralizer of a CD4-mimic antibody. Cell *165*, 449–463.

Bonsignori, M., Scott, E., Wiehe, K., Easterhoff, D., Alam, S.M., Hwang, K.K., Cooper, M., Xia, S.M., Zhang, R., Montefiori, D.C., et al. (2018). Inference of the HIV-1 VRC01 antibody lineage unmutated common ancestor reveals alternative pathways to overcome a key glycan barrier. Immunity *49*, 1162–1174.e8.

Briney, B., Sok, D., Jardine, J.G., Kulp, D.W., Skog, P., Menis, S., Jacak, R., Kalyuzhniy, O., de Val, N., Sesterhenn, F., et al. (2016). Tailored immunogens direct affinity maturation toward HIV neutralizing antibodies. Cell *166*, 1459–1470.e11.

Briney, B., Inderbitzin, A., Joyce, C., and Burton, D.R. (2019). Commonality despite exceptional diversity in the baseline human antibody repertoire. Nature *566*, 393–397.

Burton, D.R. (2017). What are the most powerful immunogen design vaccine strategies? Cold Spring Harb. Perspect. Biol. *9*, a030262.

Burton, D.R., and Hangartner, L. (2016). Broadly neutralizing antibodies to HIV and their role in vaccine design. Annu. Rev. Immunol. *34*, 635–659.

Cao, L., Diedrich, J.K., Kulp, D.W., Pauthner, M., He, L., Park, S.-K.R., Sok, D., Su, C.Y., Delahunty, C.M., Menis, S., et al. (2017). Global site-specific N-glycosylation analysis of HIV envelope glycoprotein. Nat. Commun. *8*, 14913–14954.

Chen, X., Zhou, T., Schmidt, S.D., Duan, H., Cheng, C., Chuang, G., Gu, Y., Louder, M.K., Lin, B.C., Shen, C.-H., et al. (2021). Vaccination induces maturation in a mouse model of diverse unmutated VRC01-class precursors to HIV-neutralizing antibodies with >50% breadth. Immunity *54*, 324–339.e8.

Cirelli, K.M., Carnathan, D.G., Nogal, B., Martin, J.T., Rodriguez, O.L., Upadhyay, A.A., Enemuo, C.A., Gebru, E.H., Choe, Y., Viviano, F., et al. (2019). Slow delivery immunization enhances HIV neutralizing antibody and germinal center responses via modulation of immunodominance. Cell *177*, 1153–1171.e28.

Cottrell, C.A., van Schooten, J., Bowman, C.A., Yuan, M., Oyen, D., Shin, M., Morpurgo, R., van der Woude, P., van Breemen, M., Torres, J.L., et al. (2020). Mapping the immunogenic landscape of near-native HIV-1 envelope trimers in non-human primates. PLoS Pathog. *16*, 1–23.

DeKosky, B.J., Kojima, T., Rodin, A., Charab, W., Ippolito, G.C., Ellington, A.D., and Georgiou, G. (2015). In-depth determination and analysis of the human paired heavy- and light-chain antibody repertoire. Nat. Med. *21*, 86–91.

Diskin, R., Scheid, J.F., Marcovecchio, P.M., West, A.P., Klein, F., Gao, H., Gnanapragasam, P.N.P., Abadir, A., Seaman, M.S., Nussenzweig, M.C., et al. (2011). Increasing the potency and breadth of an HIV antibody by using structure-based rational design. Science *334*, 1289–1293.

Doria-Rose, N.A., Schramm, C.A., Gorman, J., Moore, P.L., Bhiman, J.N., DeKosky, B.J., Ernandes, M.J., Georgiev, I.S., Kim, H.J., Pancera, M., et al. (2014). Developmental pathway for potent V1V2-directed HIV-neutralizing antibodies. Nature *508*, 55–62.

Duan, H., Chen, X., Boyington, J.C., Cheng, C., Zhang, Y., Jafari, A.J., Stephens, T., Tsybovsky, Y., Kalyuzhniy, O., Zhao, P., et al. (2018). Glycan masking focuses immune responses to the HIV-1 CD4-binding site and enhances elicitation of VRC01-class precursor antibodies. Immunity *49*, 301–311.e5.

Dubrovskaya, V., Tran, K., Ozorowski, G., Guenaga, J., Wilson, R., Bale, S., Cottrell, C.A., Turner, H.L., Seabright, G., O'Dell, S., et al. (2019). Vaccination with glycan-modified HIV NFL envelope trimer-liposomes elicits broadly neutralizing antibodies to multiple sites of vulnerability. Immunity *51*, 915–929.e7.

Escolano, A., Steichen, J.M., Dosenovic, P., Kulp, D.W., Golijanin, J., Sok, D., Freund, N.T., Gitlin, A.D., Oliveira, T., Araki, T., et al. (2016). Sequential immunization elicits broadly neutralizing anti-HIV-1 antibodies in Ig knockin mice. Cell *166*, 1445–1458.e12.

Falkowska, E., Le, K.M., Ramos, A., Doores, K.J., Lee, J.H., Blattner, C., Ramirez, A., Derking, R., vanGils, M.J., Liang, C.H., et al. (2014). Broadly neutralizing HIV antibodies define a glycan-dependent epitope on the prefusion conformation of gp41 on cleaved envelope trimers. Immunity *40*, 657–668.

Freund, N.T., Wang, H., Scharf, L., Nogueira, L., Horwitz, J.A., Bar-On, Y., Golijanin, J., Sievers, S.A., Sok, D., Cai, H., et al. (2017). Coexistence of potent HIV-1 broadly neutralizing antibodies and antibody-sensitive viruses in a viremic controller. Sci. Transl. Med. *9*, 1–14.

Havenar-Daughton, C., Sarkar, A., Kulp, D.W., Toy, L., Hu, X., Deresa, I., Kalyuzhniy, O., Kaushik, K., Upadhyay, A.A., Menis, S., et al. (2018). The human naive B cell repertoire contains distinct subclasses for a germline-targeting HIV-1 vaccine immunogen. Sci. Transl. Med. *10*, eaat0381.

Hoffenberg, S., Powell, R., Carpov, A., Wagner, D., Wilson, A., Kosakovsky Pond, S., Lindsay, R., Arendt, H., DeStefano, J., Phogat, S., et al. (2013). Identification of an HIV-1 clade A envelope that exhibits broad antigenicity and neutralization sensitivity and elicits antibodies targeting three distinct epitopes. J. Virol. *87*, 5372–5383.

Hu, J.K., Crampton, J.C., Cupo, A., Ketas, T., van Gils, M.J., Sliepen, K., de Taeye, S.W., Sok, D., Ozorowski, G., Deresa, I., et al. (2015). Murine antibody responses to cleaved soluble HIV-1 envelope trimers are highly restricted in specificity. J. Virol. *89*, 10383–10398.

Huang, D., Abbott, R., Havenar-Daughton, C., Skog, P., Al-Kolla, R., Groschel, B., Blane, T., Menis, S., Tran, J.T., Thinnes, T., et al. (2020). B cells expressing authentic naive human VRC01-class BCRs can be primed and recruited to germinal centers in multiple independent mouse models. Proc. Natl. Acad. Sci. U S A *117*, 22920–22931.

Huang, J., Kang, B.H., Ishida, E., Zhou, T., Griesman, T., Sheng, Z., Wu, F., Doria-Rose, N.A., Zhang, B., McKee, K., et al. (2016). Identification of a CD4-binding-site antibody to HIV that evolved near-Pan neutralization breadth. Immunity *45*, 1108–1121.

Jardine, J., Julien, J.-P., Menis, S., Ota, T., Kalyuzhniy, O., McGuire, A., Sok, D., Huang, P.-S., MacPherson, S., Jones, M., et al. (2013). Rational HIV immunogen design to target specific germline B cell receptors. Science *340*, 711–716.

Jardine, J.G., Ota, T., Sok, D., Pauthner, M., Kulp, D.W., Kalyuzhniy, O., Skog, P.D., Thinnes, T.C., Bhullar, D., Briney, B., et al. (2015). HIV-1 VACCINES. Priming a broadly neutralizing antibody response to HIV-1 using a germline-targeting immunogen. Science *349*, 156–161.

Jardine, J.G., Kulp, D.W., Havenar-Daughton, C., Sarkar, A., Briney, B., Sok, D., Sesterhenn, F., Ereño-Orbea, J., Kalyuzhniy, O., Deresa, I., et al. (2016a). HIV-1 broadly neutralizing antibody precursor B cells revealed by germline-targeting immunogen. Science *351*, 1458–1463.

Jardine, J.G., Sok, D., Julien, J.P., Briney, B., Sarkar, A., Liang, C.H., Scherer, E.A., Henry Dunand, C.J., Adachi, Y., Diwanji, D., et al. (2016b). Minimally mutated HIV-1 broadly neutralizing antibodies to guide reductionist vaccine design. PLoS Pathog. *12*, 1–33.

Kasturi, S.P., Ur Rasheed, M.A., Havenar-Daughton, C., Pham, M., Legere, T., Sher, Z.J., Kovalenkov, Y., Gumber, S., Huang, J.Y., Gottardo, R., et al. (2020). 3M-052, a synthetic TLR-7/8 agonist, induces durable HIV-1 envelope-specific plasma cells and humoral immunity in nonhuman primates. Sci. Immunol. *5*, eabb1025.

Kato, Y., Abbott, R.K., Freeman, B.L., Haupt, S., Groschel, B., Silva, M., Menis, S., Irvine, D.J., Schief, W.R., and Crotty, S. (2020). Multifaceted effects of antigen valency on B cell response composition and differentiation in vivo. Immunity *53*, 548–563.e8.

Klasse, P.J., Ketas, T.J., Cottrell, C.A., Ozorowski, G., Debnath, G., Camara, D., Francomano, E., Pugach, P., Ringe, R.P., LaBranche, C.C., et al. (2018). Epitopes for neutralizing antibodies induced by HIV-1 envelope glycoprotein BG505 SOSIP trimers in rabbits and macaques. PLoS Pathog. *14*, e1006913.

Kong, L., Ju, B., Chen, Y., He, L., Ren, L., Liu, J., Hong, K., Su, B., Wang, Z., Ozorowski, G., et al. (2016). Key gp120 glycans pose roadblocks to the rapid development of VRC01-class antibodies in an HIV-1-Infected Chinese donor. Immunity *44*, 939–950.

Kong, R., Duan, H., Sheng, Z., Xu, K., Acharya, P., Chen, X., Cheng, C., Dingens, A.S., Gorman, J., Sastry, M., et al. (2019). Antibody lineages with vaccine-induced antigen-binding hotspots develop broad HIV neutralization. Cell *178*, 567–584.e19.

LaBranche, C.C., McGuire, A.T., Gray, M.D., Behrens, S., Zhou, T., Sattentau, Q.J., Peacock, J., Eaton, A., Greene, K., Gao, H., et al. (2018). HIV-1 envelope glycan modifications that permit neutralization by germline-reverted VRC01-class broadly neutralizing antibodies. PLoS Pathog. *14*, e1007431.

Landais, E., and Moore, P.L. (2018). Development of broadly neutralizing antibodies in HIV-1 infected elite neutralizers. Retrovirology *15*, 1–14.

Landais, E., Huang, X., Havenar-Daughton, C., Murrell, B., Price, M.A., Wickramasinghe, L., Ramos, A., Bian, C.B., Simek, M., Allen, S., et al. (2016). Broadly neutralizing antibody responses in a large longitudinal sub-saharan HIV primary infection cohort. PLoS Pathog. *12*, e1005369.

Landais, E., Murrell, B., Briney, B., Murrell, S., Rantalainen, K., Berndsen, Z.T., Ramos, A., Wickramasinghe, L., Smith, M.L., Eren, K., et al. (2017). HIV envelope glycoform heterogeneity and localized diversity govern the initiation and maturation of a V2 apex broadly neutralizing antibody lineage. Immunity *47*, 990–1003.e9.

Lee, J.H., Hu, J.K., Georgeson, E., Nakao, C., Groschel, B., Dileepan, T., Jenkins, M.K., Seumois, G., Vijayanand, P., Schief, W.R., et al. (2021a).

Modulating the quantity of HIV Env-specific CD4 T cell help promotes rare B cell responses in germinal centers. J. Exp. Med. *218*, e20201254.

Lee, J.H., Toy, L., Kos, J.T., Safonova, Y., Schief, W.R., Havenar-Daughton, C., Watson, C.T., and Crotty, S. (2021b). Vaccine genetics of IGHV1-2 VRC01-class broadly neutralizing antibody precursor naïve human B cells. NPJ Vaccines *6*, 113.

Lyumkis, D., Julien, J.-P., De Val, N., Cupo, A., Potter, C.S., Klasse, J., Burton, D.R., Sanders, R.W., Moore, J.P., Carragher, B., et al. (2013). Cryo-EM structure of a fully glycosylated soluble cleaved HIV-1 envelope trimer. Science *342*, 1484–1490.

MacLeod, D.T., Choi, N.M., Briney, B., Garces, F., Ver, L.S., Landais, E., Murrell, B., Wrin, T., Kilembe, W., Liang, C.H., et al. (2016). Early antibody lineage diversification and independent limb maturation lead to broad HIV-1 neutralization targeting the Env high-mannose patch. Immunity *44*, 1215–1226.

McCoy, L.E., van Gils, M.J., Ozorowski, G., Messmer, T., Briney, B., Voss, J.E., Kulp, D.W., Macauley, M.S., Sok, D., Pauthner, M., et al. (2016). Holes in the glycan shield of the native HIV envelope are a target of trimer-elicited neutralizing antibodies. Cell Rep. *16*, 2327–2338.

McGuire, A.T., Dreyer, A.M., Carbonetti, S., Lippy, A., Glenn, J., Scheid, J.F., Mouquet, H., and Stamatatos, L. (2014). Antigen modification regulates competition of broad and narrow neutralizing HIV antibodies. Science *346*, 1380–1383.

Medina-Ramírez, M., Garces, F., Escolano, A., Skog, P., de Taeye, S.W., Del Moral-Sanchez, I., McGuire, A.T., Yasmeen, A., Behrens, A.-J., Ozorowski, G., et al. (2017). Design and crystal structure of a native-like HIV-1 envelope trimer that engages multiple broadly neutralizing antibody precursors in vivo. J. Exp. Med. *214*, 2573–2590.

Mesin, L., Ersching, J., and Victora, G.D. (2016). Germinal center B cell dynamics. Immunity *45*, 471–482.

Moyer, T.J., Kato, Y., Abraham, W., Chang, J.Y.H., Kulp, D.W., Watson, N., Turner, H.L., Menis, S., Abbott, R.K., Bhiman, J.N., et al. (2020). Engineered immunogen binding to alum adjuvant enhances humoral immunity. Nat. Med. *26*, 430–440.

Okonechnikov, K., Golosova, O., Fursov, M., Varlamov, A., Vaskin, Y., Efremov, I., German Grehov, O.G., Kandrov, D., Rasputin, K., Syabro, M., et al. (2012). Unipro UGENE: a unified bioinformatics toolkit. Bioinformatics *28*, 1166–1167.

Parks, K.R., MacCamy, A.J., Trichka, J., Gray, M., Weidle, C., Borst, A.J., Khechaduri, A., Takushi, B., Agrawal, P., Guenaga, J., et al. (2019). Overcoming steric restrictions of VRC01 HIV-1 neutralizing antibodies through immunization. Cell Rep. *29*, 3060–3072.e7.

Rettig, T.A., Ward, C., Bye, B.A., Pecaut, M.J., and Chapes, S.K. (2018). Characterization of the naive murine antibody repertoire using unamplified high-throughput sequencing. PLoS One *13*, 1–20.

Ringe, R.P., Pugach, P., Cottrell, C.A., LaBranche, C.C., Seabright, G.E., Ketas, T.J., Ozorowski, G., Kumar, S., Schorcht, A., van Gils, M.J., et al. (2019). Closing and opening holes in the glycan shield of HIV-1 envelope glycoprotein SOSIP trimers can redirect the neutralizing antibody response to the newly unmasked epitopes. J. Virol. *93*, 3–26.

Saunders, K.O., Wiehe, K., Tian, M., Acharya, P., Bradley, T., Munir Alam, S., Go, E.P., Scearce, R., Sutherland, L., Henderson, R., et al. (2019). Targeted selection of HIV-specific antibody mutations by engineering B cell maturation. Science *366*, eaay7199.

Seaman, M.S., Janes, H., Hawkins, N., Grandpre, L.E., Devoy, C., Giri, A., Coffey, R.T., Harris, L., Wood, B., Daniels, M.G., et al. (2010). Tiered categorization of a diverse panel of HIV-1 Env pseudoviruses for assessment of neutralizing antibodies. J. Virol. *84*, 1439–1452.

Silva, M., Kato, Y., Melo, M.B., Phung, I., Freeman, B.L., Li, Z., Roh, K., Van Wijnbergen, J.W., Watkins, H., Enemuo, C.A., et al. (2021). A particulate saponin/TLR agonist vaccine adjuvant alters lymph flow and modulates adaptive immunity. Sci. Immunol. *6*, eabf1152.

Simek, M.D., Rida, W., Priddy, F.H., Pung, P., Carrow, E., Laufer, D.S., Lehrman, J.K., Boaz, M., Tarragona-Fiol, T., Miiro, G., et al. (2009). Human immunodeficiency virus type 1 elite neutralizers: individuals with broad and potent neutralizing activity identified by using a high-throughput neutralization assay together with an analytical selection algorithm. J. Virol. *83*, 7337–7348.

Sok, D., and Burton, D.R. (2018). Recent progress in broadly neutralizing antibodies to HIV. Nat. Immunol. *19*, 1179–1188.

Sok, D., Gils, M.J.V., Pauthner, M., Julien, J.P., Saye-Francisco, K.L., Hsueh, J., Briney, B., Lee, J.H., Le, K.M., Lee, P.S., et al. (2014). Recombinant HIV envelope trimer selects for quaternary-dependent antibodies targeting the trimer apex. Proc. Natl. Acad. Sci. U S A *111*, 17624–17629.

Stamatatos, L., Pancera, M., and McGuire, A.T. (2017). Germline-targeting immunogens. Immunol. Rev. *275*, 203–216.

Steichen, J.M., Kulp, D.W., Tokatlian, T., Escolano, A., Dosenovic, P., Stanfield, R.L., McCoy, L.E., Ozorowski, G., Hu, X., Kalyuzhniy, O., et al. (2016). HIV vaccine design to target germline precursors of glycan-dependent broadly neutralizing antibodies. Immunity *45*, 483–496.

Steichen, J.M., Lin, Y.-C., Havenar-Daughton, C., Pecetta, S., Ozorowski, G., Willis, J.R., Toy, L., Sok, D., Liguori, A., Kratochvil, S., et al. (2019). A generalized HIV vaccine design strategy for priming of broadly neutralizing antibody responses. Science *366*, 1–15.

Struwe, W.B., Chertova, E., Allen, J.D., Seabright, G.E., Watanabe, Y., Harvey, D.J., Medina-Ramírez, M., Roser, J.D., Smith, R., Westcott, D., et al. (2018). Site-specific glycosylation of virion-derived HIV-1 Env is mimicked by a soluble trimeric immunogen. Cell Rep. *24*, 1958–1966.e5.

Tian, M., Cheng, C., Chen, X., Duan, H., Cheng, H.-L., Dao, M., Sheng, Z., Kimble, M., Wang, L., Lin, S., et al. (2016). Induction of HIV neutralizing antibody lineages in mice with diverse precursor repertoires. Cell *166*, 1471–1484.e18.

Tokatlian, T., Read, B.J., Jones, C.A., Kulp, D.W., Menis, S., Chang, J.Y.H., Steichen, J.M., Kumari, S., Allen, J.D., Dane, E.L., et al. (2019). Innate immune recognition of glycans targets HIV nanoparticle immunogens to germinal centers. Science *363*, 649–654.

Umotoy, J., Bagaya, B.S., Joyce, C., Schiffner, T., Menis, S., Saye-Francisco, K.L., Biddle, T., Mohan, S., Vollbrecht, T., Kalyuzhniy, O., et al. (2019). Rapid and focused maturation of a VRC01-class HIV broadly neutralizing antibody lineage involves both binding and accommodation of the N276-glycan. Immunity *51*, 141–154.e6.

Walker, L.M., Phogat, S.K., Chan-Hui, P.-Y., Wagner, D., Phung, P., Goss, J.L., Wrin, T., Simek, M.D., Fling, S., Mitcham, J.L., et al. (2009). Broad and potent neutralizing antibodies from an African donor reveal a new HIV-1 vaccine target. Science *326*, 285–289.

Walker, L.M., Huber, M., Doores, K.J., Falkowska, E., Pejchal, R., Julien, J.P., Wang, S.K., Ramos, A., Chan-Hui, P.Y., Moyle, M., et al. (2011). Broad neutralization coverage of HIV by multiple highly potent antibodies. Nature *477*, 466–470.

West, A.P., Diskin, R., Nussenzweig, M.C., and Björkman, P.J. (2012). Structural basis for germ-line gene usage of a potent class of antibodies targeting the CD4-binding site of HIV-1 gp120. Proc. Natl. Acad. Sci. U S A *109*, E2083–E2090.

Wiehe, K., Bradley, T., Meyerhoff, R.R., Hart, C., Williams, W.B., Easterhoff, D., Faison, W.J., Kepler, T.B., Saunders, K.O., Alam, S.M., et al. (2018). Functional relevance of improbable antibody mutations for HIV broadly neutralizing antibody development. Cell Host Microbe *23*, 759–765.e6.

Xu, K., Acharya, P., Kong, R., Cheng, C., Chuang, G.Y., Liu, K., Louder, M.K., O'Dell, S., Rawi, R., Sastry, M., et al. (2018). Epitope-based vaccine design yields fusion peptide-directed antibodies that neutralize diverse strains of HIV-1. Nat. Med. *24*, 857–867.

Yeh, C.-H., Nojima, T., Kuraoka, M., and Kelsoe, G. (2018). Germinal center entry not selection of B cells is controlled by peptide-MHCII complex density. Nat. Commun. *9*, 1–11.

Zhao, F., Joyce, C., Burns, A., Nogal, B., Cottrell, C.A., Ramos, A., Biddle, T., Pauthner, M., Nedellec, R., Qureshi, H., et al. (2020). Mapping neutralizing antibody epitope specificities to an HIV Env trimer in immunized and in infected rhesus macaques. Cell Rep. *32*, 108122.

Zhou, T., Georgiev, I., Wu, X., Yang, Z.Y., Dai, K., Finzi, A., Kwon, Y., Scheid, J.F., Shi, W., Xu, L., et al. (2010). Structural basis for broad and potent neutralization of HIV-1 by antibody VRC01. Science *329*, 811–817.

Zhou, T., Zhu, J., Wu, X., Moquin, S., Zhang, B., Acharya, P., Georgiev, I.S., Altae-Tran, H.R., Chuang, G.-Y., Joyce, M.G., et al. (2013). Multidonor analysis reveals structural elements, genetic determinants, and maturation pathway for HIV-1 neutralization by VRC01-class antibodies. Immunity *39*, 245–258.

Zhou, T., Lynch, R.M., Chen, L., Acharya, P., Wu, X., Doria-Rose, N.A., Joyce, M.G., Lingwood, D., Soto, C., Bailer, R.T., et al. (2015). Structural repertoire of HIV-1-Neutralizing antibodies targeting the CD4 supersite in 14 donors. Cell *161*, 1280–1292.

STAR★METHODS

KEY RESOURCES TABLE

REAGENT or RESOURCE	SOURCE	IDENTIFIER
Antibodies		
Rat anti-mouse CD4 Alexa Fluor 700 (Clone: GK1.5)	BioLegend	Cat# 100430; RRID: AB_493699
Rat anti-mouse/human CD45R/B220 BV785 (Clone: RA3-6B2)	BioLegend	Cat# 103246; RRID: AB_2563256
Rat anti-mouse/human CD45R/B220 BV421 (Clone: RA3-6B2)	BioLegend	Cat# 103251; RRID: AB_2562905
Rat anti-mouse CXCR5 biotin (Clone: L138D7)	BioLegend	Cat# 145510; RRID: AB_2562126
eBioscience Rat anti-mouse CXCR5 biotin (Clone: SPRCL5)	Thermo Fisher Scientific	Cat# 12-7185-82; RRID: AB_2572800
Mouse anti-rat CD90/mouse CD90.1 Alexa Fluor 488 (Clone: OX-7)	BioLegend	Cat# 202506; RRID: AB_492882
Mouse anti-rat CD90/mouse CD90.1 BV650 (Clone: OX-7)	BioLegend	Cat# 202533; RRID: AB_2562254
eBioscience Rat anti-mouse/rat FOXP3 PE-Cy7 (Clone: FJK-16s)	Thermo Fisher Scientific	Cat# 25-5773-82; RRID: AB_891552
Mouse anti-human Bcl6 Alexa Fluor 647 (Clone: K112-91)	BD Biosciences	Cat# 561525; RRID: AB_10898007
Rat anti-mouse/human GL7 PerCP-Cy5.5 (Clone: GL7)	BioLegend	Cat# 144610; RRID: AB_2562979
Hamster anti-mouse CD95 BV510 (Clone: Jo2)	BD Biosciences	Cat# 563646; RRID: AB_2738345
Hamster anti-mouse CD95 PE (Clone: Jo2)	BD Biosciences	Cat# 554258; RRID: AB_395330
Rat anti-mouse IgD PE-Cy7 (Clone: 11–26c.2a)	BioLegend	Cat# 405720; RRID: AB_2561876
Rat anti-mouse CD138 BV650 (Clone: 281–2)	BioLegend	Cat# 142518; RRID: AB_2650927
Rat anti-mouse PD-1 BV605 (Clone: 29F.1A12)	BioLegend	Cat# 135220; RRID: AB_2562616
Rat anti-mouse CD8a APC/Fire 750 (Clone 53–6.7)	BioLegend	Cat# 100766; RRID: AB_2572113
Rat anti-mouse CD4 APC/Fire 750 (Clone: GK1.5)	BioLegend	Cat# 100460; RRID: AB_2572111
Rat anti-mouse Ly-6G/Ly-6C APC/Fire 750 (Clone RB6-8C5)	BioLegend	Cat# 108456; RRID: AB_2616737
Mouse anti-mouse NK-1.1 APC/Fire 750 (Clone: S17016D)	BioLegend	Cat# 156516; RRID: AB_2892323
Rat anti-mouse CD3 APC/Fire 750 (Clone: 17A2)	BioLegend	Cat# 100248; RRID: AB_2572118
Rat anti-mouse/human CD44 PerCP-Cy5.5 (Clone: IM7)	BioLegend	Cat# 103032; RRID: AB_2076204
Rat anti-mouse CD62L BV510 (Clone: MEL-14)	BD Biosciences	Cat# 563117; RRID: AB_2738013
Mouse anti-mouse CD45.1 BUV395 (Clone: A20)	BD Biosciences	Cat# 565212; RRID: AB_2722493
Mouse anti-mouse CD45.2 BUV395 (Clone: 104)	BD Biosciences	Cat# 564616; RRID: AB_2738867

(*Continued on next page*)

CellPress
OPEN ACCESS

Continued

REAGENT or RESOURCE	SOURCE	IDENTIFIER
Hamster anti-mouse CD69 Alexa Fluor 488 (Clone: H1.2F3)	BD Biosciences	Cat# 104516; RRID: AB_492845
Ultra-LEAF purified hamster anti-mouse CD154 (Clone: MR1)	BioLegend	Cat# 106517; RRID: AB_2813947
Mouse anti-mouse CD45.1 FITC (Clone: A20)	BioLegend	Cat# 110706; RRID: AB_313495
Mouse anti-mouse CD45.2 Alexa Fluor 647 (Clone: 104)	BioLegend	Cat# 109818 RRID: AB_492870
Mouse anti-mouse TCR Vβ12 PE (Clone: MR11-1)	BioLegend	Cat# 139704; RRID: AB_10639729
Purified rat anti-mouse CD16/Cd32 (Mouse BD Fc Block^{TM}) (Clone: 2.4G2)	BD Biosciences	Cat# 553141; RRID: AB_394656
Peroxidase AffiniPure donkey anti-human IgG (H + L)	Jackson ImmunoResearch	Cat# 709-035-149; RRID: AB_2340495
Peroxidase AffiniPure goat anti-mouse IgG (H + L)	Jackson ImmunoResearch	Cat# 115-035-166; RRID: AB_2338511
Peroxidase AffiniPure goat anti-human IgG, Fcγ fragment specific	Jackson ImmunoResearch	Cat# 109-035-098; RRID: AB_2337586
TotalSeq-C0301 anti-mouse Hashtag 1 Antibody (Clone: M1/42, 30-F11)	BioLegend	Cat# 155861; RRID: AB_2800693
TotalSeq-C0302 anti-mouse Hashtag 1 Antibody (Clone: M1/42, 30-F11)	BioLegend	Cat# 155863; RRID: AB_2800694
TotalSeq-C0303 anti-mouse Hashtag 1 Antibody (Clone: M1/42, 30-F11)	BioLegend	Cat# 155865; RRID: AB_2800695
TotalSeq-C0304 anti-mouse Hashtag 1 Antibody (Clone: M1/42, 30-F11)	BioLegend	Cat# 155867; RRID: AB_2800696
TotalSeq-C0305 anti-mouse Hashtag 1 Antibody (Clone: M1/42, 30-F11)	BioLegend	Cat# 155869; RRID: AB_2800697
Bacterial and virus strains		
NEB 5-alpha Competent *E. coli* (High Efficiency)	New England BioLabs	Cat# C2987H
92TH021 from 6 HIV-1 Env-pseudotyped virus panel with N276 glycan mutation	Elise Landais IAVI-NAC (elandais@iavi.org)	Simek et al. (2009)
Viruses from tiered HIV-1 Env-pseudotyped viruses with N276 glycan mutation	Elise Landais IAVI-NAC (elandais@iavi.org)	Seaman et al. (2010)
BG505.W6M.ENV.C2 (BG505) with N276 glycan mutations	Elise Landais IAVI-NAC (elandais@iavi.org)	Hoffenberg et al. (2013)
Chemicals, peptides, and recombinant proteins		
BG505 MD39-GT3.1 SOSIP	This paper	Produced in house
BG505 SOSIPv4.1-GT1	Medina-Ramírez et al. (2017)	Produced in house
BG505 MD39 SOSIP	Steichen et al. (2016)	Produced in house
BG505 MD39 SOSIP-Biotin	Steichen et al. (2016)	Produced in house
BG505 MD39-N276D SOSIP-biotin	This paper	Produced in house
BG505 MD39 Ferritin nanoparticle	This paper and Tokatlian et al. (2019)	Produced in house
BG505 MD39-N276D Ferritin nanoparticle	This paper and Tokatlian et al. (2019)	Produced in house
BG505 MD39-N276A Ferritin nanoparticle	This paper and Tokatlian et al. (2019)	Produced in house
RM19R Fab	Cottrell et al. (2020)	Produced in house
PKVSFEPIPIHYCAP	A&A Labs LLC	Custom
BG505-MD39 SOSIP peptide megapool	A&A Labs LLC	Custom
Pierce Protein A Agarose	Thermo Fisher Scientific	Cat# 20334
CaptureSelect CH1-XL Affinity Matrix	Thermo Fisher Scientific	Cat# 1943462005

(*Continued on next page*)

Continued		
REAGENT or RESOURCE	SOURCE	IDENTIFIER
Sigma Adjuvant System	Sigma Aldrich	Cat# S6322-1VL
Alhydrogel adjuvant 2%	InvivoGen	Cat# vac-alu-250
1-Step Ultra TMB-ELISA Substrate Solution	Thermo Fisher Scientific	Cat# 34028
TMB Chromogen Solution (for ELISA)	Thermo Fisher Scientific	Cat# 002023
Phosphatase substrate	Sigma Aldrich	Cat# S0942
Bovine Serum Albumin	Sigma Aldrich	Cat# A7030-5KG
Fetal Bovine Serum	Thermo Fisher Scientific	Cat# 16000044
BD Difco^{TM} Skim Milk	BD Life Sciences	Cat# 232100
Sulfuric Acid, 2.00 Normal	RICCA Chemical Company	Cat# 8310–32
Streptavidin	Jackson ImmunoResearch	Cat# 016-000-084; RRID: AB_2337233
Lectin from Galanthus nivalis (snowdrop), lyophilized powder	Sigma-Aldrich	Cat# L8275-5mg
HBS-EP+ 20×, pH 7.6	Teknova	Cat# H8022
FuGENE 6 Transfection Reagent	Promega	Cat# E2692
Octet Kinetics Buffer 10X	Sartorius	Cat# 18–1105
BV421 Streptavidin	BioLegend	Cat# 405225
eBioscience Propidium Iodide Staining Solution	Thermo Fisher Scientific	Cat# 00-6990-50
eBioscience Fixable Viability Dye eFluor 780	Thermo Fisher Scientific	Cat# 65-0865-14
M1 antibody	This paper	Produced in house
M2 antibody	This paper	Produced in house
M3 antibody	This paper	Produced in house
M4 antibody	This paper	Produced in house
M5 antibody	This paper	Produced in house
M6 antibody	This paper	Produced in house
M7 antibody	This paper	Produced in house
M8 antibody	This paper	Produced in house
M9 antibody	This paper	Produced in house
M10 antibody	This paper	Produced in house
M11 antibody	This paper	Produced in house
M12 antibody	This paper	Produced in house
M13 antibody	This paper	Produced in house
E1 antibody	Abbott et al. (2018)	Produced in house
E2 antibody	Abbott et al. (2018)	Produced in house
E3 antibody	Abbott et al. (2018)	Produced in house
E4 antibody	Abbott et al. (2018)	Produced in house
E5 antibody	Abbott et al. (2018)	Produced in house
E6 antibody	Abbott et al. (2018)	Produced in house
E7 antibody	Abbott et al. (2018)	Produced in house
E8 antibody	Abbott et al. (2018)	Produced in house
E9 antibody	Abbott et al. (2018)	Produced in house
E10 antibody	Abbott et al. (2018)	Produced in house
E11 antibody	Abbott et al. (2018)	Produced in house
Critical commercial assays		
Pierce Fab Preparation Kit	Thermo Fisher Scientific	Cat# 44985
eBioscience FoxP3/Transcription Factor Staining Buffer Set	Thermo Fisher Scientific	Cat# 00-5523-00
BD Cytofix Fixation Buffer	BD Biosciences	Cat# 554655

(*Continued on next page*)

CellPress
OPEN ACCESS

Continued

REAGENT or RESOURCE	SOURCE	IDENTIFIER
Luciferase Assay System	Promega	Cat# E4550
EasySep Mouse CD4+ T Cell Isolation Kit	STEMCELL Technologies	Cat# 19852
EasySep Mouse B Cell Isolation Kit	STEMCELL Technologies	Cat# 19854
Alexa Fluor 647 Protein Labeling Kit	Thermo Fisher Scientific	Cat# A20173
Chromium Single Cell V(D)J Enrichment Kit, Mouse B Cell, 96 rxns	10X Genomics	Cat# 1000072
Chromium Single Cell 5′ Library & Gel Bead Kit, 4 rxns	10X Genomics	Cat# 1000014
Chromium Single Cell A Chip Kit, 16 rxns	10X Genomics	Cat# 1000009
Chromium Single Cell 5′ Feature Barcode Library Kit, 16 rxns	10X Genomics	Cat# 1000080
Chromium i7 Multiplex Kit, 96 rxns	10X Genomics	Cat# 120262
Chromium i7 Multiplex Kit N Set A, 96 rxns	10X Genomics	Cat# 1000084
SuperScript II Reverse Transcriptase	Thermo Fisher	Cat# 18064071
Phusion Green High-Fidelity DNA Polymerase	Thermo Fisher	Cat# F534L
Hot StarTaq Master Mix Kit (2500 U)	Qiagen	Cat# 203446
ExpiCHO Expression System Kit	Thermo Fisher Scientific	Cat# A29133
Human Antibody Capture Kit	Cytvia	Cat# BR100839
BirA biotin-protein ligase standard reaction kit	Avidity Inc	Cat# BirA500
Deposited data		
VRC01gHL HC sequences	This Paper	Genbank: OM484270 – OM484649
VRC01gHL LC sequences	This Paper	Genbank: OM484650 – OM484939
10X Genomics BCR sequencing data	This Paper	BioProject: PRJNA802246
Experimental models: Cell lines		
Human: HeLa-derived TZM-bl	NIH AIDS Reagent Program	Cat# 8129–442; RRID: CVCL_B478
Human: HEK293T	ATCC	Cat# CRL-3216; RRID: CVCL_0063
Human: HEK293S GnTI−/−	ATCC	Cat# CRL-3022; RRID: CVCL_A785
Human: FreeStyle 293F	Thermo Fisher Scientific	Cat# R79007; RRID: CVCL_D603
Hamster: ExpiCHO-S	Thermo Fisher Scientific	Cat# A29132; RRID: CVCL_5J31
Experimental models: Organisms/strains		
Mouse: C57BL/6J mice	The Jackson Laboratory	JAX: 000664
Mouse: VRC01^{gHL}	Abbott et al. (2018)	NA
Mouse: B6.SJL-*Ptprc*^{a}*Pepc*^{b}/BoyJ	The Jackson Laboratory	JAX: 002014
Mouse: B6.PL-*Thy1*^{a}/CyJ	The Jackson Laboratory	JAX: 000406
Mouse: HYCAP1	Lee et al., 2021	NA
Mouse: HYCAP3	Lee et al., 2021	NA
Oligonucleotides		
1st PCR mouse IgH forward primer 1mFH_VII: CCTGTCAGTAACTRCAGGTGTCC	von Boehmer et al. (2016)	NA
1st PCR mouse IgG constant region reverse primer 1mRG: AGAAGGTGTGCACACCGCTGGAC	von Boehmer et al. (2016)	NA
1st PCR mouse IgK forward primer 1mFK_I: RGTGCAGATTTTCAGCTTCCTGCT	von Boehmer et al. (2016)	NA
1st PCR mouse IgK constant region reverse primer 1mRK: ACTGAGGCACCTCCAGATGTT	von Boehmer et al. (2016)	NA

(*Continued on next page*)

REAGENT or RESOURCE	SOURCE	IDENTIFIER
Continued		
2nd PCR mouse IgH forward primer 2mFG: GGGAATTCGAGGTGCAGCTG CAGGAGTCTGG	von Boehmer et al. (2016)	NA
2nd PCR mouse IgG constant region reverse primer 2mRG: GCTCAGGGAARTAGCCCTTGAC	von Boehmer et al. (2016)	NA
2nd PCR mouse IgK forward primer 2mFK: GAYATTGTGMTSACMCARWCTMCA	von Boehmer et al. (2016)	NA
2nd PCR mouse IgK reverse primer 2mRK: TGGGAAGATGGATACAGTT	von Boehmer et al. (2016)	NA
Human IGKV3-11 FWR2 specific forward sequencing primer: GAAATTGTGTTGACACAGTCTCC	Abbott et al. (2018)	NA
VRC01 LCDR3 specific reverse sequencing primer: CGAAGAACTCGTACTGCTGAC	Abbott et al. (2018)	NA
Software and algorithms		
Cell Ranger	10X Genomics	https://support.10xgenomics.com/ingles-cell-gene-expression/software/overview/welcome
Hashtag count	Lee et al., 2021b	https://github.com/jvxtaposed/Filter-Cellranger-VDJ
ARMADiLLO	Wiehe et al. (2018)	Software provided by publisher
Unipro UGENE	Okonechnikov et al. (2012)	http://ugene.net
Data Analysis HT software v11.1	Sartorius	https://www.sartorius.com
ProteOn™ Manager Software	Bio-Rad Laboratories	https://bio-rad.com
FlowJo v10	FlowJo	https://www.flowjo.com/
Adobe Illustrator CS	Adobe	https://www.adobe.com
Prism 8	GraphPad	https://www.graphpad.com
Microsoft Office Excel	Microsoft	https://www.microsoft.com
FACSDiva	BD Bioscience	https://www.bdbiosciences.com
Other		
Octet SA Biosensors	Sartorius	Cat# SA
Costar Assay Plate, 96 well flat-bottom, half area, high binding	Corning	Cat# 3690

RESOURCE AVAILABILITY

Lead contact

Further information and requests for resources and reagents should be directed to and will be fulfilled by the lead contact, Shane Crotty (shane@lji.org).

Materials availability

The reagents and mice generated in this study may be made available on request upon completing a Material Transfer Agreement.

Data and code availability

- Paired VRC01^{gHL} BCR sequence data obtained by Sanger sequencing have been deposited in GenBank: OM484270 – OM484939. 10X Genomics BCR sequencing FASTQ data are submitted under BioProject: PRJNA802246. FCS files reported in this paper will be shared by the lead contact upon request.
- This paper does not report original code. The code used in the analysis of BCR sequences are publicly available through the 10X Genomics CellRanger software, or through published manuscripts and are reported in the relevant headings in the method details section.
- Any additional information required to reanalyze the data reported in this paper is available from the lead contact upon request.

EXPERIMENTAL MODEL AND SUBJECT DETAILS

Mouse experiments were all performed at the La Jolla Institute for Immunology (LJI). All experimental procedures were approved by the IACUC committee of LJI. Experiments were performed using sex-and age-matched mice between 7 and 12 weeks of age. Both male and female mice were used, although all mice were sex-matched within a given replicate. 6- to 7-week-old C57BL/6J (B6) mice were purchased from Jackson Laboratory (JAX: 000664) and housed at LJI. Mice were rested for least a week upon receipt prior to use. All other mouse strains were maintained at LJI. A colony of B6.SJL-*Ptprc*^{a}*Pepc*^{b}/BoyJ (B6.CD45.1) mice originally purchased from Jackson Laboratory (JAX: 002014) were maintained at LJI. VRC01^{gHL} mice heterozygous for inferred germline reverted VRC01 IgH (VRC01^{gH}) and VRC01 IgL (VRC01^{gL}) (Abbott et al., 2018) were maintained on a B6 or B6.CD45.1 background as homozygous lines. Homozygous VRC01^{gHL} B6 or VRC01^{gHL} B6.CD45.1 were crossed with homozygous B6 or B6.CD45.1 mice respectively, to generate VRC01^{gHL} heterozygous B6 or B6.CD45.1 mice. In some cases, VRC01^{gHL} heterozygous B6.CD45.1/CD45.2 mice were used and generated by crossing homozygous VRC01^{gHL} mice with homozygous B6.CD45.1 mice. C57BL/6J HYCAP1 and HYCAP3 mice carrying Env-specific knock-in TCRs (Lee et al., 2021a, 2021b) were crossed with B6.PL-*Thy1*^{a}/CyJ (CD90.1) background purchased from the Jackson Laboratory (JAX: 000406) to generate HYCAP1 or HYCAP3 CD90.1/CD90.2 mice, and eventually HYCAP1 or HYCAP3 CD90.1 homozygous mice.

METHOD DETAILS

Protein expression and purification

Trimer proteins were produced in FreeStyle™ 293F cells (Thermo Fisher Scientific, Cat# R79007) by transient transfection using 293Fectin (Thermo Fisher Scientific, Cat# 12347019) and pHL-sec plasmid containing mammalian codon-optimized constructs. Proteins were harvested from the supernatant after 7 days incubation at 37°C and purified by 2G12 antibody affinity chromatography using a HiTrap NHS-activated HP column (Cytiva, Cat# 17-0717-01) run on an ÄKTA Pure 25L HPLC (Cytiva, Cat# 29-0182-24). Trimers were polished by size exclusion chromatography (SEC) using a Superdex 200 16/600 size exclusion chromatography column (Cytiva, Cat# 28-9893-35) run on an ÄKTA Pure 25L HPLC. Final proteins were diluted in 1× TBS and stored at −80°C. For biotinylated probes, proteins were expressed with a his-tag and avi-tag, purified by Ni^{++} affinity chromatography followed by SEC, and biotinylated using a BirA biotin-protein ligase reaction kit (Avidity, Cat# BirA500) according to the manufacturer instructions. The avi-tagged MD39-GT3.1 knockout (KO) trimer (referred to in the text as MD39-GT3.1 KO4) was generated by adding mutations 280R, 365L, 368R, 371R, and 372L to MD39-GT3.1.

Paired HC and LC Fab variable region sequences from select VRC01^{gHL} affinity matured mAbs were gene synthesized and inserted into human IgG1 and IgK constant region expressing vectors pFUSEss-CHIg-hG1 (InvivoGen, Cat# pfusess-hchg1) and pFUSE2ss-CLIg-hK (InvivoGen, Cat# pfuse2ss-hclk) vectors. MAbs were expressed in 100 mL FreeStyle™ 293F cell cultures or 25 mL ExpiCHO™ cell cultures (Thermo Fisher Scientific, Cat# A29133). For 293F cell transfection, 50 μg of HC and 25 μg of LC plasmids were mixed with 225 μg polyethylenimine (PEI; 1:3 DNA:PEI ratio) in 5 mL of Opti-MEM™ reduced serum medium (Thermo Fisher Scientific, Cat# 31985070) for 30 min, then added to 293F cells. Supernatant was collected after 5–6 days. ExpiCHO™ cell cultures were transfected according to manufacturer instructions, using 8 μg HC and 7 μg LC plasmids. Supernatant was collected 8 days post transfection. Harvested supernatants were filtered through 0.45 or 0.25 μm membrane filters and batch bound overnight at 4°C to Protein A resin (Thermo Fisher Scientific, Cat# 20334) while on a rocker. Unbound supernatant was allowed to flowthrough, and the resin was washed with PBS until protein A280 reading of the flowthrough measured by a nanodrop reached background levels. Protein A bound IgG was eluted with 0.1 M Glycine pH 2.7. Eluted mAbs were buffer exchanged into 1× PBS and concentrated using a 50k MWCO concentrator (Millipore). RM19R Fab was expressed and harvested like IgG, but batch bound to CaptureSelect CH1-XL Affinity resin (Thermo Fisher Scientific, Cat# 1943462005) instead of Protein A. Resin was washed as described above, and captured Fabs were eluted with 50 mM NaOAc pH 4.0, buffer exchanged into 1× PBS, and concentrated using a 30k MWCO concentrator.

Size exclusion chromatography with multi-angle light scattering (SEC-MALS)

SEC-MALS was used to determine the molecular weight and uniformity of assembly of the trimeric proteins in solution. SEC-MALS was performed using a Wyatt Dawn Heleos-II MALS and Optilab T-rex refractive index detector coupled in-line with a Thermo-Dionex Ultimate 3000 HPLC system for size-exclusion chromatography. A Superdex 200 Increase 10/300 GL column (Cytvia, Cat# 28-9909-44) was equilibrated with 1× PBS with flowrate of 0.75 mg/mL at RT before injection of protein samples.

Differential scanning calorimetry (DSC)

DSC experiments were performed on a MicroCal VP-Capillary differential scanning calorimeter (Malvern Instruments). TBS buffer was used for baseline scans and protein samples were diluted to 0.25 mg/mL in TBS. The system was set to equilibrate at 20°C for 10 min and then heated up till 100°C at a scan rate of 90°C/h. Buffer correction, normalization, and baseline subtraction were applied during data analysis using Origin 7.0 software. The non-two-state model was used for data fitting.

Mice and immunizations

B cells from congenically marked VRC01^{gHL} mice (either CD45.1/CD45.2, CD45.1 homozygous, or CD45.2 homozygous background depending experiment and on mouse availability) were purified using the EasySep Mouse B Cell Isolation Kit (STEMCELL Technologies, Cat# 19854) according to manufacturer's instructions. 10^3 VRC01^{gHL} B cells were retro-orbitally transferred into the recipient mice (C57BL/6J or B6.CD45.1, depending on available donor cells) such that the precursor frequency would seed at 1 VRC01^{gHL} B cell in 10^6 splenic B cells (Abbott et al., 2018). For B and T cell co-transfers, 25×10^4 CD4 T cells from CD90.1$^+$ (CD90.1 homozygous or CD90.1/90.2) HYCAP1 or HYCAP3 mice were purified using the EasySep Mouse CD4$^+$ T Cell Isolation Kit (STEMCELL Technologies, Cat# 19852) (Lee et al., 2021a). In all experiments, RPMI 1640 (Corning, Cat# 10-040-CV) with 10% FBS was used as the transfer buffer.

Immunizations were carried out approximately 24 h post transfer. 20 μg of indicated immunogen diluted in PBS was mixed with 1 mg Alhydrogel alum (InvivoGen, Cat# vac-alu-250) in 200 μL total volume per dose. When Sigma Adjuvant (Sigma Aldrich, Cat# S6322-1VL) was used, 20 μg of trimer diluted in 1× PBS in a total volume of 100 μL was mixed with and equivalent volume of the adjuvant. The immunogen: adjuvant formulation was mixed and allowed to incubate at RT for 30 min. All mice were immunized intraperitoneally (i.p.) using a 25G needle. Mice were sacrificed at indicated time points following immunization and spleen and blood were harvested. Splenocytes were isolated from red blood cells using ACK lysing buffer (Thermo Fisher Scientific, Cat# A1049201), resuspended in FACS buffer (2.5% FBS in PBS) and enumerated. Cells were stained in the dark at 4°C with appropriate antibodies and live/dead stain in FACS buffer. Antibodies used in this study are listed in the key resources table. Cells were washed 2× with FACS buffer. For surface marker only panels, cells were fixed with BD Cytofix Fixation Buffer (BD Biosciences, Cat# 554655). For staining transcription factors, eBioscience FoxP3/Transcription Factor Staining Buffer Set (Thermo Fisher Scientific, Cat# 00-5523-00) was used to fix/permeabilize cells and stain with anti-transcription factor antibodies. Cells were kept at 4°C in the dark until acquisition then acquired on a LSRFortessa or FACSCelesta (BD Biosciences).

Identification of Env-specific CD4 T cells

Processed splenocytes from immunized mice were resuspended in D10 (DMEM, 10% FBS, 1× Pen Strep [Thermo Fisher Scientific, Cat# 15140122], 1× GlutaMAX^{TM} [Thermo Fisher Scientific, Cat# 35050061]) with 50 μM βMe, and seeded into sterile 96-well flat-bottom culture plates at 1 million cells/well in 100 μL (Corning, Cat# 3596), with a total of 4 million cells seeded per sample per condition. 100 μL of D10 + 1 μg/mL Alexa Fluor 647 conjugated anti-CD40L (clone: MR-1) with or without (unstimulated control) 4 μg/mL MD39-Env peptide pool was added to each well, for a final stimulation medium consisting of D10 + 50 μM βMe + 0.5 μg/mL Alexa Fluor 647 anti-CD40L with or without 2 μg/mL MD39-Env peptide pool. Purified anti-mouse CD40L antibody (Biolegend, Cat# 106517) was conjugated in advance with Alexa Fluor 647 using the Alexa Fluor 647 labeling kit (Thermo Fisher Scientific, Cat# A20173). The cells were *ex vivo* stimulated at 37°C for 6–8 h in a humidity and CO_2 controlled incubator. Following stimulation, cells were detached from plates by gently pipetting, and transferred to round bottom 96-well plates (Corning, Cat# 3798). Cells were washed twice then stained with antibody master mix. Cells were fixed with BD Cytofix and acquired on a BD FACSCelesta.

B cell receptor (BCR) sequencing

BCR sequences were obtained either by 10X Genomics Single Cell Immune Profiling (legacy version, 10X Genomics), or conventional single cell PCR. Splenocytes were stained for 10 min with Fc block at 4°C, then 30 min with Live/Dead (PI), dump (CD4/CD8/NK1.1/GR-1), B220, GL7, Fas, CD138, IgD, CD45.1, CD45.2, and appropriate TotalSeq-C Hashtag antibody (Biolegend) for each sample (see key resources table for antibody Cat#). Up to 15×10^4 total cells (all populations combined) were sorted on FACSAria-II or FACSAria-Fusion (BD Biosciences) sorters using an 85 μm nozzle. Congenically marked GL7$^+$/Fas$^+$/IgD$^-$/CD138$^-$ B_{GC} and VRC01^{gHL} B_{GC} cells were two-way bulk sorted into chilled 1.6 mL Eppendorf tubes containing 60 μL R10 (RPMI 1640, 10% FBS, 1× GlutaMAX^{TM}, 1× Pen Strep). At the end of the sort, fractions were combined into one Eppendorf tube. Cells were spun down for 5 min at 500 × g to remove excess R10, with approximately 30 μL of the supernatant remaining. Cells were gently resuspended and library construction was carried out following instructions provided by 10X Genomics (10X Genomics, CG000186 Rev A). cDNA quality and quantity were checked using the Agilent High Sensitivity D5000 ScreenTape Assay (Aligent, Cat# 5067–5592 and 5067–5593) and Qubit^{TM} dsDNA HS Assay Kit (Thermo Fisher Scientific, Cat# Q32851). Samples were sequenced on an a NovaSeq 6000 (Illumina) using the 150 × 150 bp configuration, aiming for 5000 read pairs per cell for the V(D)J cDNA library and 2000 read pairs for the Hashtag library.

Single cell sequencing was performed on sorted VRC01^{gHL} B_{GC} cells on day 36 post immunization. Cells were sorted using the same flow panel and gating strategy as the 10X Genomics samples but single cell sorted into fully skirted 96-well PCR plates with each well containing 15 μL of lysis buffer (10 mM Tris pH 8 [Sigma Aldrich, Cat# 648314], 500 U/mL RNase inhibitor [New England BioLabs, Cat# M0314L], 10 μg/mL Poly(A) [Sigma Aldrich, Cat# 10108626001]). cDNA synthesis, amplification, and Sanger sequencing of the VRC01 HCs and LCs was carried out as previously described (Abbott et al., 2018; von Boehmer et al., 2016).

BCR sequence analysis

Productive VRC01^{gHL} HC and LC nucleotide (NT) sequences were aligned to VRC01^{gH} and VRC01^{gL} variable region reference sequences using the Clustal Omega package provided within the Unipro UGENE software (Okonechnikov et al., 2012). Aligned NT and amino acid (AA) sequences were compared to the unmutated reference sequences to check for mutations. VRC01-class mutations were tabulated using the code published by Briney et al. (Briney et al., 2016) where VRC01-class mutations are identified as

mutations in the query BCR HC V-gene region that are identical to mutations observed in the reference set of VRC01-class antibodies (VRC01, PGV04, VRC-CH31, 3BNC60, 12A12, PGV20). Our code was updated to include PCIN63-71I as an additional reference VRC01-class bnAb sequence (Huang et al., 2020). ARMADiLLO (Wiehe et al., 2018) analysis was performed on V-gene regions of HC and LC. LCs with the L-CDR1 deletion were not included in the analysis because mutation probability for deletions is not calculated in the algorithm.

For analysis of 10× Genomics platform derived sequences, Hashtag counts and assembled BCR sequences were generated by the Cell Ranger software package v3.0 (10X Genomics). The mouse VDJ reference file was modified to include human IGHV1-2*02, IGHJ*01, IGHD3-16*01, IGKV3-11*01, IGKJ2*01/*02/*03/*04 reference genes to allow Cell Ranger VDJ to assemble the human VDJ derived VRC01^{gHL} sequences. Some of the cell barcodes were associated with more than one HC or LC sequence likely due to doublets and triplets being captured per droplet. As such, sequences were deconvoluted into their respective sample groups according to the following criteria. For endogenous BCR sequences, each cell barcode was assigned a Hashtag if the count exceeded 1000. If these cell barcodes were also associated with at least 100 counts of a second Hashtag, sequences associated with these cell barcodes were excluded from analysis. For paired VDJ sequence analyses, cells with more than one HC-LC pair were excluded due to pairing ambiguity. For IGH isotype analysis, all HC isotypes within each demultiplexed Hashtag group were analyzed, including HCs not paired with an LC and dual HC sequences derived from a single cell barcode.

VRC01^{gHL} HC sequences were analyzed if they did not have high overlapping Hashtag counts (>100) with a second VRC01^{gHL} Hashtag group, and likewise for VRC01^{gHL} LC sequences. Among early time point sequences (day 10 and less so for day 14) several VRC01^{gHL} LC sequence reads did not have spliced introns in the leader sequence region. This may be related to our previous observation that naïve VRC01^{gHL} B cells had lower surface BCR expression than endogenous C57BL/6J B cells, and showed dual LC expression with an endogenous lambda chain (Abbott et al., 2018). These phenotypes were resolved over time following eOD-GT5 60mer immunization (Abbott et al., 2018). Unspliced leader sequence resulted in Cell Ranger flagging the sequences as being unproductive. However, because the VJ region of those LC sequence were productive (no frameshift or stop codon mutations), these sequences were included in the analysis of VRC01^{gHL} LC sequences.

The Python script used to consolidate Hashtag counts and VDJ annotation data can be found from https://github.com/jvxtaposed/Filter-Cellranger-VDJ (Lee et al., 2021b).

Biolayer interferometry (BLI)

MAbs from MD39-GT3.1 immunized mice were digested into Fabs using the Pierce Fab Preparation Kit (Thermo Fisher Scientific, Cat# 44985) in accordance with the supplied instructions. Digested Fabs were buffer exchanged in to 1× PBS using a 10K MWCO concentrator (Millipore). Ligand and analyte samples for BLI kinetic measurements on ForteBio Octet RED384 (Sartorius) were prepared by appropriate dilution into 1× Kinetics buffer (0.02% v/v Tween 20, 0.1% w/v BSA in 1× PBS). Fabs were titrated down 2-fold from ~53 nM to 6.6~3.3 nM (2.5 μg/mL to 0.312~0.156 μg/mL). The glVRC01 Fab was titrated 2-fold from ~1 μM down to 31 nM. Biotinylated MD39-GT3.1 or MD39-N276D trimer diluted to 2–2.5 μg/mL in 1× Kinetics buffer were captured onto the streptavidin (SA) capture BLI sensor (Sartorius) for 200 s, then returned to baseline for 120 s. The trimer loaded sensors were dipped into dilutions of Fabs for 300 s for the association step, then dissociated for 300 s. Binding curves were fitted using a 1:1 binding model within the Data Analysis HT software v11.1 (Sartorius).

Surface plasmon resonance (SPR)

Kinetics and affinity of antibody-antigen interactions were measured on ProteOn XPR36 (Bio-Rad Laboratories) using GLC Sensor Chip (Bio-Rad Laboratories) and 1× HBS-EP + pH 7.4 running buffer (20× stock, Teknova, Cat# H8022) supplemented with BSA at 1 mg/mL. We followed Human Antibody Capture Kit instructions (Cytvia, Cat# BR-1008-39) to prepare the chip surface for ligand capture. Approximately 5700 RU of anti-human Fc IgG capture antibody was amine coupled in all flow cells of the GLC Chip. PGT121 IgG (Walker et al., 2011) was captured with anti-human Fc IgG, then trimer ligands were captured with PGT121 IgG, after which Fab analytes were flowed to assess binding. PGT121 ligand concentration was 2 μg/mL, and trimer concentration was 10 μg/mL. Regeneration was done with 0.85% phosphoric acid, 15 s contact time, 4 injections. For double referencing we used a blank channel and blank injection. For eOD-GT5 binding, monomeric eOD-GT5 was captured on to the sensor, and Fabs were tested from a top concentration of 22.4 μM and five successive 4-fold dilutions. Raw sensorgrams were analyzed using ProteOn Manager software (Bio-Rad Laboratories) with Langmuir model. Analyte concentrations were measured on NanoDrop 2000c Spectrophotometer using Absorption signal at 280 nm.

Enzyme-linked immuno-sorbent assay (ELISA)

ELISA was performed to analyze the binding of bnAbs and non-nAbs binding to MD39 and MD39-GT3.1. Either capture antibody (RM19R Fab) or HIV trimers were directly coated onto high-binding 96-well half-area plates (Corning, Cat# 3690) at 2 μg/mL in 25 μL 1× PBS (Thermo Fisher Scientific, Cat# 10010023) per well and incubated overnight at 4°C. Plates were washed 3× with PBS containing 0.2% v/v tween (PBST) (Tween 20; Sigma Aldrich, Cat# P1379-1L) in a 405 TS Washer (BioTek Instruments) and blocked with PBST containing 5% w/v skim milk (BD Difco™ Skim Milk; BD Life Sciences, Cat# 232100) and 1% v/v FBS (Thermo Fisher Scientific, Cat #16000044) for 1 h at RT. Plates were washed 1× and 25 μL of dilution series of primary mAbs in PBST +1% FBS were added for 1 h at 37°C. Plates were washed 3× and mAb binding was detected by adding 25 μL of anti-Human IgG, Fcγ fragment specific antibody

(Jackson ImmunoResearch, Cat# 109-035-098) at 1:5000 dilution in PBST + 1% FBS. After 1 h incubation at RT, plates were washed 3× and 25 μL TMB Chromogen Solution (Thermo Fisher Scientific, Cat# 002023) substrate was added. After 6 min, 25 μL 0.5 M H_2SO_4 was added to stop the reaction. Absorption was read at 450 and 570 nm on a VERSA max plate reader (Molecular Devices).

For *Galanthus nivalis* lectin (GNL) capture serum ELISAs, high-binding 96-well half-area plates were coated overnight at 4°C with 30 μL 4.5 μg/mL of GNL (Sigma Aldrich, Cat# L8275-5 mg) in 1× PBS. Plates were washed 5× with wash buffer (1× PBS, 0.05% v/v Tween 20) and incubated with 30 μL of BG505 trimer at 1 μg/mL for 2 h at 37°C. Trimer bound plates were washed 5× with wash buffer, and blocked with 150 μL of blocking buffer (1× PBS, 3% w/v BSA) for 1 h at RT. 50 μL of sera serially diluted in dilution buffer (1× PBS, 1% w/v BSA) were added to the plates and incubated for 1 h at RT. For cross-competition ELISA, plates were first incubated with RM19R Fab (Cirelli et al., 2019; Cottrell et al., 2020) for 1.5 h at RT. Plates were then washed 5× in wash buffer, followed by the addition of serially diluted samples as indicated above. For no competition controls run in parallel, dilution buffer was added in place of RM19R Fab. Plates were washed 5× in wash buffer. 50 μL anti-mouse IgG-HRP (Jackson ImmunoResearch, Cat# 115-035-166) diluted 1:10,000 in dilution buffer was added to all wells for 1 h at RT. Plates were washed 5× and detected with 50 μL 1-Step Ultra TMB-ELISA Substrate Solution (Thermo Fisher Scientific, Cat# 34028) for 5 min. The reaction was stopped with 50 μL 2N H_2SO_4 (Ricca Chemical, Cat# 8310–32), and read on a Perkin Elmer EnVision Microplate Reader at 450 nm.

For ELISAs where the antigen was directly captured, plates were coated overnight at 4°C with 30 μL of the antigen of interest diluted to 2 μg/mL in 1× PBS. Plates were washed 5× with wash buffer, blocked with blocking buffer, and serum or mAbs diluted in dilution buffer were added to plates for 1 h at RT as described above. Plates were washed 5×, and the secondary Ab conjugated with HRP diluted in dilution buffer was added to each well. The secondary Ab was either anti-mouse IgG-HRP (1:10,000 dilution; Jackson ImmunoResearch, Cat# 115-035-166) or anti-human IgG-HRP (1:5,000 dilution; Jackson ImmunoResearch, Cat# 709-035-149) depending on the experiment. Signal detection was performed as described above.

For SA capture ELISAs, 96-well half-area plates were coated overnight with 50 μL of 2.5 μg/mL SA (Jackson ImmunoResearch, Cat# 016-000-084) in 1× PBS at 4°C. Plates were washed 5× in wash buffer, after which 30 μL of biotinylated MD39-N276D trimers (2.5 μg/mL in PBS) were added for 2 h at 37°C. Plates were washed 5× with wash buffer, then blocked for 1 h with blocking buffer at RT. Primary and secondary antibody binding steps were carried out as described for direct antigen capture ELISAs. Signal detection was performed as stated above. In some experiments, alkaline phosphatase (AP) conjugated anti-human IgG antibody (1:250 dilution in dilution buffer, Jackson ImmunoResearch, Cat# 109-055-003) was used as a secondary. In those cases, signal was detected with phosphatase substrate (Sigma Aldrich, Cat# S0942). Reaction was stopped with 2N NaOH, and read on a Perkin Elmer EnVision Microplate Reader at 405 nm. EC_{50} was determined by normalizing signal to maximum signal detected by mature VRC01 binding, then by fitting a nonlinear regression dose response curve. Endpoint titers were interpolated based on an asymmetrical logistic-dose response curve. AUC, EC_{50} and Endpoint titers were all calculated in Prism 8 (GraphPad).

Neutralization assay

Mab neutralizing activity was assessed using single round replication viruses in TZM-bl target cells as described previously (Landais et al., 2016). The parent virus of the pseudoviruses used in our panel are described elsewhere (Hoffenberg et al., 2013; Seaman et al., 2010; Simek et al., 2009). Pseudoviruses incorporating single AA mutations were generated by Quickchange mutagenesis (Stratagene). Wild-type (WT) and mutant pseudoviruses were generated by co-transfection of HEK293 T or HEK293S (*N*-acetylglucosaminyltransferase-I deficient, GnTI−/−) cells using the FuGENE6 transfection reagent (Promega, Cat# E2692) with an Env-expressing plasmid and an Env-deficient genomic backbone plasmid (pSG3Δenv). Pseudoviruses were harvested 72 h post transfection for use in neutralization assays. Target TZM-bl cells in D10 were seeded onto 96-well half area solid white flat bottom plates (Corning, Cat# 3688). Individual pseudovirus stocks were mixed with mAbs serially diluted in D10, and pre-incubated 1 h at 37°C with before addition to target cells in presence of 5 μg/mL DEAE-dextran (Sigma Aldrich, Cat# 93556-1G). Infectivity was assessed after 72 h using Luciferase Assay readout reagent kits (Promega, Cat# E4550) as per manufacturer instructions. Signal was detected by a VersaMax Microplate Reader (Molecular Devices). All experiments were carried out in duplicate and repeated at least twice. Neutralization was calculated as the percentage loss of infectivity relative to pseudovirus only control wells. To determine neutralization IC_{50} (concentration yielding 50% neutralization) values, the dose-response curves were fitted using constrained (Top = 0, Bottom = 100) nonlinear regression in Prism 8 (GraphPad).

QUANTIFICATION AND STATISTICAL ANALYSIS

Statistical analyses were performed using GraphPad Prism v8. Bars plotted in linear scale represent the mean and standard deviation (SD). Graphs plotted in log scale represent the geometric mean and geometric standard deviation. Statistical significance between indicated groups were queried by an unpaired, two-tailed Students-t test at a confidence level of 95%. p values are as follows: ns > 0.05, *p ≤0.05, **p ≤0.01, ***p ≤0.001, ****p ≤0.0001. Prism v8 was used to the analyses of all ELISA data; area under curve (AUC) was determined using the Area under curve function, EC_{50} was calculated using the log(agonist) vs. normalized response – variable slope function, and endpoint titers were interpolated using the Asymmetric Sigmoidal, 5PL, X is log(concentration) curve. Neutralization IC_{50} values were calculated via a constrained nonlinear regression fit (Top = 0, Bottom = 100) using the One site – Fit logIC_{50} in Prism v8. N = number of independent experiments performed, n = number of samples per given experiment.

Cell Reports, Volume *38*

Supplemental information

Highly mutated antibodies capable of neutralizing N276 glycan-deficient HIV after a single immunization with an Env trimer

Jeong Hyun Lee, Catherine Nakao, Michael Appel, Amber Le, Elise Landais, Oleksandr Kalyuzhniy, Xiaozhen Hu, Alessia Liguori, Tina-Marie Mullen, Bettina Groschel, Robert K. Abbott, Devin Sok, William R. Schief, and Shane Crotty

A

```
BG505 SOSIP           AENLWVTVYYGVPVWKDAETTLFCASDAKAYETEKHNVWATHACVPTDPNPQEIHLENVTEEFNMWKNNMVEQMHTDIISLWDQSLK
BG505 SOSIP-GT3       AENLWVTVYYGVPVWKDAETTLFCASDAKAYETEKHNVWATHACVPTDPNPQEIHLENVTEEFNMWKNNMVEQMHTDIISLWDQSLK
BG505 MD39-GT3.1      AENLWVTVYYGVPVWKDAETTLFCASDAKAYETEKHNVWATHACVPTDPNPQEIHLENVTEEFNMWKNNMVEQMHEDIISLWDQSLK
BG505 SOSIPv4.1-GT1   AENLWVTVYYGVPVWKDAETTLFCASDAKAYETKKHNVWATHACVPTDPNPQEIHLENVTEEFNMWKNNMVEQMHTDIISLWDQSLK

BG505 SOSIP           PCVKLTPLCVTLQCTNVTNNITDDMRGELKNCSFNMTTELRDKKQKVYSLFYRLDVVQINENQGNRSNNSNKEYRLINCNTSAITQA
BG505 SOSIP-GT3       PCVKLTPLCVTLQCTNVTNNITDDMRGELKNCSFNMTTELRDKKQKVYSLFYRLDVVQINENQGNRSNNSNKEYRLINCNTSAITQA
BG505 MD39-GT3.1      PCVKLTPLCVTLQCTNVTNNITDDMRGELKNCSFNMTTELRDKKQKVYSLFYRLDVVQINENQGNRSNNSNKEYRLINCNTSAITQA
BG505 SOSIPv4.1-GT1   PCVKLTPLCVTLQCTNVTNNITDDMRGELKNCSFNMTTELRDKRQKVHALFYKLDIVPINENQNT-------SYRLINCNTAAITQA

BG505 SOSIP           CPKVSFEPIPIHYCAPAGFAILKCKDKKFNGTGPCPSVSTVQCTHGIKPVVSTQLLLNGSLAEEEVMIRSENITNNAKNILVQFNTP
BG505 SOSIP-GT3       CPKVSFEPIPIHYCAPAGFAILKCKDKKFNGTGPCPSVSTVQCTHGIKPVVSTQLLLNGSLAEEEVMIRSEDFRNNAKNILVQFNTP
BG505 MD39-GT3.1      CPKVSFEPIPIHYCAPAGFAILKCKDKKFNGTGPCPSVSTVQCTHGIKPVVSTQLLLNGSLAEEEVIIRSEDFRNNAKNILVQLNTP
BG505 SOSIPv4.1-GT1   CPKVSFEPIPIHYCAPAGFAILKCKDKKFNGTGPCPSVSTVQCTHGIKPVVSTQLLLNGSLAEEEVMIRSEDIRNNAKNILVQFNTP

BG505 SOSIP           VQINCTRPNNNTRKSIRIGPGQAFYATGDIIGDIRQAHCNVSKATWNETLGKVVKQLRKHFGNNTIIRFANSSGGDLEVTTHSFNCG
BG505 SOSIP-GT3       VQINCTRPNNNTRKSIRIGPGQAFYATGDIIGDIRQAHCNVSKATWNETLGKVVKQLRKHYGNDTTIRFAPSSGGDLEVMNHSFNCG
BG505 MD39-GT3.1      VQINCTRPNNNTVKSIRIGPGQAFYYTGDIIGDIRQAHCNVSKATWNETLGKVVKQLRKHYGNDTTIRFAPSSGGDLEVMNHSFNCG
BG505 SOSIPv4.1-GT1   VQINCTRPNNNTRKSIRIGPGQWFYATGDIIGDIRQAHCNVSKATWNETLGKVVKQLRKHFGNNTIIRFANSSGGDLEVTTHSFNCG

BG505 SOSIP           GEFFYCNTSGLFNSTWISNTSVQGSNSTGSNDSITLPCRIKQIINMWQRIGQAMYAPPIQGVIRCVSNITGLILTRDGGSTNSTTET
BG505 SOSIP-GT3       GEFFYCNTSGLFNSTWISNTSVQGSNSTGSNDSITLPCRIKQIINMWQRIGQAMYAPPIQGVIRCVSNITGLILVRDGGYTNSNTET
BG505 MD39-GT3.1      GEFFYCNTSGLFNSTWISNTSVQGSNSTGSNDSITLPCRIKQIINMWQRIGQAMYAPPIQGVIRCVSNITGLILVRDGGYTNSNTET
BG505 SOSIPv4.1-GT1   GEFFYCDTSGLFNSTWISNTSVQGSNSTGSNDSITLPCRIKQIINMWQRIGQAMYAPPIQGVIRCVSNITGLILTRDGGSTDSTTET

BG505 SOSIP           FRPGGGDMRDNWRSELYKYKVVKIEPLGVAPTRCKRRVVGRRRRRRAVGIGAVFLGFLGAAGSTMGAASMTLTVQARNLLSGIVQQQ
BG505 SOSIP-GT3       FRPGGGDMRDNWRSELYKYKVVKIEPLGVAPTRCKRRVVGRRRRRRAVGIGAVFLGFLGAAGSTMGAASMTLTVQARNLLSGIVQQQ
BG505 MD39-GT3.1      FRPGGGDMRDNWRSELYKYKVVKIEPLGVAPTRCKRRVVGRRRRRRAVGIGAVSLGFLGAAGSTMGAASMTLTVQARNLLSGIVQQQ
BG505 SOSIPv4.1-GT1   FRPSGGDMRDNWRSELYKYKVVKIEPLGVAPTRCKRRVVGRRRRRRAVGIGAVFLGFLGAAGSTMGAASMTLTVQARNLLSGIVQQQ

BG505 SOSIP           SNLLRAPEAQQHLLKLTVWGIKQLQARVLAVERYLRDQQLLGIWGCSGKLICCTNVPWNSSWSNRNLSEIWDNMTWLQWDKEISNYT
BG505 SOSIP-GT3       SNLLRAPEAQQHLLKLTVWGIKQLQARVLAVERYLRDQQLLGIWGCSGKLICCTNVPWNSSWSNRNLSEIWDNMTWLQWDKEISNYT
BG505 MD39-GT3.1      SNLLRAPEPQQHLLKDTHWGIKQLQARVLAVEHYLRDQQLLGIWGCSGKLICCTNVPWNSSWSNRNLSEIWDNMTWLQWDKEISNYT
BG505 SOSIPv4.1-GT1   SNLLRAPEAQQHLLKLTVWGIKQLQARVLAVERYLRDQQLLGIWGCSGKLICCTNVPWNSSWSNRNLSEIWDNMTWLQWDKEISNYT

BG505 SOSIP           QIIYGLLEESQNQQEKNEQDLLALD
BG505 SOSIP-GT3       QIIYGLLEESQNQQEKNEQDLLALD
BG505 MD39-GT3.1      QIIYGLLEESQNQQEKNEQDLLALD
BG505 SOSIPv4.1-GT1   QIIYGLLEESQNQQEKNEQDLLALD
```

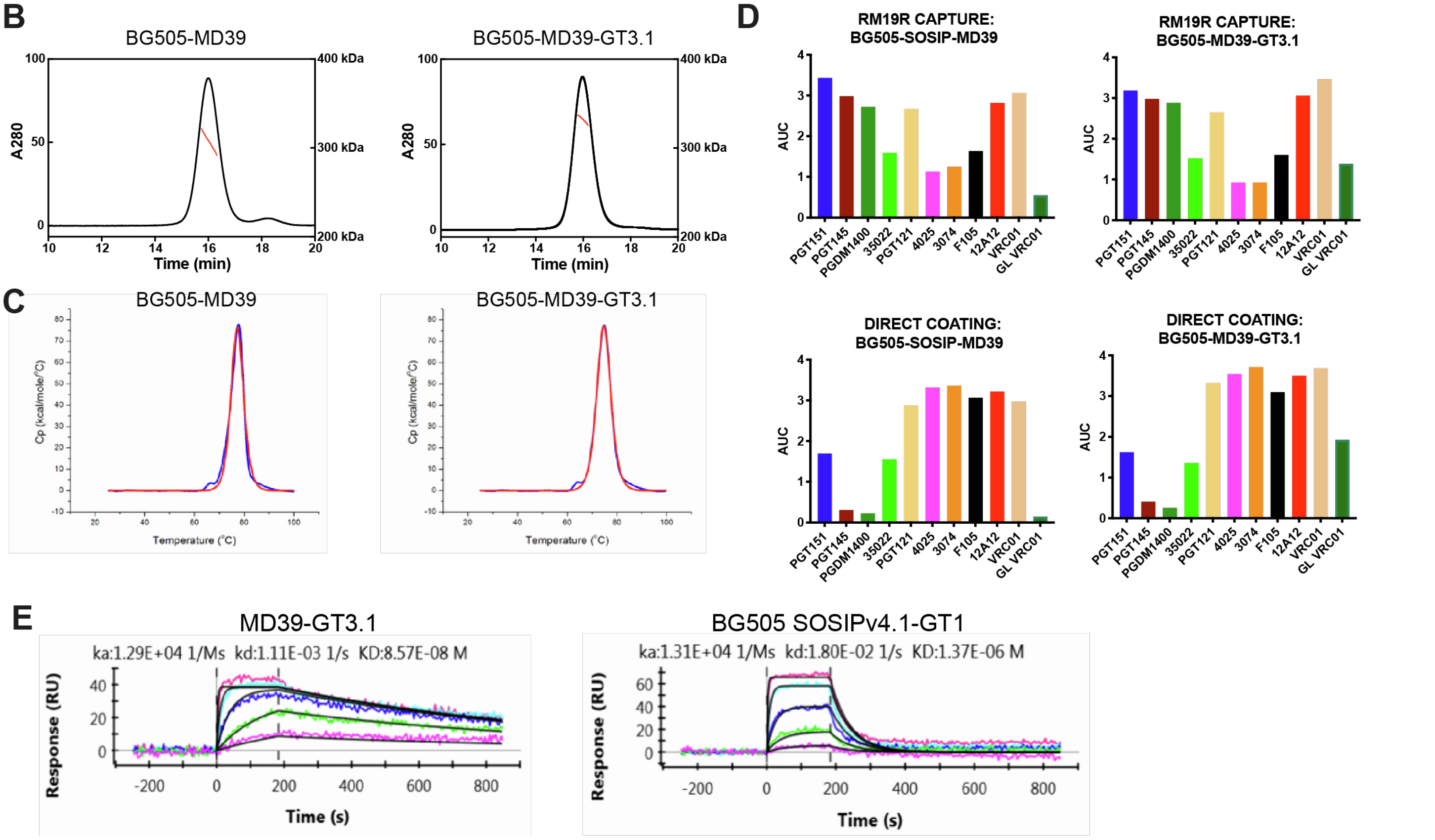

Figure S1. Characterization of the MD39-GT3.1 trimer immunogen and related immunogens, related to Figures 1 and 2.

(A) The sequence of BG505 SOSIP trimer and BG505 SOSIP-based CD4bs GT trimer constructs. Modifications from the parent BG505 SOSIP sequence is shown in red. The furin cleavage site between gp120 and gp41 is shown in bold font. The 15mer epitope of HYCAP Tg CD4 T cells is underlined.
(B) Size exclusion chromatography-multiangle light scattering (SEC-MALS) traces for BG505 MD39 and BG505 MD39-GT3.1. The left y-axis is normalized A280 and right y-axis is the molecular weight of protein and glycan. The MW assessed by protein-conjugate analysis is shown as the red line inside the peak. Theoretical protein MW is 214 kDa in both cases.
(C) Thermal stability of BG505 MD39 and BG505 MD39-GT3.1 measured by differential scanning calorimetry (DSC). The raw data are shown as blue solid line, and the fit is shown as red line. Melting temperature (Tm) values from the fit are shown.
(D) Antigenic profiles measured by ELISA for BG505 MD39 and BG505 MD39-GT3.1, with trimers either captured by the base binding mAb RM19R (Cottrell et al., 2020) in order to preserve trimer quaternary structure or coated directly onto the ELISA plate so as to disrupt trimer quaternary structure.
(E) SPR kinetic analysis of glVRC01 Fab binding to MD39-GT3.1 trimer (left) and BG505 SOSIPv4.1-GT1 (right) captured on sensor by PGT121. The glVRC01 Fab analyte was tested at a top concentration of 30.347 μM and four successive 4-fold dilutions. The dissociation constant from this kinetic fit was 86 nM and1.4 μM, respectively.

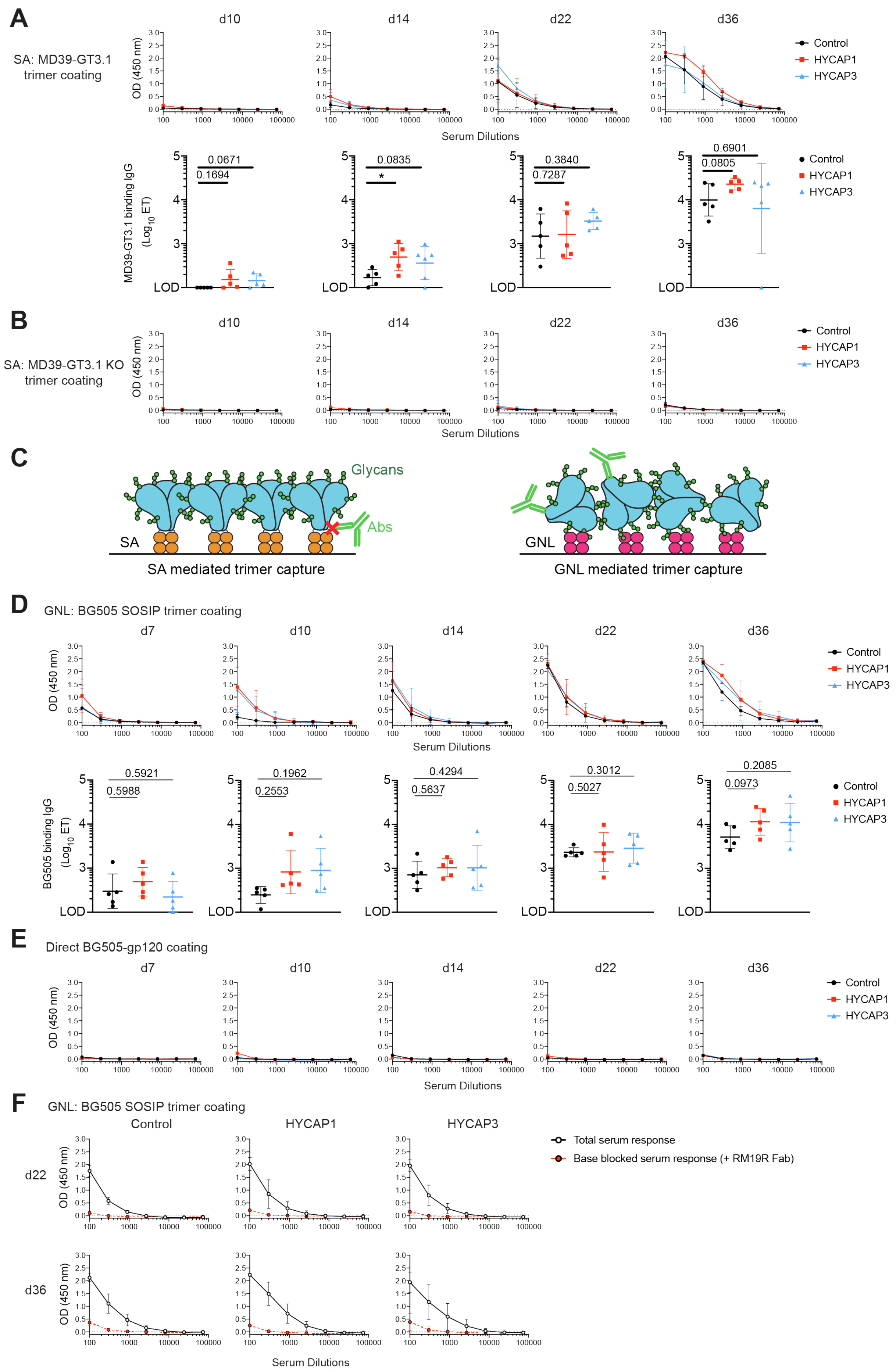

Figure S2. Serum IgG responses from MD39-GT3.1 immunized mice, related to Figure 2.

(A) MD39-GT3.1 trimer-binding serum IgG measured by ELISA and endpoint titers (ET). Biotinylated MD39-GT3.1 trimers were captured on to SA coated plates.
(B) As in (A), but serum IgG binding to the MD39-GT3.1 KO trimer.
(C) Comparison of SA-biotinylated trimer capture and GNL-trimer capture. SA-based trimer capture results in C-terminus oriented binding of the trimer on to ELISA plates, sterically occluding the trimer base epitope. GNL binds mannose residues and captures Env trimers in random orientations.
(D) BG505 SOSIP trimer-binding serum IgG measured by ELISA and ET. Plates were initially coated with GNL, and trimer was captured onto the GNL coated plates. P-values calculated by two-tailed Student's t-tests.
(E) IgG serum ELISA to detect monomeric BG505 gp120. Recombinant BG505 gp120 was directly coated onto ELISA plates.
(F) As in (D) for d22 and d36 (black lines), but with the respective trimer base blocking ELISA performed in parallel (red lines). To block the base epitope, RM19R Fab was added to the trimers prior to the addition of mouse serum.

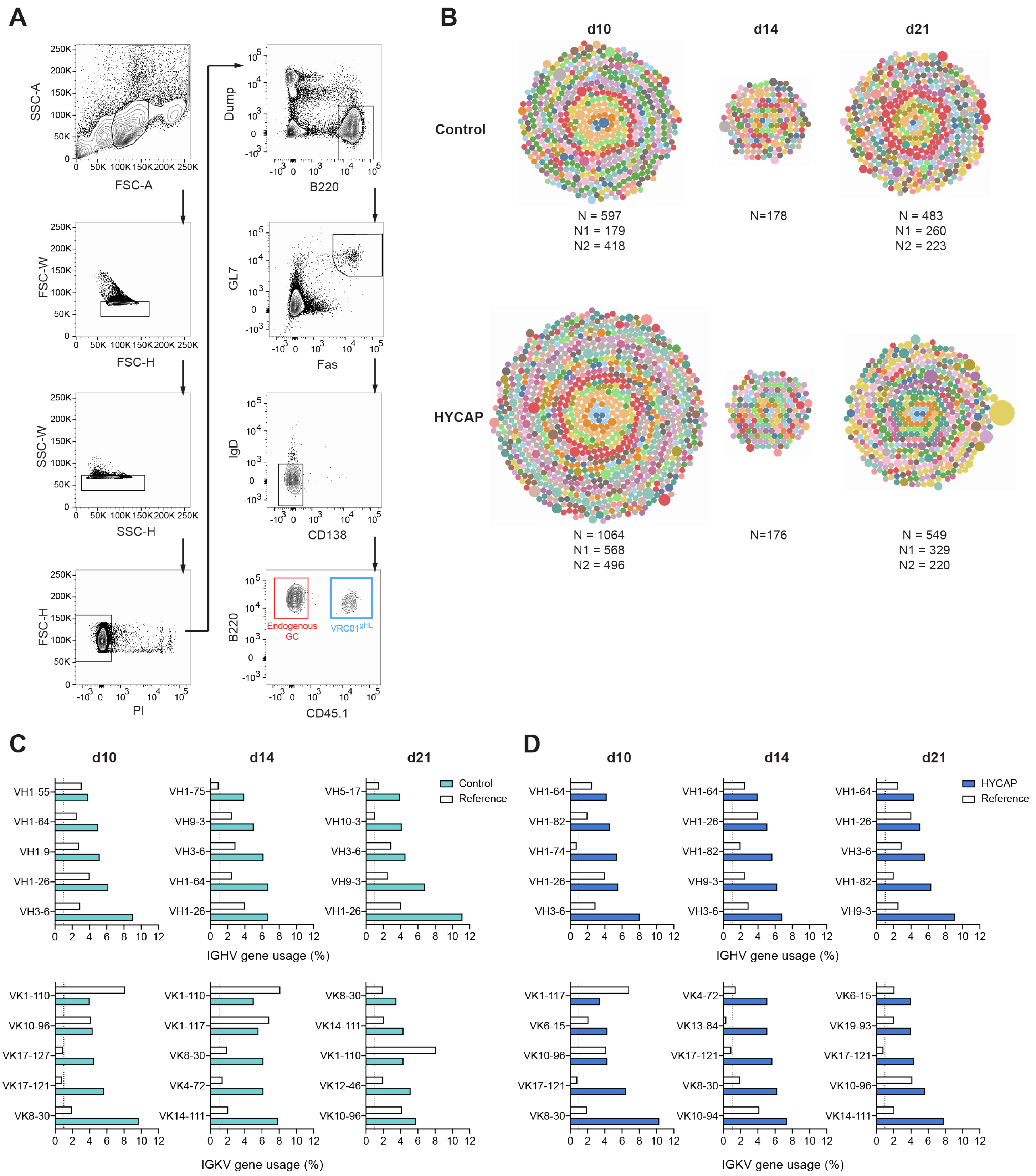

Figure S3. Analysis of endogenous B$_{GC}$ BCR sequences in MD39-GT3.1 trimer immunized mice, related to Figure 2.

(A) Endogenous B$_{GC}$ cell and VRC01^{gHL} B$_{GC}$ cell sort strategy for sequencing. Dump: CD4, CD8a, NK1.1, Gr-1.

(B) Diversity of endogenous B$_{GC}$ cells. Each circle denotes a unique clonotype, defined by paired HC VDJ-LC VJ genes. The size of each circle is directly proportional to the number of clones observed for that clonotype. Circles are colored by IGHV-genes although the colors are not mutually exclusive due to the large number of IGHV genes observed. Distinct

clones using the same IGHV-genes are proximally clustered. N = total number of paired sequences obtained from two independent experiments; N1 = sequences from first experiment, pooled from 4 mice each for both control and HYCAP3 groups; N2 = sequences from a second experiment, pooled from 5 mice in the control group, pooled from 10 mice in the HYCAP group (5 mice from HYCAP1 and HYCAP3 groups each). B cells from d14 were only sequenced in the second experiment.
(C) The top 5 most used IGHV (VH, upper) and IGKV (VK, lower) genes among HC-LC paired endogenous B_{GC} cells after 10, 14 or 21 days post immunization in the control group. The reference V-gene usage frequencies were derived from V-gene usage observed unimmunized C57BL/6J splenic B cells (Rettig et al., 2018). Dotted line indicates 1%.
(D) As in (C) but analysis of sequences from mice that received HYCAP1 and HYCAP3 CD4 T cells by adoptive transfer.

A

	Control	N1	N2	N1+N2	HYCAP1	N1	N2	N1+N2	HYCAP3	N1	N2	N1+N2	Total
d10	HC	5	89	94	HC	0	221	221	HC	312	242	554	869
	LC	3	49	52	LC	0	142	142	LC	193	145	338	532
d14	HC	0	117	117	HC	0	201	201	HC	0	215	215	533
	LC	0	100	100	LC	0	146	146	LC	0	165	165	411
d21	HC	43	158	201	HC	0	283	283	HC	97	261	358	842
	LC	46	129	175	LC	0	214	214	LC	93	209	302	691
d36	HC	0	133	133	HC	0	131	131	HC	0	117	117	381
	LC	0	113	113	LC	0	96	96	LC	0	83	83	292

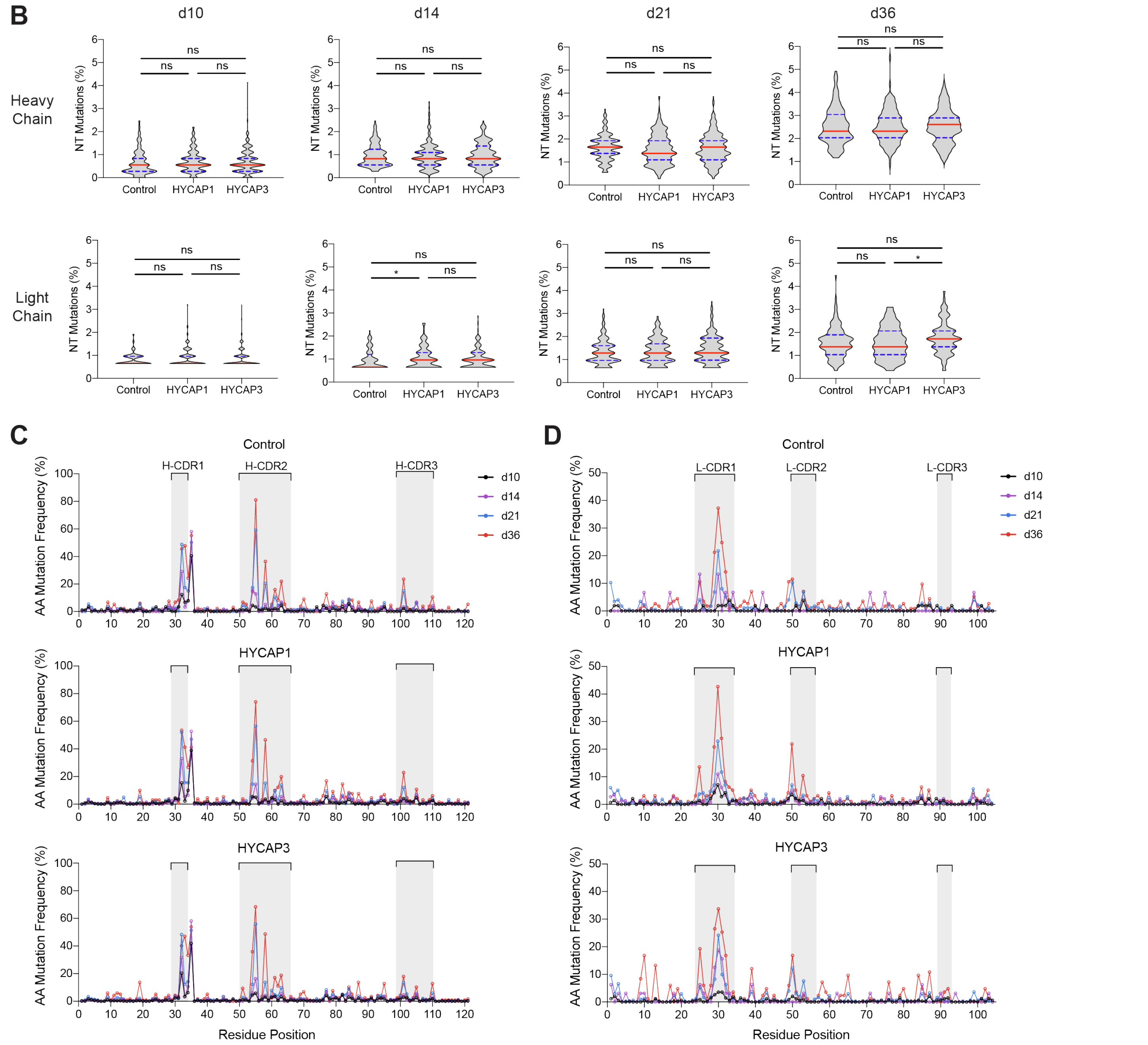

Figure S4. Sequencing of VRC01^{gHL} cells, related to Figure 3.

(A) Number of B_{GC} VRC01^{gHL} HC and LC sequences analyzed. N1 and N2 indicate two independent experiments. D10, 14, 21 B cells were sequenced using the 10X Genomics platform and pooled from 4 mice per group for each experiment. D36 sequences were obtained by Sanger sequencing from 4 mice within each transfer group.

(B) Violin plots of % nucleotide (NT) mutations for the data shown in Figure 2A. The denominator for d10, 14, 21 sequences is 363 NT residues for HC and 312 residues for LC. The denominator for d36 sequences is 345 NT residues for HC and 290 residues for LC.

(C) Per residue HC AA mutations in the three experimental groups.
(D) Per residue LC AA mutations in the three experimental groups.

			M1		M2		M3		M4		M5		M6		M7		M8		M9	
Virus Strain	Tier	Clade	T	S	T	S	T	S	T	S	T	S	T	S	T	S	T	S	T	S
191084 B7-19_N276A	2	A	0.02631	0.00295	0.02851	0.00276	1.07981	0.05221	0.02356	0.00146	26.43695	0.11549	9.25587	0.06637	1.24439	0.00542	0.07767	0.00225	0.23643	0.00289
BG505_N276A	2	A	3.87232	NT	21.55737	NT	17.87130	NT	8.80939	NT	NN	NT	66.33857	NT	76.17481	NT	0.53851	NT	79.94976	NT
BG505_N276D	2	A	3.06971	NT	40.76442	NT	3.46174	NT	0.49062	NT	95.17247	NT	17.85259	NT	3.84156	NT	0.05780	NT	1.14787	NT
REJO4541.67_N276A	2	B	3.79945	0.01394	44.31766	0.10066	11.37000	0.39231	8.65472	0.01772	NN	NN	NN	22.88683	NN	NN	NN	38.42655	NN	93.06217
1012_11_TC21_3257_N276A	1B	B	0.12607	0.02426	0.44165	0.01760	6.86379	0.22708	7.33115	0.09931	NN	95.86216	NN	37.24879	15.54891	0.45328	5.41503	0.03266	21.41162	0.11809
HIV-001428-2.42_N276A	2	C	0.01680	0.00082	7.89591	0.01182	2.64889	0.03082	0.60076	0.00297	NN	15.01911	21.68769	0.15419	19.81655	0.14983	0.04261	0.00057	1.20000	0.00210
3301.v1.c24_N276A		AC	NN	0.06797	NN	0.09463	NN	0.46500	NN	0.09445	NN	1.07127	NN	6.92608	NN	2.79699	NN	0.03651	NN	0.02381
6041.v3.c23_N276A		AC	NN	0.62301	NN	NN	NN	NN	NN	5.48987	NN	NN	NN	NN	NN	NN	29.68087	0.11642	NN	5.32711
92TH021_N276A	2	AE	NN	0.13069	NN	NN	NN	NN	NN	NN	NN	NN	NN	NN	NN	NN	NN	0.49901	NN	NN
191084 B7-19_N276D	2	A	0.07461	0.00304	0.04816	0.00324	0.25399	0.00722	0.02596	0.00212	0.95622	0.01078	0.11903	0.00379	0.04188	0.00283	0.02837	0.00215	0.04985	0.00337
SC422661.8_N276D	2	B	1.72409	NT	3.75924	NT	8.09772	NT	0.32041	NT	NN	NT	NN	NT	24.88707	NT	16.12163	NT	78.30517	NT
HIV-001428-2.42_N276D	2	C	0.02598	0.00085	15.10990	0.01596	1.14572	0.00733	0.16154	0.00250	NN	2.77310	34.96976	0.14733	37.50414	0.03775	0.01913	0.00037	0.23609	0.00125
HIV-0815.v3.c3_N276D	2	ACD	4.12250	NT	NN	NT	NN	NT	2.30058	NT	50.56341	NT	NN	NT	NN	NT	0.73764	NT	NN	NT
MLV			NN	NN	NN	NN	NN	NN	NN	NN	NN	NN	NN	NN	NN	NN	NN	NN	NN	NN

			M10		M11		M12		M13		M14		M15		E7		E11		glVRC01		VRC01	
Virus Strain	Tier	Clade	T	S	T	S	T	S	T	S	T	S	T	S	T	S	T	S	T	S	T	S
191084 B7-19_N276A	2	A	5.50763	0.02893	9.50712	0.03078	0.05328	0.00272	0.00746	0.00049	NN	79.73813	NN	28.77998	2.77038	0.02073	0.02364	0.00149	NN	NN	0.03100	0.00092
BG505_N276A	2	A	24.89845	NT	NN	NT	NN	NT	1.00591	NT	NN	ND	NN	ND	NN	NT	NN	NT	NN	ND	0.01300	NT
BG505_N276D	2	A	0.10831	NT	0.64819	NT	3.72484	NT	0.00850	NT	NN	ND	NN	ND	30.75698	NT	15.40579	NT	NN	ND	0.01100	NT
REJO4541.67_N276A	2	B	NN	63.44921	NN	NN	NN	0.39616	0.00805	0.00001	NN	NN	NN	NN	27.23893	0.50054	16.35902	0.57058	NN	NN	0.02100	0.00350
1012_11_TC21_3257_N276A	1B	B	NN	NN	NN	21.65742	0.28868	0.03467	0.03405	0.00393	NN	NN	NN	NN	89.60507	6.88754	0.80414	0.01075	NN	NN	0.05400	0.81000
HIV-001428-2.42_N276A	2	C	NN	0.53909	12.40000	0.01435	0.53000	0.00155	0.01100	0.00002	NN	NN	NN	2.46747	NN	2.00477	ND	0.94445	NN	NN	0.00550	0.00240
3301.v1.c24_N276A		AC	NN	39.31180	NN	0.76253	NN	0.03244	0.20735	0.00001	NN	NN	NN	12.53858	NN	1.56525	NN	0.13680	NN	NN	0.09000	0.00340
6041.v3.c23_N276A		AC	NN	7.14739	NN	NN	NN	NN	NN	2.56698	NN	NN	NN	NN	NN	NN	NN	NN	NN	NN	0.00900	0.00290
92TH021_N276A	2	AE	NN	NN	NN	NN	NN	NN	NN	2.80218	NN	NN	NN	NN	NN	NN	NN	NN	NN	NN	0.06000	0.01000
191084 B7-19_N276D	2	A	0.11201	0.00255	0.14182	0.00439	0.01806	0.00262	0.00795	0.00024	NN	1.01321	NN	0.29781	0.02558	0.00074	0.01150	0.00127	NN	36.73057	0.02700	0.00520
SC422661.8_N276D	2	B	35.10128	NT	NN	NT	0.25366	NT	0.00585	NT	NN	ND	NN	ND	7.62172	NT	4.75373	NT	NN	ND	0.01000	NT
HIV-001428-2.42_N276D	2	C	1.79904	0.00369	0.45619	0.00137	0.01341	0.00123	0.00114	0.00001	NN	99.53962	NN	1.30816	2.16156	0.00392	5.18986	0.01404	NN	NN	0.01000	0.00230
HIV-0815.v3.c3_N276D	2	ACD	4.16580	NT	9.12986	NT	NN	NT	0.04374	NT	NN	ND	NN	ND	NN	NT	NN	NT	NN	ND	0.00780	NT
MLV			NN	NN	NN	NN	NN	NN	NN	NN	NN	NN	NN	NN	NN	NN	NN	NN	NN	NN	NN	NN

Figure S5. Pseudovirus neutralization by post-immunization VRC01^{gHL} mAbs, related to Figure 6.
Individual pseudovirus neutralization assay IC_{50} (μg/mL) values graphed in Figure 6. The indicated ΔN276 viruses were produced in HEK293T (T) or HEK293S (S) cells. Murine leukemia virus (MLV) was used as a negative control. ND = Not determined; NN = IC_{50} >100 μg/mL, no neutralization; NT = No virus titer. Data shown is an average of two experiments.
